# Prevalence and characteristics of fever in adult and paediatric patients with coronavirus disease 2019 (COVID-19): A systematic review and meta-analysis of 17515 patients

**DOI:** 10.1371/journal.pone.0249788

**Published:** 2021-04-06

**Authors:** Md Asiful Islam, Shoumik Kundu, Sayeda Sadia Alam, Tareq Hossan, Mohammad Amjad Kamal, Rosline Hassan

**Affiliations:** 1 Department of Haematology, School of Medical Sciences, Universiti Sains Malaysia, Kelantan, Malaysia; 2 Department of Biochemistry and Molecular Biology, Faculty of Biological Sciences, Jahangirnagar University, Savar, Dhaka, Bangladesh; 3 Department of Biochemistry and Molecular Biology, Cumming School of Medicine, University of Calgary, Calgary, Alberta, Canada; 4 West China School of Nursing, Frontiers Science Center for Disease-Related Molecular Network, West China Hospital, Institutes for Systems Genetics, Sichuan University, Chengdu, China; 5 King Fahd Medical Research Center, King Abdulaziz University, Jeddah, Saudi Arabia; 6 Enzymoics, Novel Global Community Educational Foundation, Hebersham, New South Wales, Australia; University of Oxford, UNITED KINGDOM

## Abstract

**Background:**

Coronavirus disease 2019 (COVID-19), a pandemic disease caused by the severe acute respiratory syndrome coronavirus 2 started to spread globally since December 2019 from Wuhan, China. Fever has been observed as one of the most common clinical manifestations, although the prevalence and characteristics of fever in adult and paediatric COVID-19 patients is inconclusive. We aimed to conduct a systematic review and meta-analysis to estimate the overall pooled prevalence of fever and chills in addition to fever characteristics (low, medium, and high temperature) in both adult and paediatric COVID-19 patients.

**Methods:**

The protocol of this systematic review and meta-analysis was registered with PROSPERO (CRD42020176327). PubMed, Scopus, ScienceDirect and Google Scholar databases were searched between 1^st^ December 2019 and 3^rd^ April 2020 without language restrictions. Both adult (≥18 years) and paediatric (<18 years) COVID-19 patients were considered eligible. We used random-effects model for the meta-analysis to obtain the pooled prevalence and risk ratio (RR) with 95% confidence intervals (CIs). Quality assessment of included studies was performed using the Joanna Briggs Institute critical appraisal tools. Heterogeneity was assessed using the *I²* statistic and Cochran’s Q test. Robustness of the pooled estimates was checked by different subgroups and sensitivity analyses.

**Results:**

We identified 2055 studies, of which 197 studies (n = 24266) were included in the systematic review and 167 studies with 17142 adults and 373 paediatrics were included in the meta-analysis. Overall, the pooled prevalence of fever in adult and paediatric COVID-19 patients were 79.43% [95% CI: 77.05–81.80, *I*^*2*^ = 95%] and 45.86% [95% CI: 35.24–56.48, *I*^*2*^ = 78%], respectively. Besides, 14.45% [95% CI: 10.59–18.32, *I*^*2*^ = 88%] of the adult COVID-19 patients were accompanied with chills. In adult COVID-19 patients, the prevalence of medium-grade fever (44.33%) was higher compared to low- (38.16%) and high-grade fever (14.71%). In addition, the risk of both low (RR: 2.34, 95% CI: 1.69–3.22, *p*<0.00001, *I*^2^ = 84%) and medium grade fever (RR: 2.79, 95% CI: 2.21–3.51, *p*<0.00001, *I*^2^ = 75%) were significantly higher compared to high-grade fever, however, there was no significant difference between low- and medium-grade fever (RR: 1.17, 95% CI: 0.94–1.44, *p* = 0.16, *I*^2^ = 87%). 88.8% of the included studies were of high-quality. The sensitivity analyses indicated that our findings of fever prevalence for both adult and paediatric patients are reliable and robust.

**Conclusions:**

The prevalence of fever in adult COVID-19 patients was high, however, 54.14% of paediatric COVID-19 patients did not exhibit fever as an initial clinical feature. Prevalence and risk of low and medium-grade fevers were higher compared to high-grade fever.

## Introduction

In December 2019, a novel coronavirus namely severe acute respiratory syndrome coronavirus-2 (SARS-CoV-2) infection outbroke in Wuhan, Hubei province, China causing coronavirus disease 2019 (COVID-19) [1]. Although it started in China, within a very short time, this infection has spread all over the world. Over 108 million people across 219 countries were infected with 2.38 million confirmed death cases until 14^th^ February 2021 [2].

In the last 17 years, two other human coronaviruses namely SARS-CoV in November 2002 and Middle East respiratory syndrome coronavirus (MERS-CoV) in April 2012 were reported to cause SARS and MERS diseases, respectively; leading to a fatal lower respiratory tract infection [3, 4]. Although SARS-CoV and MERS-CoV are both closely related to SARS-CoV-2, it is evident that SARS-CoV-2 is more infectious and spreads more rapidly than that of SARS-CoV and MERS-CoV [5]. A widespread clinical spectrum of SARS-CoV-2 infection has been observed ranging from asymptomatic, mild upper respiratory tract illness to severe viral pneumonia with respiratory failure and, death [6, 7]. Although the clinical symptoms of COVID-19 include cough, sore throat, muscle ache, shortness of breath, headache. smell dysfunction and taste disorder [7–11]; fever has been observed as the most predominant initial clinical symptom in both adult and paediatric COVID-19 patients [12, 13]. A variable degree of fever ranging from low to high-grade accompanied with or without chills has been detected in COVID-19 patients [7, 8, 14].

The prevalence and characteristics of fever in adult and paediatric COVID-19 patients is contradictory and inconclusive. A systematic review and meta-analysis can resolve the debate, aid in clinical diagnosis avoiding unnecessary delay in addition to managing COVID-19 patients in a more appropriate manner. Therefore, the objective of this systematic review and meta-analysis was to estimate the overall pooled prevalence of fever and chills in addition to fever characteristics (low, medium, and high temperature) in both adult and paediatric subjects.

## Methods

### Systematic review protocol

We conducted a systematic review and meta-analysis in accordance with the Preferred Reporting Items for Systematic Reviews and Meta-analyses (PRISMA) guideline (S1 Checklist) [15]. The protocol of this study was registered with International Prospective Register of Systematic Reviews (PROSPERO) database, registration number: CRD42020176327.

### Eligibility criteria

The objective was to identify studies published within the first four months of the COVID-19 outbreak that presented the prevalence of fever in adult (≥18 years) and paediatric (<18 years) patients with COVID-19, worldwide. There was no restriction on the study design, therefore; observational studies, clinical trials, and case series were included. In addition to the published studies, preprints were also considered if data of interest were reported. Review articles, case reports, opinions, and perspectives were excluded. Data reported by news reports and press releases or data collected from websites or databases were not considered. Nationwide studies were excluded from the meta-analysis due to the possibility of the overlapping study cohort. We handled studies from identical authors or hospitals with caution and if the study population were different, the study was included.

### Search strategy

PubMed, Scopus, ScienceDirect, and Google Scholar databases were searched to identify studies published between 1^st^ December 2019 and 3^rd^ April 2020 without language restrictions. The following search terms were searched in PubMed database (in the title and abstract of the studies) and were modified to suit other databases: COVID-19, COVID19, coronavirus, nCoV, SARS-CoV-2, SARS-CoV2, clinical, symptom, symptoms, characteristic, characteristics, feature, features, condition, conditions, comorbid, co-morbid, comorbidity, co-morbidity, comorbidities, co-morbidities, epidemiological, epidemiology, and fever. Complete details of the search strategy are in S1 Table. To ensure a robust search procedure, references of the included studies were also reviewed. Duplicate studies were excluded by using EndNote X8 software.

### Study selection

To identify eligible studies, articles of interest were screened based on the title and abstract followed by full text by four authors (MAI, SK, SSA, and TH) independently. Disagreements about inclusion were discussed and resolved by consensus.

### Data extraction

Data extraction was done by MAI and cross-checked independently by three authors (SK, SSA, and TH). Before data extraction, all non-English-language studies were translated into English using Google Translate and validated by a native speaker. When duplicate data were identified, study with the smaller sample size or incomplete data was excluded. From each eligible study, we extracted the following information into a predefined Excel spreadsheet: first author’s last name; region (country, province/municipalities/special administrative regions/city) of the participants; data collection period; COVID-19 confirmation procedure; total number of COVID-19 patients; number of female COVID-19 patients; age; age category; subgroups of COVID-19 patients; body temperature (°C); prevalence of fever, and prevalence of chills.

### Quality assessment

The quality of included studies was assessed independently by two authors (SK and SSA) using the Joanna Briggs Institute (JBI) critical appraisal tools for cross-sectional, cohort, case-control, case series, randomised controlled trials (RCTs), and non-randomised experimental studies [16]. Further, two authors (MAI and TH) validated the results of the quality assessment. The studies were classified as low-quality (high-risk of bias) if the overall score was ≤50%.

### Data analysis

Random-effects model was used to obtain the pooled prevalence and 95% confidence intervals (CIs) of fever and chills in adult and paediatric patients with COVID-19. Risk ratio (RR) with 95% CIs were used to estimate the risk of developing fever and different grades of fever in different subgroups of COVID-19 patients. Low-, medium- and high-grade fever were defined as 37·3–38·0°C, 38·1–39·0°C and >39·0°C, respectively. To assess publication bias, funnel plots presenting prevalence estimates against their sample size were constructed and the asymmetry of the funnel plot was confirmed with Egger’s test when a minimum of ten studies was available. Heterogeneity between studies was assessed using the *I²* statistic (*I²* >75% indicating substantial heterogeneity) in addition to using the Cochran’s Q test to identify the significance of heterogeneity. All the analyses and plots were generated by using metaprop codes in meta (version 4.11–0) and metafor (version 2.4–0) packages of R (version 3.6.3) in RStudio (version 1.2.5033) and RevMan (version 5.3) software [17, 18].

### Subgroup and sensitivity analyses

To assess the prevalence and risk of fever, different COVID-19 subgroups were analysed including i) low-, medium- and high-grade fever; ii) COVID-19 patients from different regions; iii) severe vs non-severe; iv) survived (recovered or discharged) vs non-survived; v) ICU vs non-ICU patients; vi) pregnant women or new mothers. To identify the source of heterogeneity and to check the robustness of the results, sensitivity analyses were performed individually for studies with adult and paediatric population through the following strategies: i) excluding small studies (n<100); ii) excluding studies with pregnant women or new mothers; iii) excluding the low-quality studies (high-risk of bias); iv) excluding studies where the confirmation method was not reported; v) excluding non-English studies, vi) excluding outlier studies, and vii) considering only cross-sectional studies. Additionally, to identify the outlier studies and the sources of heterogeneity a Galbraith plot was constructed.

## Results

**Fig 1:**
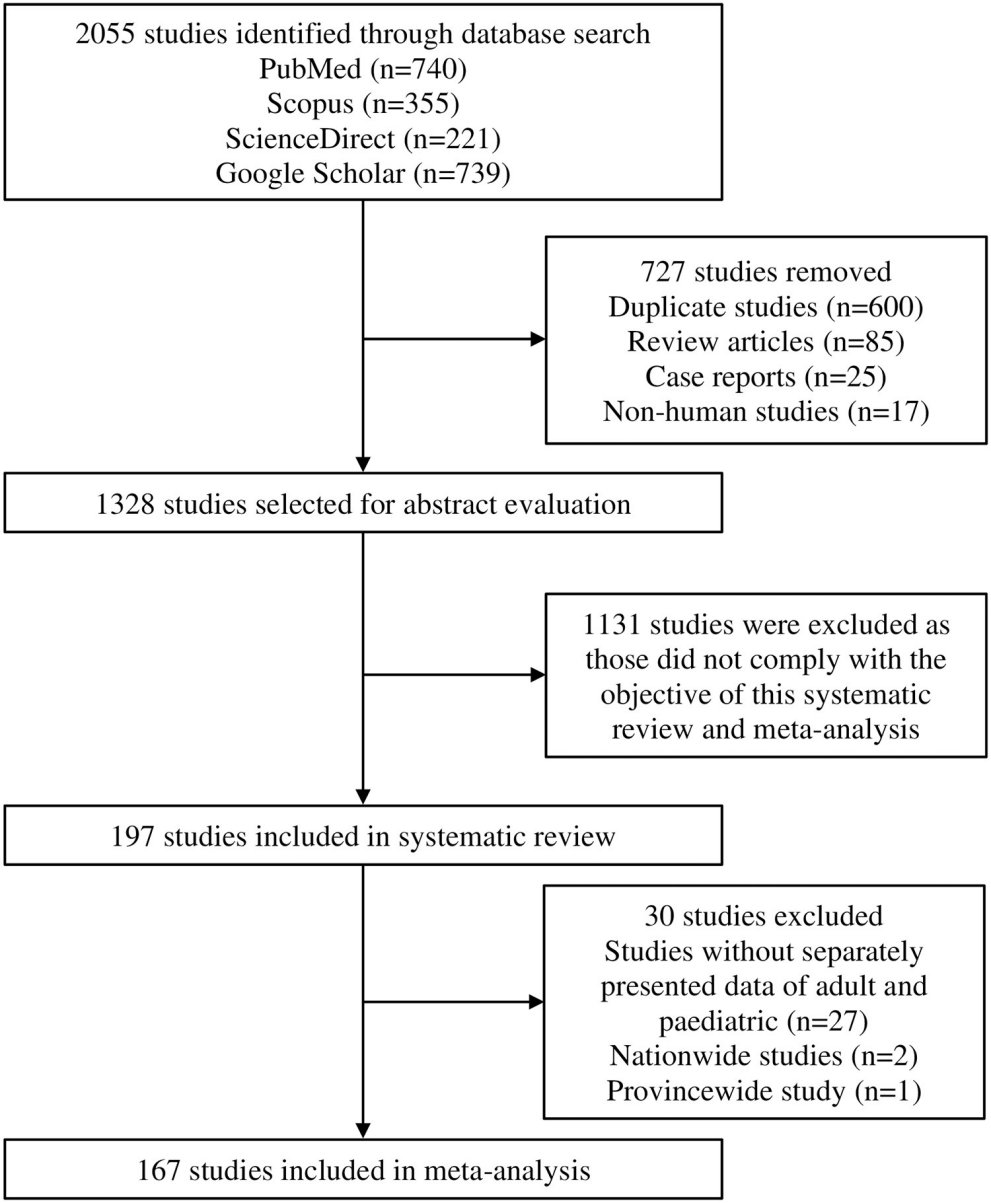
PRISMA flow diagram of study selection.

Our search initially identified 2055 studies. After removing 727 studies [duplicate studies (n = 600), review articles (n = 85), case reports (n = 25), and non-human studies (n = 17)]; titles and abstracts of 1328 studies were screened for eligibility, of which 1131 studies were excluded as those did not comply with the objective of this study. Therefore, 197 studies (n = 24266) were included in the systematic review, of which 167 studies [adult (n = 152), paediatric (n = 12), and mixed (n = 3)] were finally included in the meta-analysis (Fig 1).

Detailed characteristics and references of the included studies are presented in S2 Table. Overall, this meta-analysis reports data from 17515 COVID-19 patients (49.8% female) accumulating 17142 adults (including 270 pregnant women or new mothers) and 373 paediatrics. Ages of the adult and paediatric COVID-19 patients included in this meta-analysis ranged from 29.1±2.4 to 70.7±13.5 years and from 6.9±0.7 to 8.3±3.5 years, respectively. Studies on adult participants were from four countries including China (151 studies, n = 17078), USA (one study, n = 24), France (one study, n = 5), and Singapore (two studies, n = 35)]. All the studies on paediatric COVID-19 patients were from China. Among the included studies, 94.6% confirmed COVID-19 patients by using the reverse transcription-polymerase chain reaction (RT-PCR) method, whereas, in rest of the studies, confirmatory method was not reported.

Overall, the pooled prevalence of fever in adult and paediatric COVID-19 patients were 79.43% [95% CI: 77.05–81.80, *I*^*2*^ = 95%] and 45.86% [95% CI: 35.24–56.48, *I*^*2*^ = 78%], respectively (Table 1; S1 Fig). Prevalence of fever in Chinese, American, Singaporean, British, and French COVID-19 adult population were 79.60% [95% CI: 77.21–81.99, *I*^*2*^ = 96%], 50.00% [95% CI: 30.00–70.00], 81.80% [95% CI: 66.42–97.19, *I*^*2*^ = 33%], and 60.00% [95% CI: 17.06–100.00], respectively (Table 1; S1 Fig). Fever prevalence in adult COVID-19 patients ranged between 68.26% [95% CI: 60.46–76.07, *I*^*2*^ = 51%] and 98.63% [95% CI: 95.96–100.00] and in paediatric COVID-19 patients ranged between 42.82% [95% CI: 24.49–61.15, *I*^*2*^ = 87%] and 47.92% [95% CI: 32.95–62.88, *I*^*2*^ = 30%] in 15 Chinese provinces or municipalities (Table 1; S2 Fig).

**Table 1:** Pooled prevalence of fever in COVID-19 patients from different regions.

Regions		Fever prevalence [95% CIs] (%)	Number of studies analysed	Total number of COVID-19 patients	Heterogeneity		Publication bias, Egger’s test (*p*-value)
					*I*^*2*^	*p-*value	
Worldwide (Adult)		79.43 [77.05–81.80]	155	17142	95%	<0.0001	0.06
China (Adult)		79.60 [77.21–81.99]	151	17078	96%	<0.0001	0.05
China (Paediatric)		45.86 [35.24–56.48]	15	373	78%	<0.0001	0.0002
China provinces / municipalities	Hubei (Adult)	78.44 [75.00–81.88]	86	10069	97%	<0.0001	0.18
	Hubei (Paediatric)	42.82 [24.49–61.15]	5	209	87%	<0.0001	NA
	Zhejiang (Adult)	84.32 [77.64–91.00]	6	1812	90%	<0.0001	NA
	Shanghai (Adult)	86.10 [81.36–90.84]	10	1223	81%	<0.0001	0.37
	Jiangsu (Adult)	70.37 [61.62–79.11]	3	892	83%	0.003	NA
	Chongqing (Adult)	79.87 [73.19–86.54]	7	792	82%	<0.0001	NA
	Guangdong (Adult)	81.24 [70.38–92.10]	8	788	91%	<0.0001	NA
	Guangdong (Paediatric)	47.92 [32.95–62.88]	4	60	30%	0.23	NA
	Hunan (Adult)	68.26 [60.46–76.07]	3	301	51%	0.12	NA
	Beijing (Adult)	84.89 [80.34–89.44]	6	233	0%	0.46	NA
	Anhui (Adult)	89.66 [85.09–94.24]	4	204	12%	0.33	NA
	Hainan (Adult)	79.31 [73.67–84.95]	3	198	0%	0.97	NA
	Fujian (Adult)	76.36 [69.88–82.85]	1	165	NA	NA	NA
	Hebei (Adult)	97.30 [92.07–100.00]	1	37	NA	NA	NA
	Sichuan (Adult)	84.02 [76.31–91.73]	4	84	0%	0.42	NA
	Shandong (Adult)	98.63 [95.96–100.00]	1	73	NA	NA	NA
	Shaanxi (Adult)	94.24 [85.24–100.00]	2	41	19%	0.26	NA
USA (Adult)		50.00 [30.00–70.00]	1	24	NA	NA	NA
Singapore (Adult)		81.80 [66.42–97.19]	2	35	33%	0.22	NA
UK (Adult and paediatric)		39.71 [28.08–51.34]	1	68	NA	NA	NA
France (Adult)		60.00 [17.06–100.00]	1	5	NA	NA	NA

CIs, confidence intervals; NA, not applicable.

**Fig 2:**
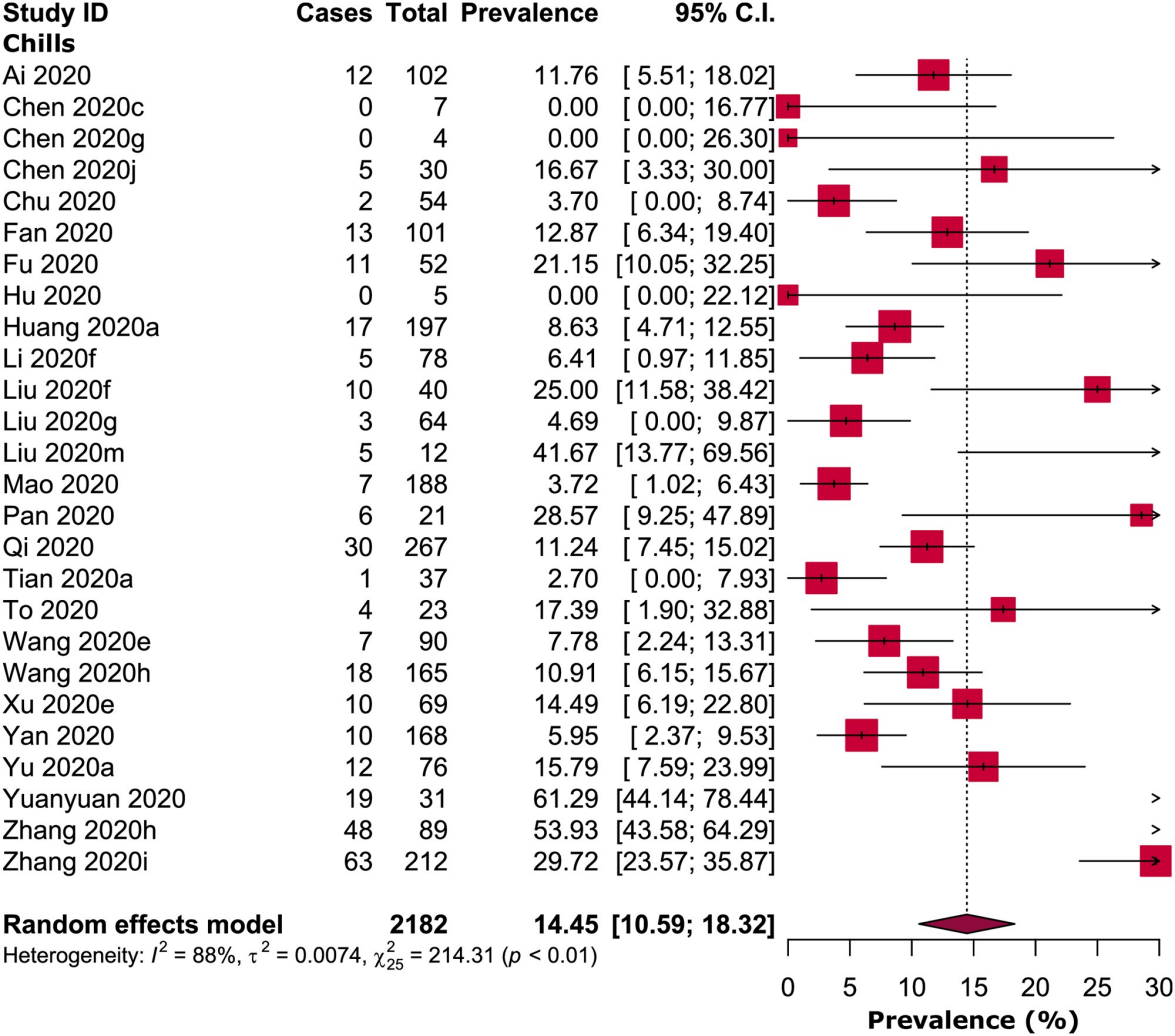
Prevalence of chills in adult COVID-19 patients.

**Fig 3:**
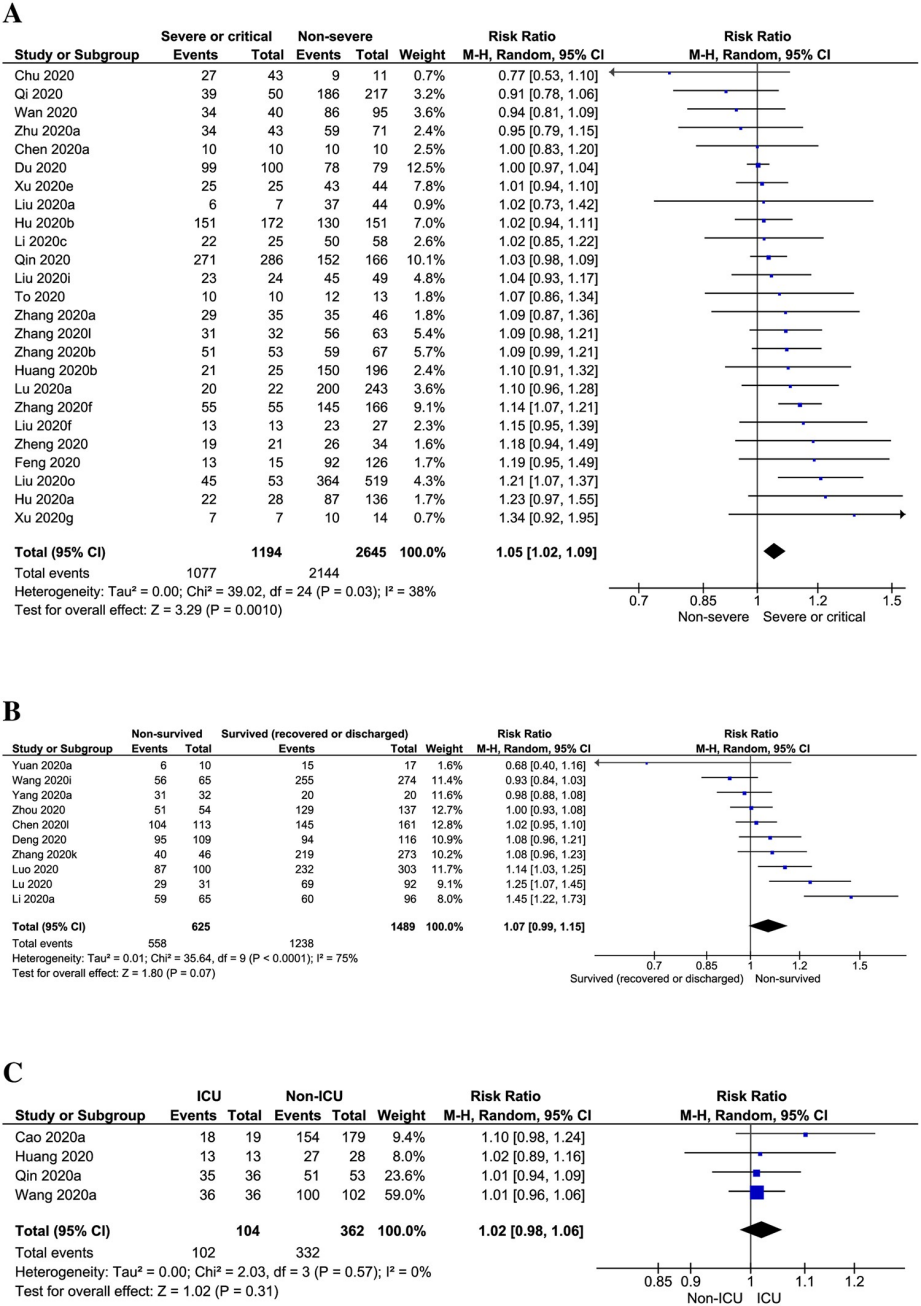
Risks of fever prevalence in (A) severe or critical vs non-severe, (B) non-survived vs survived (recovered or discharged) and (C) ICU vs non-ICU adult COVID-19 patients.

Besides fever, 14.45% [95% CI: 10.59–18.32, *I*^*2*^ = 88%] of the adult COVID-19 patients were accompanied with chills (Fig 2). Risk of fever was observed significantly higher in severe or critical COVID-19 patients when compared to non-severe COVID-19 patients (prevalence: 91.69% vs 83.85%; RR: 1.05, 95% CI: 1.02–1.09; *p =* 0.001, *I*^*2*^ = 38%) (Table 2; Fig 3; S3 Fig). There was no significant difference of fever risk in ICU vs non-ICU (RR: 1.02; 95% CI: 0.98–1.06; *p =* 0.31, *I*^*2*^ = 0%) and survived (recovered or discharged) vs non-survived COVID-19 patients (RR: 1.07, 95% CI: 0.99–1.15; *p =* 0.07, *I*^*2*^ = 75%) (Table 2; Fig 3; S3 Fig). In pregnant women or new mothers, the prevalence of fever was 56.45% [95% CI: 40.15–72.75, *I*^*2*^ = 89%] (Table 2; S3 Fig).

**Table 2:** Pooled prevalence and characteristics of fever in different subgroups of COVID-19 patients.

Subgroups of adult COVID-19 patients	Fever prevalence [95% CIs] (%)	Number of studies analysed	Total number of COVID-19 patients	Heterogeneity		Publication bias, Egger’s test (*p-*value)
				*I*^*2*^	*p-*value	
**Severe**	91.69 [89.18–94.20]	32	1678	78%	<0.0001	0.51
Low-grade fever (37.3–38.0°C)	30.27 [4.74–55.79]	7	284	97%	<0.0001	NA
Medium-grade fever (38.1–39.0°C)	43.17 [24.44–61.90]			92%	<0.0001	
High-grade fever (>39°C)	22.39 [10.51–34.28]			88%	<0.0001	
**Non-severe**	83.85 [79.50–88.21]	26	2745	91%	<0.0001	0.05
Low-grade fever (37.3–38.0°C)	36.16 [22.93–49.39]	7	431	88%	<0.0001	NA
Medium-grade fever (38.1–39.0°C)	43.90 [39.24–48.55]			0%	0.53	
High-grade fever (>39°C)	14.16 [7.99–20.33]			70%	0.002	
**Survived (recovered or discharged)**	84.17 [79.41–88.94]	17	1720	87%	<0.0001	0.75
Low-grade fever (37.3–38.0°C)	46.19 [31.54–60.83]	3	132	64%	0.06	NA
Medium-grade fever (38.1–39.0°C)	42.94 [34.23–51.65]			6%	0.34	
High-grade fever (>39°C)	8.51 [0.38–16.64]			63%	0.06	
**Non-survived**	90.13 [87.47–92.79]	13	863	43%	0.04	0.06
Low-grade fever (37.3–38.0°C)	33.65 [27.23–40.07]	3	207	0%	0.60	NA
Medium-grade fever (38.1–39.0°C)	47.93 [38.60–57.26]			47%	0.15	
High-grade fever (>39°C)	17.76 [12.16–23.35]			13%	0.31	
**ICU patients**	98.83 [96.03–100.00]	4	104	0%	0.87	NA
Low-grade fever (37.3–38.0°C)	23.08 [0.17–45.98]	1	13	NA	NA	NA
Medium-grade fever (38.1–39.0°C)	53.85 [26.75–80.95]					
High-grade fever (>39°C)	23.08 [0.17–45.98]					
**Non-ICU patients**	94.27 [88.70–99.83]	4	362	82%	0.0007	0.08
Low-grade fever (37.3–38.0°C)	18.52 [3.87–33.17]	1	27	NA	NA	NA
Medium-grade fever (38.1–39.0°C)	40.74 [22.21–59.27]					
High-grade fever (>39°C)	40.74 [22.21–59.27]					
**Pregnant women or new mothers**	56.45 [40.15–72.75]	11	270	89%	<0.0001	0.26

CIs, confidence intervals; NA, not applicable.

**Fig 4:**
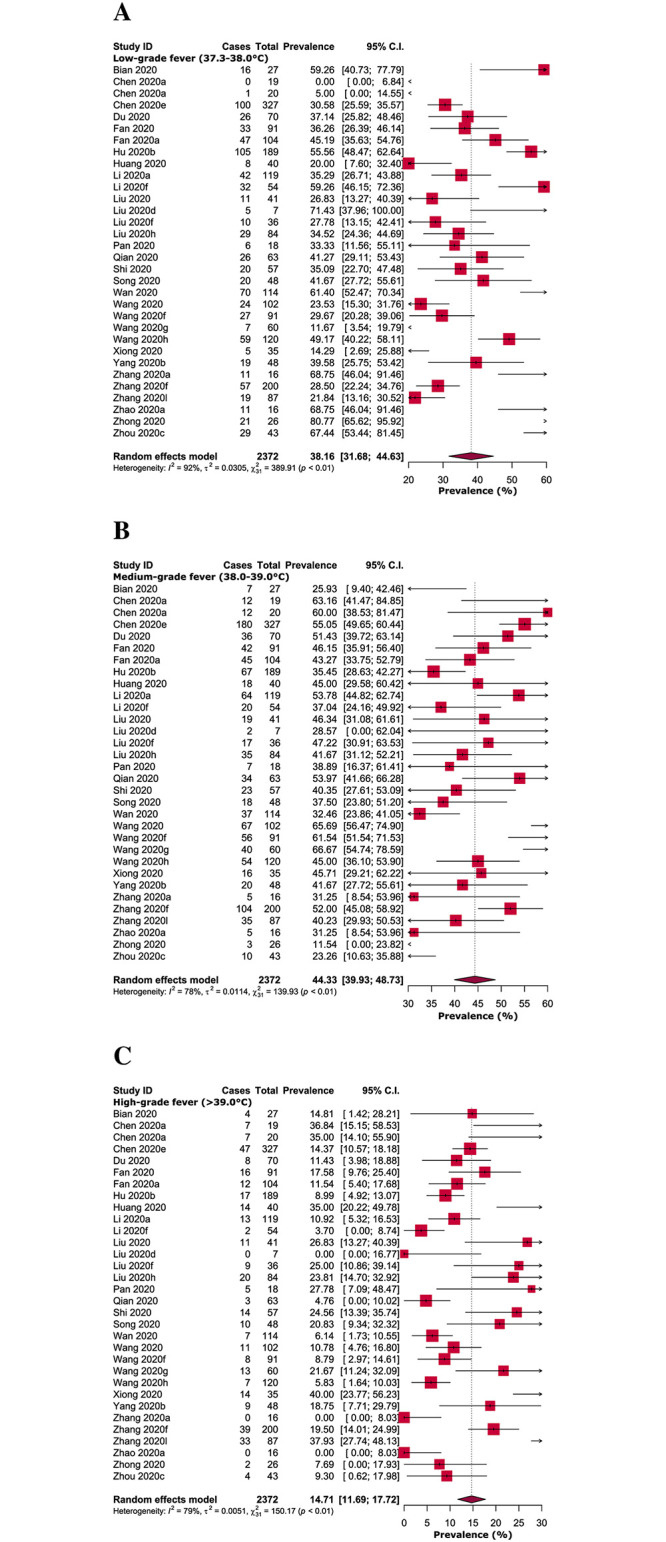
Prevalence of (A) low (37.3–38.0°C), (B) medium (38.0–39.0°C) and (C) high-grade (>39.0°C) fever in adult COVID-19 patients.

**Fig 5:**
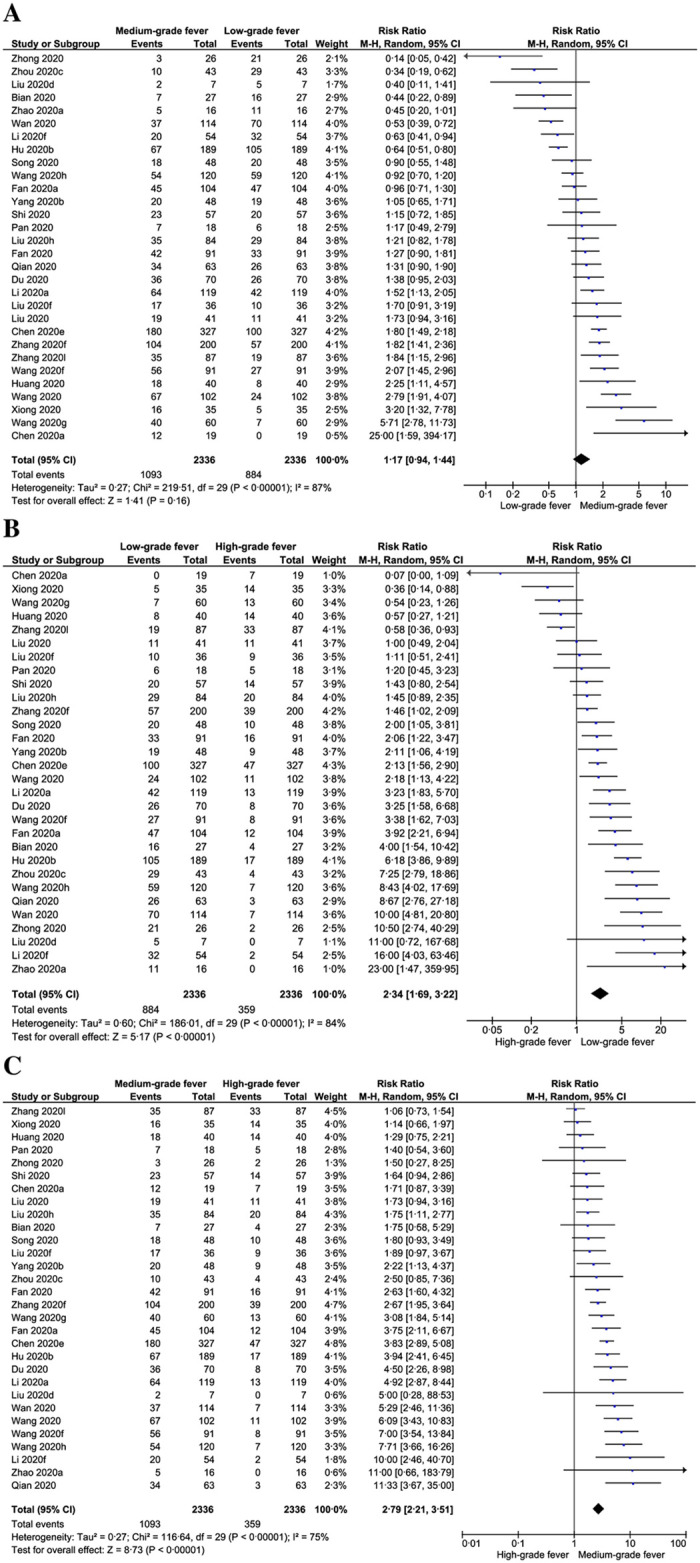
Risks of (A) low-grade fever (37·3–38·0°C) vs medium-grade fever (38·1–39·0°C), (B) high-grade fever (>39·0°C) vs low-grade fever (37·3–38·0°C), and (C) high-grade fever (>39·0°C) vs medium-grade fever (38·1–39·0°C) in adult COVID-19 patients.

In adult COVID-19 patients, among different grades of fever, the prevalence of medium-grade fever (44.33%) was higher compared to low- (38.16%) and high-grade fever (14.71%). In addition, the risk of both low (RR: 2.34, 95% CI: 1.69–3.22, *p*<0.00001) and medium grade fever (RR: 2.79, 95% CI: 2.21–3.51, *p*<0.00001) were significantly higher compared to high-grade fever, however, there was no significant difference between low- and medium-grade fever (RR: 1.17, 95% CI: 0.94–1.44, *p* = 0.16) (Figs 4 and 5; Table 3).

**Table 3:** Risk of different grades of fever in adult COVID-19 patients.

Subgroups of adult COVID-19 patients	Risk ratio [95% CIs]	*p-*value	Interpretation	Number of studies analysed	Total number of COVID-19 patients	Heterogeneity	
						*I*^*2*^	*p-*value
**Overall**							
Low vs medium-grade fever	1.17 [0.94–1.44]	0.16	Medium-grade fever higher risk than low-grade fever	30	2336	87%	<0.00001
High vs low-grade fever	2.34 [1.69–3.22]	**<0.00001**	**Low-grade fever significantly higher risk than high-grade fever**			84%	<0.00001
High vs medium-grade fever	2.79 [2.21–3.51]	**<0.00001**	**Medium-grade fever significantly higher risk than high-grade fever**			75%	<0.00001
**Severe**							
Low vs medium-grade fever	1.73 [0.59–5.03]	0.31	Medium-grade fever higher risk than low-grade fever	7	284	91%	<0.00001
High vs low-grade fever	1.14 [0.29–4.57]	0.85	low-grade fever higher risk than high-grade fever			90%	<0.00001
High vs medium-grade fever	2.05 [1.02–4.12]	**0.04**	**Medium-grade fever significantly higher risk than high-grade fever**			81%	<0.0001
**Non-severe**							
Low vs medium-grade fever	1.04 [0.79–1.37]	0.78	Medium-grade fever higher risk than low-grade fever	7	431	59%	0.02
High vs low-grade fever	2.50 [1.32–4.73]	**0.005**	**Low-grade fever significantly higher risk than high-grade fever**			77%	0.00002
High vs medium-grade fever	2.72 [1.89–3.90]	**<0.00001**	**Medium-grade fever significantly higher risk than high-grade fever**			43%	0.10
**Survived (Recovered or discharged)**							
Low vs medium-grade fever	0.92 [0.57–1.50]	0.74	Low-grade fever higher risk than medium-grade fever	3	132	63%	0.07
High vs low-grade fever	4.33 [1.02–18.45]	**0.046**	**Low-grade fever significantly higher risk than high-grade fever**			82%	0.004
High vs medium-grade fever	4.13 [1.25–13.68]	**0.02**	**Medium-grade fever significantly higher risk than high-grade fever**			74%	0.02
**Non-survived**							
Low vs medium-grade fever	1.56 [1.00–2.42]	**0.05**	**Medium-grade fever higher risk than low-grade fever**	2	150	58%	0.12
High vs low-grade fever	2.08 [1.35–3.20]	**0.0008**	**Low-grade fever significantly higher risk than high-grade fever**			0%	0.95
High vs medium-grade fever	3.15 [1.99–4.99]	**<0.00001**	**Medium-grade fever significantly higher risk than high-grade fever**			21%	0.26

CIs, confidence intervals.

In different subgroups of COVID-19 patients, the prevalence of low and medium-grade fever was found significantly higher in non-severe (prevalence: 36.16%, RR: 2.50, 95% CI: 1.32–4.73, *p =* 0.005, *I*^*2*^ = 88% and prevalence: 43.90%, RR: 2.72, 95% CI: 1.89–3.90, *p =* 0.00001, *I*^*2*^ = 0%; respectively) and non-survived adult COVID-19 patients (prevalence: 33.65%, RR: 2.08, 95% CI: 1.35–3.20, *p =* 0.0008, *I*^*2*^ = 0% and prevalence: 47.93%, RR: 3.15, 95% CI: 1.99–4.99, *p =* 0.00001, *I*^*2*^ = 47%; respectively) when compared to high-grade fever (Tables 2 and 3; S4–S13 Figs).

**Fig 6:**
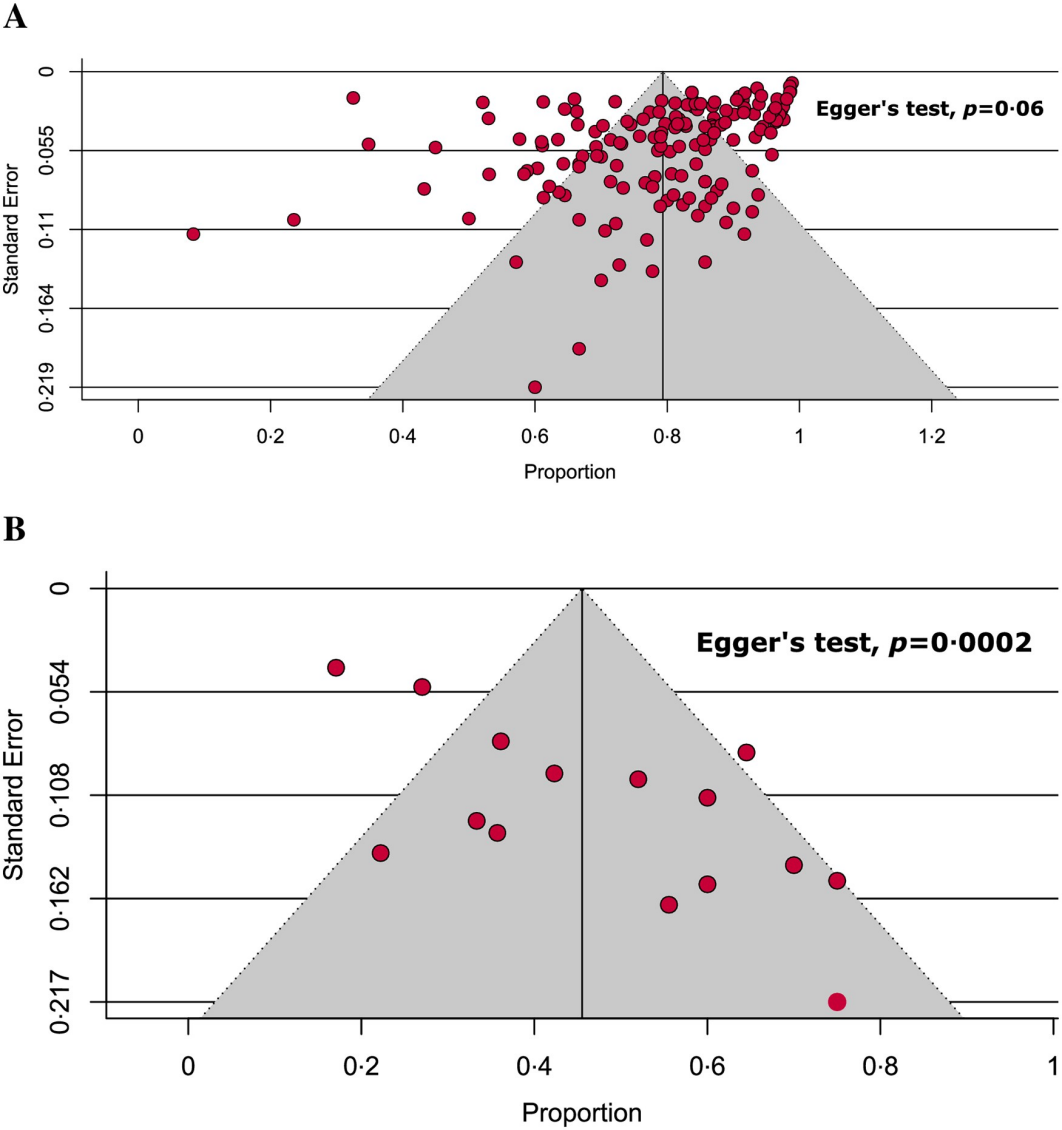
Funnel plots on (A) adult and (B) paediatric COVID-19 studies.

Detailed quality assessment of the included studies is shown in S3–S8 Tables. Briefly, 88.8% of the included studies were of high-quality (low-risk of bias); of which, none of the cohort, case series, case-control, RCTs, and non-randomized experimental studies was of low-quality and all the remaining low-quality studies (11.2%) were cross-sectional. Overall, different levels of heterogeneity (ranging from 0% to 97%) were observed during the estimation of the prevalence of fever in COVID-19 adult and paediatric patients from different regions (Table 1). Moreover, variations in the levels of heterogeneity were also observed in different subgroups ranging from 0% to 97% (Table 2). Following the visual inspection of funnel plots and Egger’s test results (Fig 6), none of the analyses on adult patients (Table 1) and subgroups (Table 2) exhibited significant publication bias, except for a single analysis on Chinese paediatric patients (Table 1).

**Fig 7:**
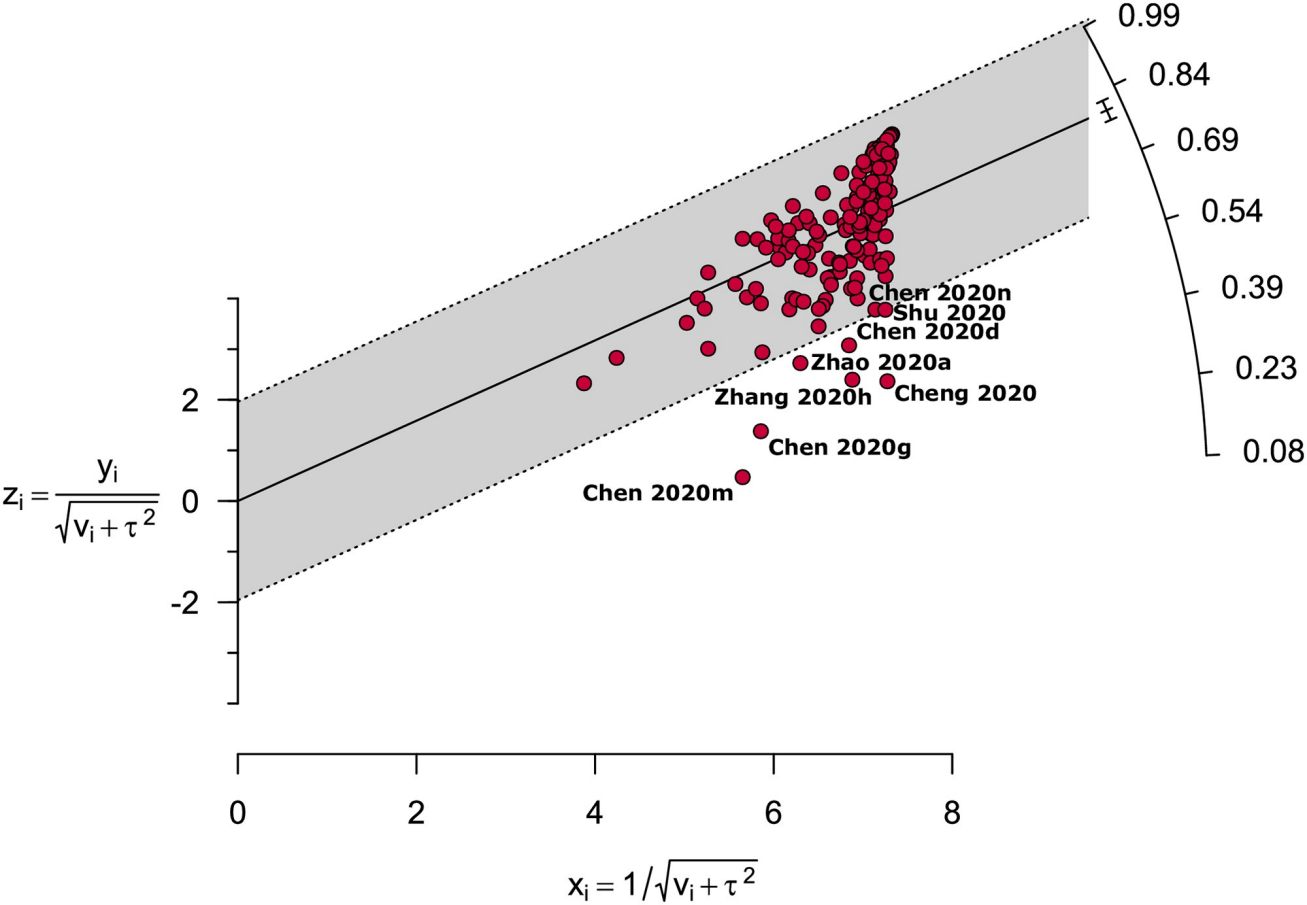
Galbraith plot identified eight outlier studies as potential sources of heterogeneity.

Sensitivity analyses on adult COVID-19 patients excluding studies on the basis of small studies, pregnant women or new mothers, low-quality studies, COVID-19 confirmation method not being reported, non-English studies, outlier studies, and considering only cross-sectional studies showed marginal differences in overall pooled prevalence with 0.7% lower, 1.6% higher, 0.4% lower, 0.04% higher, 0.04% lower, 3.2% higher, and 2.1% higher, respectively (Table 4; S14 Fig). Additionally, sensitivity analyses on paediatric population excluding low-quality studies, non-English studies, and considering only cross-sectional studies resulted in 8.9% higher, 4.2% lower, and 8.9% lower pooled prevalence, respectively (Table 4; S15 Fig). Overall, our sensitivity analyses for both adult and paediatric population indicated that the fever prevalence of both adult and paediatric patients are reliable and robust as there were no substantial changes following different strategies of sensitivity analyses. As the sources of heterogeneity, although we identified eight outlier studies from the Galbraith plot (Fig 7), performing a sensitivity analysis excluding these outlier studies could not reduce the levels of heterogeneity.

**Table 4:** Sensitivity analyses.

Strategies of Sensitivity analyses	Fever prevalence [95% CIs] (%)	Difference of pooled prevalence compared to the main result	Number of studies analysed	Total number of COVID-19 patients	Heterogeneity	
					*I*^*2*^	*p*-value
**Adults**						
Excluding small studies	78.86 [74.82–82.91]	0.7% lower	51	12735	98%	<0.0001
Excluding pregnant women or new mothers	80.72 [78.35–83.09]	1.6% higher	144	16782	95%	<0.0001
Excluding low-quality studies	79.13 [76.59–81.68]	0.4% lower	138	15922	96%	<0.0001
Excluding studies without reported COVID-19 confirmation procedure	79.77 [77.61–81.93]	0.04% higher	146	16085	94%	<0.0001
Excluding non-English studies	79.40 [76.97–81.82]	0.04% lower	149	16912	96%	<0.0001
Excluding outlier studies	81.98 [80.11–83.86]	3.2% higher	147	15469	92%	<0.0001
Considering only cross-sectional studies	81.07 [78.91–83.23]	2.1% higher	123	14100	93%	<0.0001
**Paediatrics**						
Excluding low-quality studies	49.94 [40.10–59.77]	8.9% higher	13	282	63%	0.003
Excluding non-English studies	43.93 [33.51–54.35]	4.2% lower	14	342	74%	<0.0001
Considering only cross-sectional studies	41.76 [28.28–55.24]	8.9% lower	9	285	82%	<0.0001

CIs, confidence intervals.

## Discussion

Based on the findings of this meta-analysis, the prevalence of fever was estimated to be 79.43% in symptomatic adult COVID-19 patients, which is less common than SARS (99–100%) [19, 20], however, similar to MERS (77%, meta-analysis result) [21]. We estimated the prevalence of fever in paediatric COVID-19 subjects to be 45.86%, however, from the systematic literature search-based studies, the mean prevalence in the paediatric MERS and SARS subjects was 6.45% and 98%, respectively [22, 23]. Even though the prevalence of fever in COVID-19 paediatric subjects is higher than MERS and lower than SARS paediatric population, nevertheless, more than half of the COVID-19 paediatric patients did not show fever as an initial symptom. Therefore, for the clinical confirmation of paediatric COVID-19 symptomatic subjects, fever should not be considered as the only initial symptom. To avoid delaying in diagnosis, history of exposure to COVID-19 patients, especially household exposure and other clinical manifestations including cough, expectoration, polypnea, chest tightness, diarrhoea should be considered as well [24–26].

Our meta-analysis estimated fever prevalence in severe or critical COVID-19 patients as 91.69%. In severe or critical MERS patients, the prevalence of fever was observed as 71% [27], whereas fever was predominant in 95.7% of the severe or critical SARS patients [28]. Similar to severe or critical vs non-severe COVID-19 patients, body temperature was also detected higher in severe or critical patients with SARS than that of non-severe patients [29]. Similar to our findings, risk of fever was observed high in non-survived patients with MERS compared to survived patients (79.1% vs 93.9%, *p =* 0.04) [30]. Additionally, alike our findings on COVID-19, body temperature was higher in non-survived MERS patients compared to that in survived MERS patients [31, 32]. The prevalence of fever in ICU vs non-ICU SARS (95.7% vs 89.9%) [28] and COVID-19 patients from our meta-analysis (98.83 vs 94.27) were quite similar.

Pregnancy data on SARS and MERS is very limited. From our meta-analysis, we observed 56.45% of the pregnant women or new mothers with COVID-19 presented with fever. In contrast, 100% of pregnant women or new mothers with SARS [33, 34] and 80–100% with MERS [35, 36] exhibited fever. As less than half of the pregnant women or new mothers with COVID-19 did not exhibit fever as an initial symptom, other clinical manifestations observed in pregnant women or new mothers such as cough, fatigue, dyspnoea, and myalgia should also be considered [37–41].

Medium to high-grade fever was predominantly detected in patients with SARS [29, 42–45] and MERS [4, 46, 47]; findings from our meta-analysis indicate that both low and medium-grade fever is clearly prevalent, not high-grade fever in COVID-19 patients. We detected chills in only 14.45% of the adult COVID-19 patients, whereas, in SARS [48] and MERS [33, 49], chills were estimated to be 59.3% and 87%-92%, respectively. Therefore, while chills were considered as a distinctive clinical feature in SARS and MERS diagnosis, chills are not possibly a typical clinical manifestation for COVID-19 diagnosis.

Our study has several strengths. This meta-analysis is the first, to our knowledge, to comprehensively investigate the prevalence and characteristics of fever in adult and paediatric COVID-19 patients. This meta-analysis was conducted with a large number of studies and hence including a large number of participants, resulting in more robust estimates. We included both English and non-English-language articles, and the non-English-language articles do not seem to affect overall estimates in this meta-analysis. Majority of the included studies confirmed COVID-19 subjects by using the RT-PCR technique which strengthens our findings. Majority of the analyses did not represent significant publication bias demonstrating that we were unlikely to have missed studies that could have altered the findings. All the conducted sensitivity analyses generated similar results to the main findings indicating the robustness of the meta-analysis results. Based on the quality assessments, approximately 89% of the studies were of high methodological quality (low-risk of bias) which ensured a reliable result. Nevertheless, there are several notable limitations. Based on the search strategy and considered time period, this meta-analysis could include only 3% studies conducted outside China, therefore, the prevalence may not represent at a global scale and generalisation of the findings should be done with care. Most of the analyses generated substantial degrees of heterogeneity even though we tried to identify the sources of heterogeneity by constructing subgroup, sensitivity analyses and Galbraith plot.

Due to the absence of fever as an initial clinical presentation, diagnosis of COVID-19 may be initially missed. Identification of suspected patients with COVID-19 would be difficult when the patients are asymptomatic [12, 50], especially without fever manifestation. In such cases, other manifestations should be considered. As fever seems to be an important initial symptom of COVID-19, to halt the spread of the disease, a digital infrared thermal imaging system with maximum accuracy could be considered to screen mass suspected COVID-19 patients with a history of contact to COVID-19-positive individuals or history of intra and intercountry travelling or visiting in hospitals or clinics [51]. Temperature-monitoring campaign and fever hotline which were quite successful during the SARS outbreak could be considered for identifying suspected COVID-19 subjects and take immediate actions [52, 53].

## Conclusions

In conclusion, the findings from this meta-analysis represent the most comprehensive and robust currently available evidence of fever prevalence in adult and paediatric COVID-19 patients. We estimated the prevalence of fever reported during admission as 79.43% in adult and 45.86% in paediatric COVID-19 patients in addition to 14.45% chills. Prevalence and risk of low and medium-grade fevers were higher compared to high-grade fever. Therefore, fever should be considered as one of the most common initial clinical symptoms for adults. In case of paediatric COVID-19 patients, fever should not be considered as the only initial symptom, rather, history of exposure to COVID-19 patients, especially household exposure and other clinical manifestations including cough, expectoration, polypnea, chest tightness, diarrhoea should be considered as well. We hope that these results will assist in the decision making of patients, clinicians, and policymakers.

## Supporting information

S1 Checklist Preferred Reporting Items for Systematic Reviews and Meta-Analyses (PRISMA). (DOCX)

S1 Fig Prevalence of fever in (A) adult, (B) paediatric COVID-19 patients and adult patients from (C) China, (D) USA, (E) Singapore, (F) UK, and (G) France. (PDF)

S2 Fig Prevalence of fever in adult COVID-19 patients from (A) Hubei, (B) Hubei (Paediatric), (C) Zhejiang, (D) Shanghai, (E) Jiangsu, (F) Chongqing, (G) Guangdong, (H) Guangdong (Paediatric), (I) Hunan, (J) Beijing, (K) Anhui, (L) Hainan, (M) Fujian, (N) Hebei, (O) Sichuan, (P) Shandong, and (Q) Shaanxi. (PDF)

S3 Fig Prevalence of fever in (A) severe or critical, (B) non-severe, (C) survived (recovered or discharged), (D) non-survived, (E) ICU, (F) non-ICU, and (G) pregnant women or new mothers with COVID-19. (PDF)

S4 Fig Prevalence of (A) low-grade (37·3–38·0°C), (B) medium-grade (38·1–39·0°C), and (C) high-grade fever (>39·0°C) in severe or critical COVID-19 adult patients. (PDF)

S5 Fig Prevalence of (A) low-grade (37·3–38·0°C), (B) medium-grade (38·1–39·0°C), and (C) high-grade fever (>39·0°C) in non-severe COVID-19 adult patients. (PDF)

S6 Fig Prevalence of (A) low-grade (37·3–38·0°C), (B) medium-grade (38·1–39·0°C), and (C) high-grade fever (>39·0°C) in survived (recovered or discharged) adult COVID-19 patients. (PDF)

S7 Fig Prevalence of (A) low-grade (37·3–38·0°C), (B) medium-grade (38·1–39·0°C), and (C) high-grade fever (>39·0°C) in non-survived adult COVID-19 patients. (PDF)

S8 Fig Prevalence of (A) low-grade (37·3–38·0°C), (B) medium-grade (38·1–39·0°C), and (C) high-grade fever (>39·0°C) in adult COVID-19 ICU patients. (PDF)

S9 Fig Prevalence of (A) low-grade (37·3–38·0°C), (B) medium-grade (38·1–39·0°C), and (C) high-grade fever (>39·0°C) in adult COVID-19 non-ICU patients. (PDF)

S10 Fig Risks of (A) low-grade fever (37·3–38·0°C) vs medium-grade fever (38·1–39·0°C), (B) high-grade fever (>39·0°C) vs low-grade fever (37·3–38·0°C), (C) and high-grade fever (>39·0°C) vs medium-grade fever (38·1–39·0°C) in adult severe or critical COVID-19 patients. (PDF)

S11 Fig Risks of (A) low-grade fever (37·3–38·0°C) vs medium-grade fever (38·1–39·0°C), (B) high-grade fever (>39·0°C) vs low-grade fever (37·3–38·0°C), and (C) high-grade fever (>39·0°C) vs medium-grade fever (38·1–39·0°C) in adult non-severe COVID-19 patients. (PDF)

S12 Fig Risks of (A) low-grade fever (37·3–38·0°C) vs medium-grade fever (38·1–39·0°C), (B) high-grade fever (>39·0°C) vs low-grade fever (37·3–38·0°C), and (C) high-grade fever (>39·0°C) vs medium-grade fever (38·1–39·0°C) in survived (recovered or discharged) adult COVID-19 patients. (PDF)

S13 Fig Risks of (A) low-grade fever (37·3–38·0°C) vs medium-grade fever (38·1–39·0°C), (B) high-grade fever (>39·0°C) vs low-grade fever (37·3–38·0°C), and (C) high-grade fever (>39·0°C) vs medium-grade fever (38·1–39·0°C) in non-survived adult COVID-19 patients. (PDF)

S14 Fig Sensitivity analyses: Prevalence of fever in adult COVID-19 patients (A) excluding small studies (n<100), (B) excluding pregnant women or new mothers, (C) excluding low-quality studies, (D) excluding studies without confirmation method being reported, (E) excluding non-English studies, (F) excluding outlier studies, and (G) considering only cross-sectional studies. (PDF)

S15 Fig Sensitivity analyses: Prevalence of fever in paediatric COVID-19 patients (A) excluding low-quality studies, (B) excluding non-English studies, and (C) considering only cross-sectional studies. (PDF)

S1 Table Keywords used to search databases. (DOCX)

S2 Table Major characteristics of the included studies. (DOCX)

S3 Table Quality assessment of the included cross-sectional studies. (DOCX)

S4 Table Quality assessment of the included cohort studies. (DOCX)

S5 Table Quality assessment of the included case series. (DOCX)

S6 Table Quality assessment of the included randomised controlled trials. (DOCX)

S7 Table Quality assessment of the included case-control studies. (DOCX)

S8 Table Quality assessment of the included non-randomised experimental studies. (DOCX)
