# Supplementary material for: Prevalence and characteristics of fever in adult and paediatric patients with coronavirus disease 2019 (COVID-19): A systematic review and meta-analysis of 17515 patients
Source: PLoS One. 2021 Apr 6;16(4):e0249788. doi: 10.1371/journal.pone.0249788 (PMC8023501; doi:10.1371/journal.pone.0249788)
Supplement: S1 Fig — (PDF) [file pone.0249788.s002.pdf]

A

Study ID	Cases	Total	Prevalence	95% C.I.
**Overall (Adult)**				
Bhatraju 2020	12	24	50.00	[30.00; 70.00]
Bian 2020	27	28	96.43	[89.55; 100.00]
Cao 2020	182	199	91.46	[87.57; 95.34]
Cao 2020a	172	198	86.87	[82.16; 91.57]
Chang 2020a	14	16	87.50	[71.30; 100.00]
Chen 2020	20	31	64.52	[47.67; 81.36]
Chen 2020a	20	20	100.00	[93.48; 100.00]
Chen 2020b	217	249	87.15	[82.99; 91.31]
Chen 2020c	7	9	77.78	[50.62; 100.00]
Chen 2020d	40	89	44.94	[34.61; 55.28]
Chen 2020e	327	534	61.24	[57.10; 65.37]
Chen 2020f	82	99	82.83	[75.40; 90.26]
Chen 2020g	4	17	23.53	[3.37; 43.69]
Chen 2020i	36	37	97.30	[92.07; 100.00]
Chen 2020j	28	30	93.33	[84.41; 100.00]
Chen 2020k	85	123	69.11	[60.94; 77.27]
Chen 2020l	249	274	90.88	[87.47; 94.29]
Chen 2020m	0	5	0.00	[0.00; 22.12]
Chen 2020n	125	236	52.97	[46.60; 59.33]
Cheng 2020	213	655	32.52	[28.93; 36.11]
Cheng 2020a	8	11	72.73	[46.41; 99.05]
Cheng 2020b	64	111	57.66	[48.47; 66.85]
Chu 2020	36	54	66.67	[54.09; 79.24]
Chuan 2020	25	32	78.12	[63.80; 92.45]
Chung 2020	14	21	66.67	[46.50; 86.83]
Cui 2020	25	35	71.43	[56.46; 86.39]
Deng 2020	189	225	84.00	[79.21; 88.79]
Diao 2020	4	6	66.67	[28.95; 100.00]
Ding 2020	5	5	100.00	[77.88; 100.00]
Du 2020	177	179	98.88	[97.34; 100.00]
Du 2020a	32	53	60.38	[47.21; 73.55]
Fan 2020	91	101	90.10	[84.27; 95.92]
Fan 2020a	104	148	70.27	[62.91; 77.63]
Feng 2020	105	141	74.47	[67.27; 81.67]
Fu 2020	45	52	86.54	[77.26; 95.82]
Han 2020	94	108	87.04	[80.70; 93.37]
Han 2020a	14	17	82.35	[64.23; 100.00]
He 2020	19	31	61.29	[44.14; 78.44]
Hou 2020	12	14	85.71	[67.38; 100.00]
Hu 2020a	109	164	66.46	[59.24; 73.69]
Hu 2020b	271	323	83.90	[79.89; 87.91]
Huang 2020	40	41	97.56	[92.84; 100.00]
Huang 2020b	171	221	77.38	[71.86; 82.89]
Huang 2020c	34	36	94.44	[86.96; 100.00]
Huang 2020d	32	34	94.12	[86.21; 100.00]
Ji 2020	32	33	96.97	[91.12; 100.00]
Jin 2020	545	651	83.72	[80.88; 86.55]
Lei 2020	28	29	96.55	[89.91; 100.00]
Lei 2020b	16	20	80.00	[62.47; 97.53]
Lescure 2020	3	5	60.00	[17.06; 100.00]
Li 2020	12	17	70.59	[48.93; 92.25]
Li 2020a	119	161	73.91	[67.13; 80.70]
Li 2020b	34	47	72.34	[59.55; 85.13]
Li 2020c	72	83	86.75	[79.45; 94.04]
Li 2020d	67	77	87.01	[79.50; 94.52]
Li 2020e	15	18	83.33	[66.12; 100.00]
Li 2020f	54	78	69.23	[58.99; 79.47]
Liang 2020	18	21	85.71	[70.75; 100.00]
Liao 2020	22	30	73.33	[57.51; 89.16]
Liu 2020	41	51	80.39	[69.50; 91.29]
Liu 2020a	43	51	84.31	[74.33; 94.29]
Liu 2020b	193	291	66.32	[60.89; 71.75]
Liu 2020c	13	15	86.67	[69.46; 100.00]
Liu 2020d	7	10	70.00	[41.60; 98.40]
Liu 2020e	30	51	58.82	[45.32; 72.33]
Liu 2020f	36	40	90.00	[80.70; 99.30]
Liu 2020g	43	64	67.19	[55.68; 78.69]
Liu 2020h	112	137	81.75	[75.28; 88.22]
Liu 2020i	68	73	93.15	[87.36; 98.95]
Liu 2020j	10	13	76.92	[54.02; 99.83]
Liu 2020k	90	109	82.57	[75.45; 89.69]
Liu 2020l	57	78	73.08	[63.23; 82.92]
Liu 2020o	409	620	65.97	[62.24; 69.70]
Liu 2020p	44	56	78.57	[67.82; 89.32]
Lu 2020	98	123	79.67	[72.56; 86.79]
Lu 2020a	220	265	83.02	[78.50; 87.54]
Lu 2020b	69	91	75.82	[67.03; 84.62]
Luo 2020	319	403	79.16	[75.19; 83.12]
Mao 2020	159	188	84.57	[79.41; 89.74]
Miao 2020	49	62	79.03	[68.90; 89.17]
Min 2020	23	30	76.67	[61.53; 91.80]
Mo 2020	126	155	81.29	[75.15; 87.43]
Nie 2020	21	33	63.64	[47.22; 80.05]
Pan 2020	18	21	85.71	[70.75; 100.00]
Pung 2020	15	17	88.24	[72.92; 100.00]
Qi 2020	225	267	84.27	[79.90; 88.64]
Qian 2020	65	91	71.43	[62.15; 80.71]
Qin 2020	423	452	93.58	[91.33; 95.84]
Qin 2020a	86	89	96.63	[92.88; 100.00]
Qiu 2020a	66	104	63.46	[54.21; 72.72]
Shi 2020	59	81	72.84	[63.15; 82.53]
Shi 2020a	82	101	81.19	[73.57; 88.81]
Shu 2020	284	545	52.11	[47.92; 56.30]
Song 2020	49	51	96.08	[90.75; 100.00]
Su 2020	8	14	57.14	[31.22; 83.07]
Tang 2020a	72	73	98.63	[95.96; 100.00]
Tian 2020a	23	37	62.16	[46.54; 77.79]
To 2020	22	23	95.65	[87.32; 100.00]
Wan 2020	120	135	88.89	[83.59; 94.19]
Wang 2020	107	114	93.86	[89.45; 98.27]
Wang 2020a	136	138	98.55	[96.56; 100.00]
Wang 2020d	11	11	100.00	[88.69; 100.00]
Wang 2020e	55	90	61.11	[51.04; 71.18]
Wang 2020f	91	110	82.73	[75.66; 89.79]
Wang 2020g	60	69	86.96	[79.01; 94.90]
Wang 2020h	126	165	76.36	[69.88; 82.85]
Wang 2020i	311	339	91.74	[88.81; 94.67]
Wang 2020j	42	60	70.00	[58.40; 81.60]
Wei 2020	61	100	61.00	[51.44; 70.56]
Wei 2020a	72	84	85.71	[78.23; 93.20]
Wu 2020b	72	91	79.12	[70.77; 87.47]
Xie 2020	8	9	88.89	[68.36; 100.00]
Xiong 2020	36	42	85.71	[75.13; 96.30]
Xu 2020	28	48	58.33	[44.39; 72.28]
Xu 2020b	34	51	66.67	[53.73; 79.60]
Xu 2020e	68	69	98.55	[95.73; 100.00]
Xu 2020g	17	21	80.95	[64.16; 97.75]
Yang 2020	7	7	100.00	[83.23; 100.00]
Yang 2020a	51	52	98.08	[94.34; 100.00]
Yang 2020b	47	55	85.45	[76.14; 94.77]
Yang 2020c	42	44	95.45	[89.30; 100.00]
Yao 2020	53	55	96.36	[91.42; 100.00]
Young 2020	13	18	72.22	[51.53; 92.91]
Yu 2020	6	7	85.71	[59.79; 100.00]
Yu 2020a	67	76	88.16	[80.89; 95.42]
Yuan 2020a	21	27	77.78	[62.10; 93.46]
Yuanyuan 2020	25	31	80.65	[66.74; 94.55]
Zeng 2020	218	338	64.50	[59.40; 69.60]
Zhang 2020	64	82	78.05	[69.09; 87.01]
Zhang 2020a	64	81	79.01	[70.14; 87.88]
Zhang 2020b	110	120	91.67	[86.72; 96.61]
Zhang 2020c	11	13	84.62	[65.00; 100.00]
Zhang 2020d	13	14	92.86	[79.37; 100.00]
Zhang 2020f	200	221	90.50	[86.63; 94.36]
Zhang 2020g	540	645	83.72	[80.87; 86.57]
Zhang 2020h	31	89	34.83	[24.93; 44.73]
Zhang 2020i	167	212	78.77	[73.27; 84.28]
Zhang 2020j	23	28	82.14	[67.96; 96.33]
Zhang 2020k	259	319	81.19	[76.90; 85.48]
Zhang 2020l	87	95	91.58	[85.99; 97.16]
Zhao 2020	15	19	78.95	[60.62; 97.28]
Zhao 2020a	16	37	43.24	[27.28; 59.21]
Zhao 2020b	36	56	64.29	[51.74; 76.84]
Zhao 2020g	71	80	88.75	[81.83; 95.67]
Zhao 2020h	331	459	72.11	[68.01; 76.22]
Zheng 2020	45	55	81.82	[71.62; 92.01]
Zhong 2020	26	49	53.06	[39.09; 67.03]
Zhou 2020	180	191	94.24	[90.94; 97.54]
Zhou 2020a	54	62	87.10	[78.75; 95.44]
Zhou 2020b	216	254	85.04	[80.65; 89.43]
Zhou 2020c	43	62	69.35	[57.88; 80.83]
Zhu 2020	27	32	84.38	[71.79; 96.96]
Zhu 2020a	93	114	81.58	[74.46; 88.70]
Zhu 2020b	6	6	100.00	[80.92; 100.00]
Zhu 2020c	9	10	90.00	[71.41; 100.00]
**Random effects model**		**17142**	**79.43**	**[77.05; 81.80]**

Heterogeneity: I^2 = 95%, τ^2 = 0.0194, χ^2_{154} = 3361.50 (p = 0)

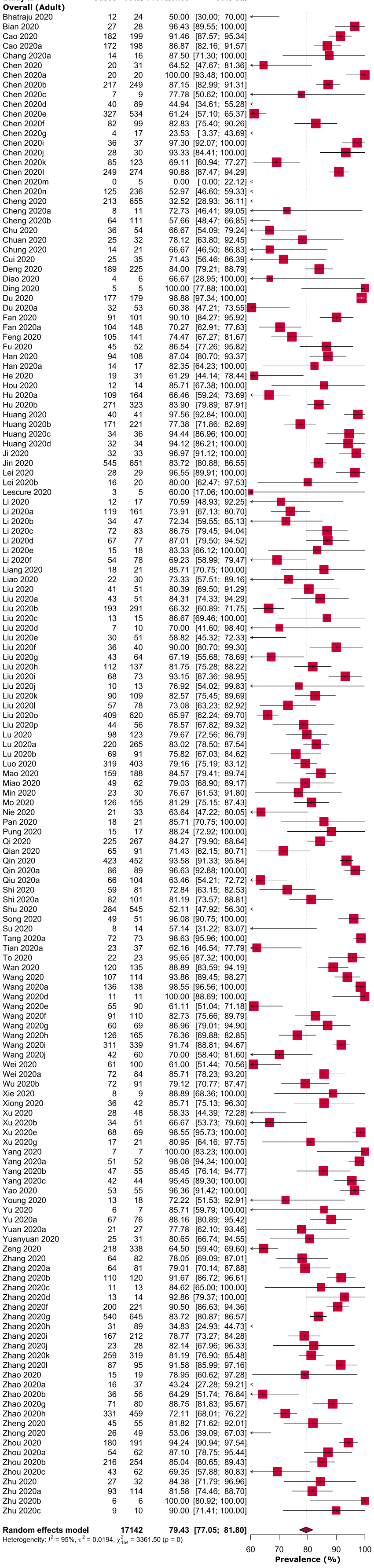

B

Study ID	Cases	Total	Prevalence	95% C.I.
**Overall (Paediatric)**				
Du 2020a	5	14	35.71	[10.61; 60.81]
Feng 2020a	5	15	33.33	[9.48; 57.19]
Jiehao 2020	6	10	60.00	[29.64; 90.36]
Liu 2020e	3	4	75.00	[32.57; 100.00]
Qiu 2020	13	36	36.11	[20.42; 51.80]
Su 2020	2	9	22.22	[0.00; 49.38]
Sun 2020	6	8	75.00	[44.99; 100.00]
Tang 2020	11	26	42.31	[23.32; 61.30]
Wang 2020b	20	31	64.52	[47.67; 81.36]
Wu 2020c	20	74	27.03	[16.91; 37.15]
Xia 2020	12	20	60.00	[38.53; 81.47]
Xu 2020d	7	10	70.00	[41.60; 98.40]
Yu 2020b	14	82	17.07	[8.93; 25.22]
Zheng 2020a	13	25	52.00	[32.42; 71.58]
Zhou 2020d	5	9	55.56	[23.09; 88.02]
**Random effects model**		**373**	**45.86**	**[35.24; 56.48]**

Heterogeneity: I^2 = 78%, τ^2 = 0.0302, χ^2_{14} = 62.55 (p < 0.01)

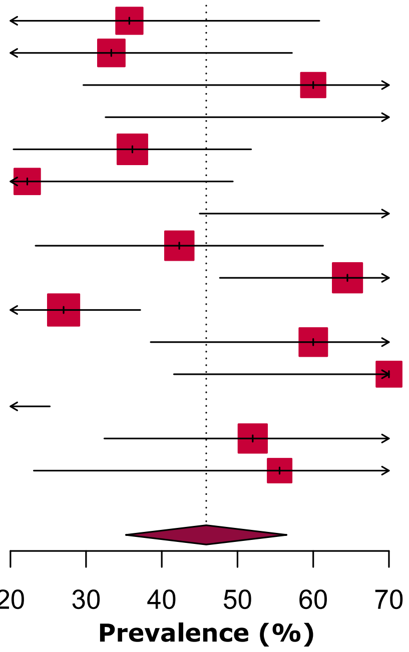

C

Study ID	Cases	Total	Prevalence	95% C.I.
**Chinese (Adult)**				
Bian 2020	27	28	96.43	[89.55; 100.00]
Cao 2020	182	199	91.46	[87.57; 95.34]
Cao 2020a	172	198	86.87	[82.16; 91.57]
Chang 2020a	14	16	87.50	[71.30; 100.00]
Chen 2020	20	31	64.52	[47.67; 81.36]
Chen 2020a	20	20	100.00	[93.48; 100.00]
Chen 2020b	217	249	87.15	[82.99; 91.31]
Chen 2020c	7	9	77.78	[50.62; 100.00]
Chen 2020d	40	89	44.94	[34.61; 55.28]
Chen 2020e	327	534	61.24	[57.10; 65.37]
Chen 2020f	82	99	82.83	[75.40; 90.26]
Chen 2020g	4	17	23.53	[3.37; 43.69]
Chen 2020i	36	37	97.30	[92.07; 100.00]
Chen 2020j	28	30	93.33	[84.41; 100.00]
Chen 2020k	85	123	69.11	[60.94; 77.27]
Chen 2020l	249	274	90.88	[87.47; 94.29]
Chen 2020m	0	5	0.00	[0.00; 22.12]
Chen 2020n	125	236	52.97	[46.60; 59.33]
Cheng 2020	213	655	32.52	[28.93; 36.11]
Cheng 2020a	8	11	72.73	[46.41; 99.05]
Cheng 2020b	64	111	57.66	[48.47; 66.85]
Chu 2020	36	54	66.67	[54.09; 79.24]
Chuan 2020	25	32	78.12	[63.80; 92.45]
Chung 2020	14	21	66.67	[46.50; 86.83]
Cui 2020	25	35	71.43	[56.46; 86.39]
Deng 2020	189	225	84.00	[79.21; 88.79]
Diao 2020	4	6	66.67	[28.95; 100.00]
Ding 2020	5	5	100.00	[77.88; 100.00]
Du 2020	177	179	98.88	[97.34; 100.00]
Du 2020a	32	53	60.38	[47.21; 73.55]
Fan 2020	91	101	90.10	[84.27; 95.92]
Fan 2020a	104	148	70.27	[62.91; 77.63]
Feng 2020	105	141	74.47	[67.27; 81.67]
Fu 2020	45	52	86.54	[77.26; 95.82]
Han 2020	94	108	87.04	[80.70; 93.37]
Han 2020a	14	17	82.35	[64.23; 100.00]
He 2020	19	31	61.29	[44.14; 78.44]
Hou 2020	12	14	85.71	[67.38; 100.00]
Hu 2020a	109	164	66.46	[59.24; 73.69]
Hu 2020b	271	323	83.90	[79.89; 87.91]
Huang 2020	40	41	97.56	[92.84; 100.00]
Huang 2020b	171	221	77.38	[71.86; 82.89]
Huang 2020c	34	36	94.44	[86.96; 100.00]
Huang 2020d	32	34	94.12	[86.21; 100.00]
Ji 2020	32	33	96.97	[91.12; 100.00]
Jin 2020	545	651	83.72	[80.88; 86.55]
Lei 2020	28	29	96.55	[89.91; 100.00]
Lei 2020b	16	20	80.00	[62.47; 97.53]
Li 2020	12	17	70.59	[48.93; 92.25]
Li 2020a	119	161	73.91	[67.13; 80.70]
Li 2020b	34	47	72.34	[59.55; 85.13]
Li 2020c	72	83	86.75	[79.45; 94.04]
Li 2020d	67	77	87.01	[79.50; 94.52]
Li 2020e	15	18	83.33	[66.12; 100.00]
Li 2020f	54	78	69.23	[58.99; 79.47]
Liang 2020	18	21	85.71	[70.75; 100.00]
Liao 2020	22	30	73.33	[57.51; 89.16]
Liu 2020	41	51	80.39	[69.50; 91.29]
Liu 2020a	43	51	84.31	[74.33; 94.29]
Liu 2020b	193	291	66.32	[60.89; 71.75]
Liu 2020c	13	15	86.67	[69.46; 100.00]
Liu 2020d	7	10	70.00	[41.60; 98.40]
Liu 2020e	30	51	58.82	[45.32; 72.33]
Liu 2020f	36	40	90.00	[80.70; 99.30]
Liu 2020g	43	64	67.19	[55.68; 78.69]
Liu 2020h	112	137	81.75	[75.28; 88.22]
Liu 2020i	68	73	93.15	[87.36; 98.95]
Liu 2020j	10	13	76.92	[54.02; 99.83]
Liu 2020k	90	109	82.57	[75.45; 89.69]
Liu 2020l	57	78	73.08	[63.23; 82.92]
Liu 2020o	409	620	65.97	[62.24; 69.70]
Liu 2020p	44	56	78.57	[67.82; 89.32]
Lu 2020	98	123	79.67	[72.56; 86.79]
Lu 2020a	220	265	83.02	[78.50; 87.54]
Lu 2020b	69	91	75.82	[67.03; 84.62]
Luo 2020	319	403	79.16	[75.19; 83.12]
Mao 2020	159	188	84.57	[79.41; 89.74]
Miao 2020	49	62	79.03	[68.90; 89.17]
Min 2020	23	30	76.67	[61.53; 91.80]
Mo 2020	126	155	81.29	[75.15; 87.43]
Nie 2020	21	33	63.64	[47.22; 80.05]
Pan 2020	18	21	85.71	[70.75; 100.00]
Qi 2020	225	267	84.27	[79.90; 88.64]
Qian 2020	65	91	71.43	[62.15; 80.71]
Qin 2020	423	452	93.58	[91.33; 95.84]
Qin 2020a	86	89	96.63	[92.88; 100.00]
Qiu 2020a	66	104	63.46	[54.21; 72.72]
Shi 2020	59	81	72.84	[63.15; 82.53]
Shi 2020a	82	101	81.19	[73.57; 88.81]
Shu 2020	284	545	52.11	[47.92; 56.30]
Song 2020	49	51	96.08	[90.75; 100.00]
Su 2020	8	14	57.14	[31.22; 83.07]
Tang 2020a	72	73	98.63	[95.96; 100.00]
Tian 2020a	23	37	62.16	[46.54; 77.79]
To 2020	22	23	95.65	[87.32; 100.00]
Wan 2020	120	135	88.89	[83.59; 94.19]
Wang 2020	107	114	93.86	[89.45; 98.27]
Wang 2020a	136	138	98.55	[96.56; 100.00]
Wang 2020d	11	11	100.00	[88.69; 100.00]
Wang 2020e	55	90	61.11	[51.04; 71.18]
Wang 2020f	91	110	82.73	[75.66; 89.79]
Wang 2020g	60	69	86.96	[79.01; 94.90]
Wang 2020h	126	165	76.36	[69.88; 82.85]
Wang 2020i	311	339	91.74	[88.81; 94.67]
Wang 2020j	42	60	70.00	[58.40; 81.60]
Wei 2020	61	100	61.00	[51.44; 70.56]
Wei 2020a	72	84	85.71	[78.23; 93.20]
Wu 2020b	72	91	79.12	[70.77; 87.47]
Xie 2020	8	9	88.89	[68.36; 100.00]
Xiong 2020	36	42	85.71	[75.13; 96.30]
Xu 2020	28	48	58.33	[44.39; 72.28]
Xu 2020b	34	51	66.67	[53.73; 79.60]
Xu 2020e	68	69	98.55	[95.73; 100.00]
Xu 2020g	17	21	80.95	[64.16; 97.75]
Yang 2020	7	7	100.00	[83.23; 100.00]
Yang 2020a	51	52	98.08	[94.34; 100.00]
Yang 2020b	47	55	85.45	[76.14; 94.77]
Yang 2020c	42	44	95.45	[89.30; 100.00]
Yao 2020	53	55	96.36	[91.42; 100.00]
Yu 2020	6	7	85.71	[59.79; 100.00]
Yu 2020a	67	76	88.16	[80.89; 95.42]
Yuan 2020a	21	27	77.78	[62.10; 93.46]
Yuanyuan 2020	25	31	80.65	[66.74; 94.55]
Zeng 2020	218	338	64.50	[59.40; 69.60]
Zhang 2020	64	82	78.05	[69.09; 87.01]
Zhang 2020a	64	81	79.01	[70.14; 87.88]
Zhang 2020b	110	120	91.67	[86.72; 96.61]
Zhang 2020c	11	13	84.62	[65.00; 100.00]
Zhang 2020d	13	14	92.86	[79.37; 100.00]
Zhang 2020f	200	221	90.50	[86.63; 94.36]
Zhang 2020g	540	645	83.72	[80.87; 86.57]
Zhang 2020h	31	89	34.83	[24.93; 44.73]
Zhang 2020i	167	212	78.77	[73.27; 84.28]
Zhang 2020j	23	28	82.14	[67.96; 96.33]
Zhang 2020k	259	319	81.19	[76.90; 85.48]
Zhang 2020l	87	95	91.58	[85.99; 97.16]
Zhao 2020	15	19	78.95	[60.62; 97.28]
Zhao 2020a	16	37	43.24	[27.28; 59.21]
Zhao 2020b	36	56	64.29	[51.74; 76.84]
Zhao 2020g	71	80	88.75	[81.83; 95.67]
Zhao 2020h	331	459	72.11	[68.01; 76.22]
Zheng 2020	45	55	81.82	[71.62; 92.01]
Zhong 2020	26	49	53.06	[39.09; 67.03]
Zhou 2020	180	191	94.24	[90.94; 97.54]
Zhou 2020a	54	62	87.10	[78.75; 95.44]
Zhou 2020b	216	254	85.04	[80.65; 89.43]
Zhou 2020c	43	62	69.35	[57.88; 80.83]
Zhu 2020	27	32	84.38	[71.79; 96.96]
Zhu 2020a	93	114	81.58	[74.46; 88.70]
Zhu 2020b	6	6	100.00	[80.92; 100.00]
Zhu 2020c	9	10	90.00	[71.41; 100.00]
**Random effects model**		**17078**	**79.60**	**[77.21; 81.99]**

Heterogeneity: $I^2 = 96\%$, $\tau^2 = 0.0193$, $\chi^2_{150} = 3345.86$ ($p = 0$)

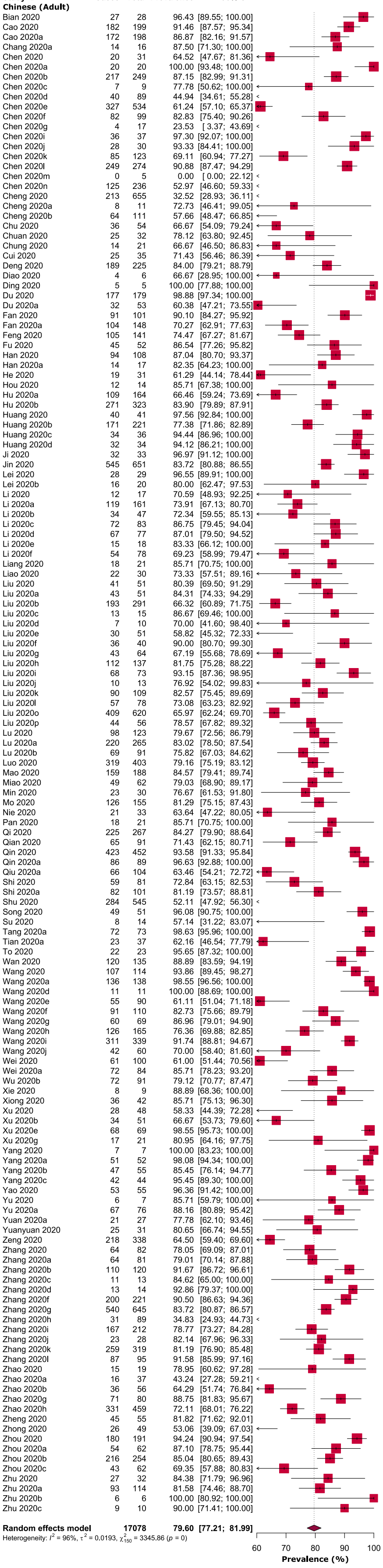

D

Study ID	Cases	Total	Prevalence	95% C.I.
**USA (Adult)**				
Bhatraju 2020	12	24	50.00	[30; 70]

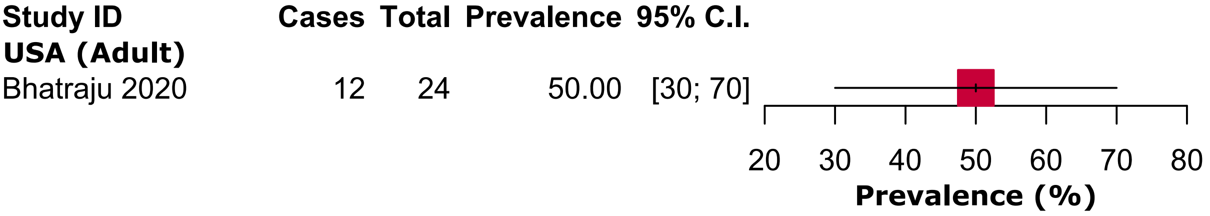

E

Study ID	Cases	Total	Prevalence	95% C.I.
**Singapore (Adult)**				
Pung 2020	15	17	88.24	[72.92; 100.00]
Young 2020	13	18	72.22	[51.53; 92.91]
**Random effects model**		**35**	**81.80**	**[66.42; 97.19]**

Heterogeneity: I^2 = 33%, τ^2 = 0.0042, χ^2_1 = 1.49 (p = 0.22)

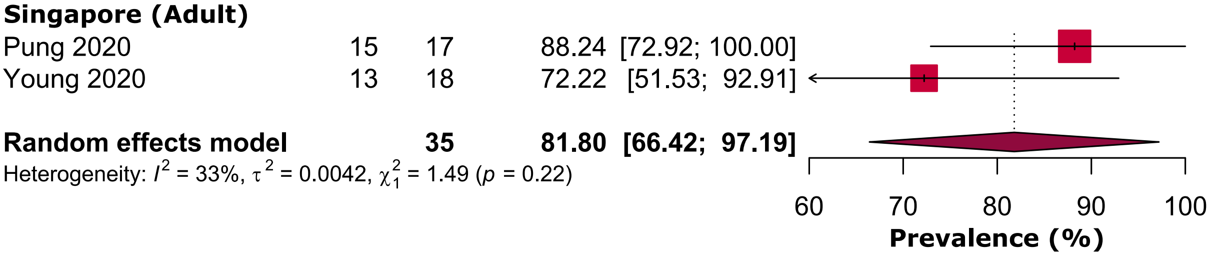

F

Study ID	Cases	Total	Prevalence	95% C.I.
**UK (Adult)**				
Easom 2020	27	68	39.71	[28.08; 51.34]

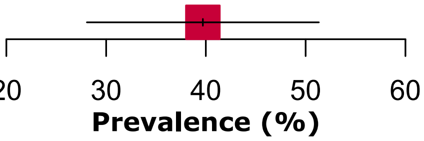

G

Study ID	Cases	Total	Prevalence	95% C.I.
**France (Adult)**				
Lescure 2020	3	5	60.00	[17.06; 100]

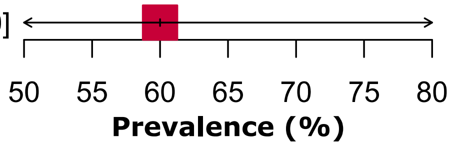

S1 Fig. Prevalence of fever in (A) adult, (B) paediatric COVID-19 patients and adult patients from (C) China, (D) USA, (E) Singapore, (F) UK, and (G) France.
