# Supplementary material for: Prevalence and characteristics of fever in adult and paediatric patients with coronavirus disease 2019 (COVID-19): A systematic review and meta-analysis of 17515 patients
Source: PLoS One. 2021 Apr 6;16(4):e0249788. doi: 10.1371/journal.pone.0249788 (PMC8023501; doi:10.1371/journal.pone.0249788)
Supplement: S2 Fig — (PDF) [file pone.0249788.s003.pdf]

A

Study ID	Cases	Total	Prevalence	95% C.I.
**Hubei (Adult)**				
Cao 2020	182	199	91.46	[87.57; 95.34]
Chen 2020	20	31	64.52	[47.67; 81.36]
Chen 2020a	20	20	100.00	[93.48; 100.00]
Chen 2020c	7	9	77.78	[50.62; 100.00]
Chen 2020d	40	89	44.94	[34.61; 55.28]
Chen 2020e	327	534	61.24	[57.10; 65.37]
Chen 2020f	82	99	82.83	[75.40; 90.26]
Chen 2020g	4	17	23.53	[3.37; 43.69]
Chen 2020j	28	30	93.33	[84.41; 100.00]
Chen 2020k	85	123	69.11	[60.94; 77.27]
Chen 2020l	249	274	90.88	[87.47; 94.29]
Chen 2020m	0	5	0.00	[0.00; 22.12]
Chen 2020n	125	236	52.97	[46.60; 59.33]
Cheng 2020	213	655	32.52	[28.93; 36.11]
Cheng 2020b	64	111	57.66	[48.47; 66.85]
Chu 2020	36	54	66.67	[54.09; 79.24]
Cui 2020	25	35	71.43	[56.46; 86.39]
Deng 2020	189	225	84.00	[79.21; 88.79]
Ding 2020	5	5	100.00	[77.88; 100.00]
Du 2020	177	179	98.88	[97.34; 100.00]
Fan 2020	91	101	90.10	[84.27; 95.92]
Han 2020	94	108	87.04	[80.70; 93.37]
Han 2020a	14	17	82.35	[64.23; 100.00]
He 2020	19	31	61.29	[44.14; 78.44]
Hou 2020	12	14	85.71	[67.38; 100.00]
Hu 2020b	271	323	83.90	[79.89; 87.91]
Huang 2020	40	41	97.56	[92.84; 100.00]
Huang 2020c	34	36	94.44	[86.96; 100.00]
Huang 2020d	32	34	94.12	[86.21; 100.00]
Lei 2020	28	29	96.55	[89.91; 100.00]
Li 2020a	119	161	73.91	[67.13; 80.70]
Li 2020d	67	77	87.01	[79.50; 94.52]
Liu 2020c	13	15	86.67	[69.46; 100.00]
Liu 2020e	30	51	58.82	[45.32; 72.33]
Liu 2020f	36	40	90.00	[80.70; 99.30]
Liu 2020g	43	64	67.19	[55.68; 78.69]
Liu 2020h	112	137	81.75	[75.28; 88.22]
Liu 2020k	90	109	82.57	[75.45; 89.69]
Liu 2020l	57	78	73.08	[63.23; 82.92]
Lu 2020	98	123	79.67	[72.56; 86.79]
Lu 2020b	69	91	75.82	[67.03; 84.62]
Luo 2020	319	403	79.16	[75.19; 83.12]
Min 2020	23	30	76.67	[61.53; 91.80]
Mo 2020	126	155	81.29	[75.15; 87.43]
Nie 2020	21	33	63.64	[47.22; 80.05]
Pan 2020	18	21	85.71	[70.75; 100.00]
Qian 2020	65	91	71.43	[62.15; 80.71]
Qin 2020	423	452	93.58	[91.33; 95.84]
Qin 2020a	86	89	96.63	[92.88; 100.00]
Shi 2020	59	81	72.84	[63.15; 82.53]
Shi 2020a	82	101	81.19	[73.57; 88.81]
Shu 2020	284	545	52.11	[47.92; 56.30]
Tang 2020a	72	73	98.63	[95.96; 100.00]
Wang 2020	107	114	93.86	[89.45; 98.27]
Wang 2020a	136	138	98.55	[96.56; 100.00]
Wang 2020e	55	90	61.11	[51.04; 71.18]
Wang 2020f	91	110	82.73	[75.66; 89.79]
Wang 2020g	60	69	86.96	[79.01; 94.90]
Wang 2020i	311	339	91.74	[88.81; 94.67]
Wang 2020j	42	60	70.00	[58.40; 81.60]
Wei 2020	61	100	61.00	[51.44; 70.56]
Wei 2020a	72	84	85.71	[78.23; 93.20]
Xiong 2020	36	42	85.71	[75.13; 96.30]
Xu 2020	28	48	58.33	[44.39; 72.28]
Yang 2020a	51	52	98.08	[94.34; 100.00]
Yao 2020	53	55	96.36	[91.42; 100.00]
Yu 2020	6	7	85.71	[59.79; 100.00]
Yuan 2020a	21	27	77.78	[62.10; 93.46]
Yuanyuan 2020	25	31	80.65	[66.74; 94.55]
Zhang 2020	64	82	78.05	[69.09; 87.01]
Zhang 2020a	64	81	79.01	[70.14; 87.88]
Zhang 2020b	110	120	91.67	[86.72; 96.61]
Zhang 2020f	200	221	90.50	[86.63; 94.36]
Zhang 2020h	31	89	34.83	[24.93; 44.73]
Zhang 2020i	167	212	78.77	[73.27; 84.28]
Zhang 2020j	23	28	82.14	[67.96; 96.33]
Zhang 2020k	259	319	81.19	[76.90; 85.48]
Zhang 2020l	87	95	91.58	[85.99; 97.16]
Zhao 2020a	16	37	43.24	[27.28; 59.21]
Zheng 2020	45	55	81.82	[71.62; 92.01]
Zhong 2020	26	49	53.06	[39.09; 67.03]
Zhou 2020	180	191	94.24	[90.94; 97.54]
Zhou 2020a	54	62	87.10	[78.75; 95.44]
Zhou 2020b	216	254	85.04	[80.65; 89.43]
Zhu 2020a	93	114	81.58	[74.46; 88.70]
Zhu 2020c	9	10	90.00	[71.41; 100.00]
**Random effects model**		**10069**	**78.44**	**[75.00; 81.88]**

Heterogeneity: I^2 = 97%, τ^2 = 0.0238, χ^2_{85} = 2692.44 (p = 0)

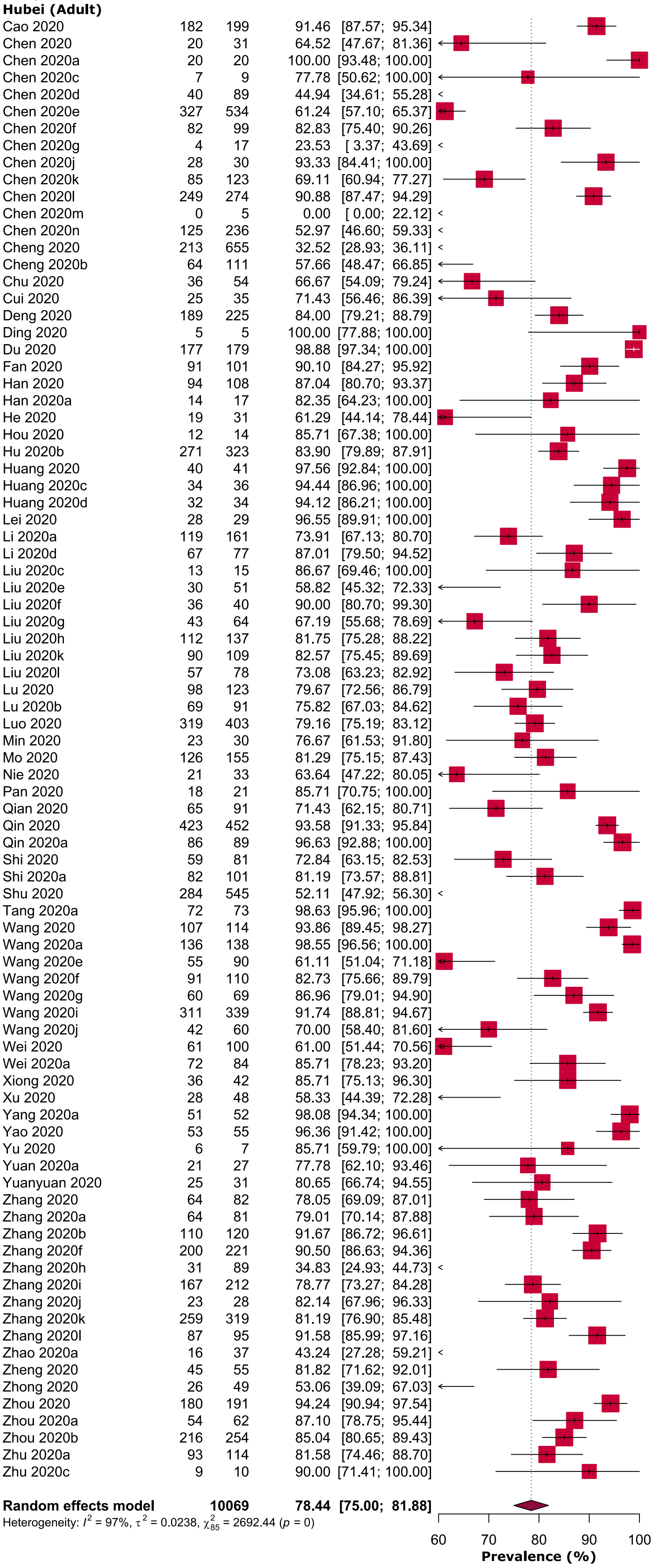

B

Study ID	Cases	Total	Prevalence	95% C.I.
**Hubei (Paediatric)**				
Sun 2020	6	8	75.00	[44.99; 100.00]
Wu 2020c	20	74	27.03	[16.91; 37.15]
Xia 2020	12	20	60.00	[38.53; 81.47]
Yu 2020b	14	82	17.07	[8.93; 25.22]
Zheng 2020a	13	25	52.00	[32.42; 71.58]
**Random effects model**		**209**	**42.82**	**[24.49; 61.15]**

Heterogeneity: I^2 = 87%, τ^2 = 0.0350, χ^2_4 = 30.67 (p < 0.01)

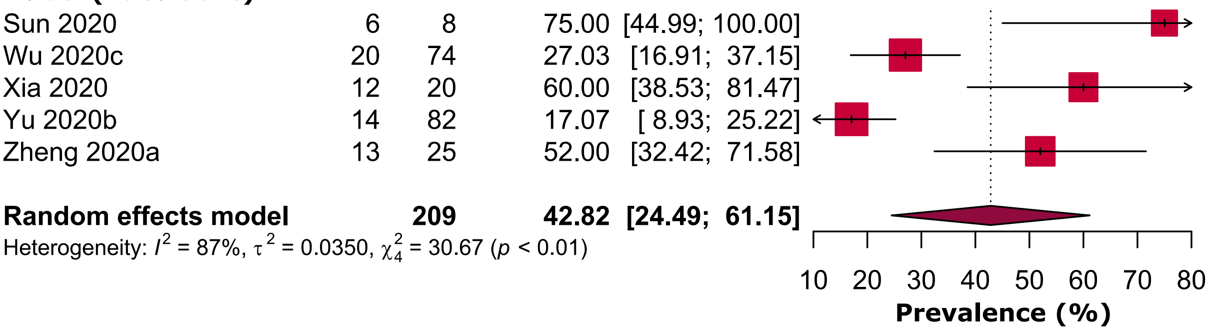

C

Study ID	Cases	Total	Prevalence	95% C.I.
**Zhejiang (Adult)**				
Ji 2020	32	33	96.97	[91.12; 100.00]
Jin 2020	545	651	83.72	[80.88; 86.55]
Liu 2020d	7	10	70.00	[41.60; 98.40]
Zhang 2020d	13	14	92.86	[79.37; 100.00]
Zhang 2020g	540	645	83.72	[80.87; 86.57]
Zhao 2020h	331	459	72.11	[68.01; 76.22]
**Random effects model**		**1812**	**84.32**	**[77.64; 91.00]**

Heterogeneity: I^2 = 90%, τ^2 = 0.0051, χ^2_5 = 52.31 (p < 0.01)

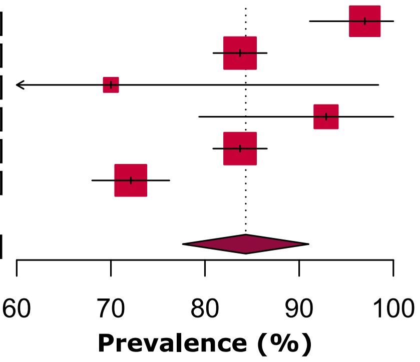

D

Study ID	Cases	Total	Prevalence	95% C.I.
**Shanghai (Adult)**				
Cao 2020a	172	198	86.87	[82.16; 91.57]
Chen 2020b	217	249	87.15	[82.99; 91.31]
Cheng 2020a	8	11	72.73	[46.41; 99.05]
Fan 2020a	104	148	70.27	[62.91; 77.63]
Lu 2020a	220	265	83.02	[78.50; 87.54]
Mao 2020	159	188	84.57	[79.41; 89.74]
Miao 2020	49	62	79.03	[68.90; 89.17]
Song 2020	49	51	96.08	[90.75; 100.00]
Yang 2020	7	7	100.00	[83.23; 100.00]
Yang 2020c	42	44	95.45	[89.30; 100.00]
**Random effects model**		**1223**	**86.10**	**[81.36; 90.84]**

Heterogeneity: I^2 = 81%, τ^2 = 0.0041, χ^2_9 = 47.74 (p < 0.01)

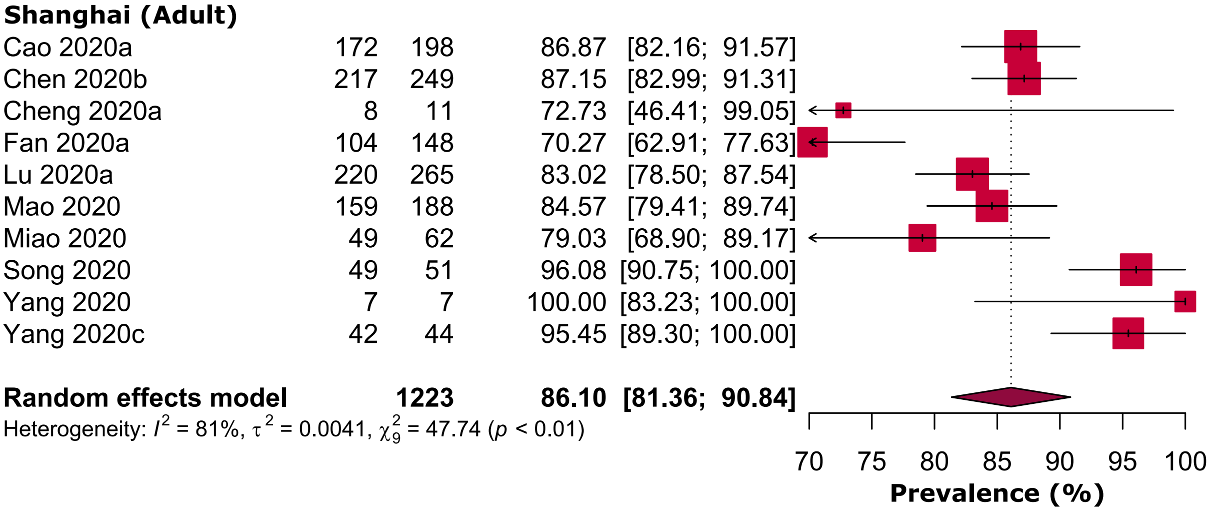

E

Study ID	Cases	Total	Prevalence	95% C.I.
**Jiangsu (Adult)**				
Huang 2020b	171	221	77.38	[71.86; 82.89]
Liu 2020o	409	620	65.97	[62.24; 69.70]
Xu 2020b	34	51	66.67	[53.73; 79.60]
**Random effects model**		**892**	**70.37**	**[61.62; 79.11]**

Heterogeneity: $I^2 = 83\%$, $\tau^2 = 0.0046$, $\chi^2_2 = 11.46$ ($p < 0.01$)

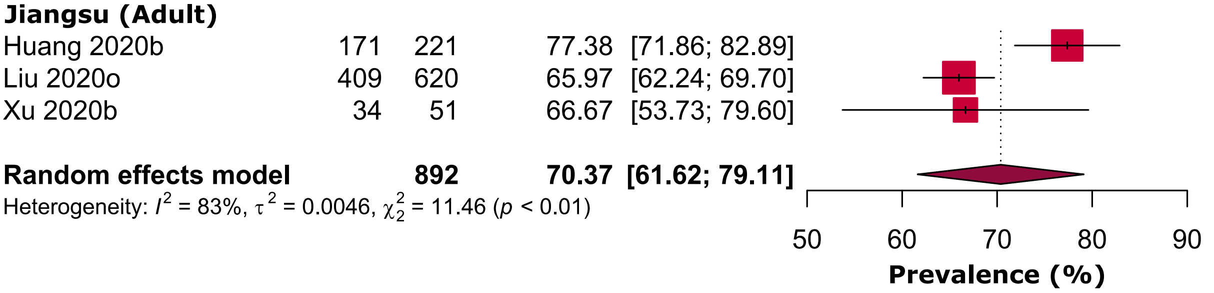

F

Study ID	Cases	Total	Prevalence	95% C.I.
**Chongqing (Adult)**				
Hu 2020a	109	164	66.46	[59.24; 73.69]
Li 2020c	72	83	86.75	[79.45; 94.04]
Liao 2020	22	30	73.33	[57.51; 89.16]
Liu 2020a	43	51	84.31	[74.33; 94.29]
Qi 2020	225	267	84.27	[79.90; 88.64]
Wan 2020	120	135	88.89	[83.59; 94.19]
Zhou 2020c	43	62	69.35	[57.88; 80.83]
**Random effects model**		**792**	**79.87**	**[73.19; 86.54]**

Heterogeneity: $I^2 = 82\%$, $\tau^2 = 0.0061$, $\chi^2_6 = 32.87$ ($p < 0.01$)

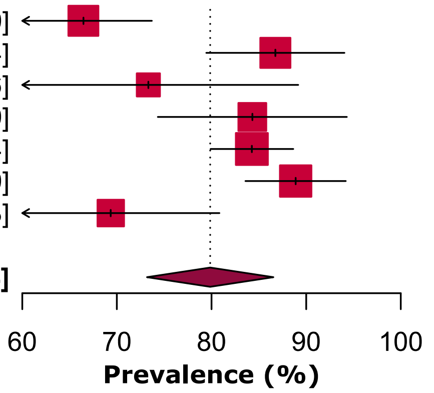

G

Study ID	Cases	Total	Prevalence	95% C.I.
**Guangdong (Adult)**				
Lei 2020b	16	20	80.00	[62.47; 97.53]
Li 2020f	54	78	69.23	[58.99; 79.47]
Liu 2020b	193	291	66.32	[60.89; 71.75]
To 2020	22	23	95.65	[87.32; 100.00]
Wang 2020d	11	11	100.00	[88.69; 100.00]
Xu 2020g	17	21	80.95	[64.16; 97.75]
Zeng 2020	218	338	64.50	[59.40; 69.60]
Zhu 2020b	6	6	100.00	[80.92; 100.00]
**Random effects model**		**788**	**81.24**	**[70.38; 92.10]**

Heterogeneity: $I^2 = 91\%$, $\tau^2 = 0.0207$, $\chi^2_7 = 76.27$ ($p < 0.01$)

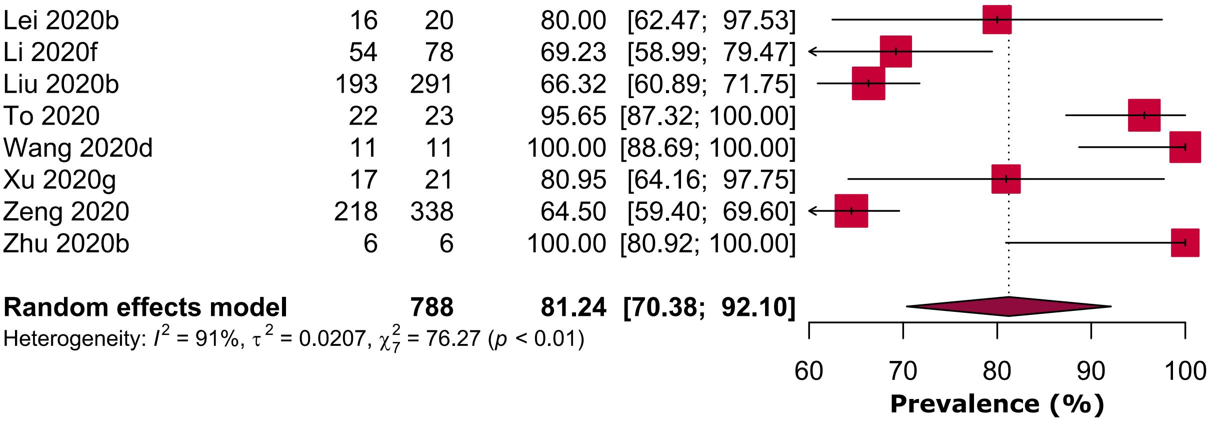

H

Study ID	Cases	Total	Prevalence	95% C.I.
**Guangdong (Paediatric)**				
Feng 2020a	5	15	33.33	[9.48; 57.19]
Tang 2020	11	26	42.31	[23.32; 61.30]
Xu 2020d	7	10	70.00	[41.60; 98.40]
Zhou 2020d	5	9	55.56	[23.09; 88.02]
**Random effects model**		**60**	**47.92**	**[32.95; 62.88]**

Heterogeneity: I^2 = 30%, τ^2 = 0.0070, χ^2_3 = 4.28 (p = 0.23)

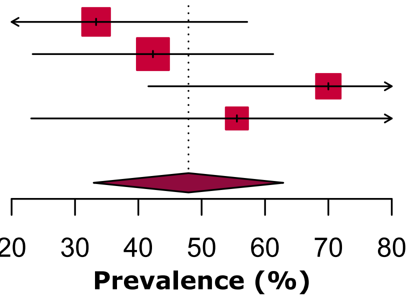

I

Study ID	Cases	Total	Prevalence	95% C.I.
**Hunan (Adult)**				
Feng 2020	105	141	74.47	[67.27; 81.67]
Qiu 2020a	66	104	63.46	[54.21; 72.72]
Zhao 2020b	36	56	64.29	[51.74; 76.84]
**Random effects model**		**301**	**68.26**	**[60.46; 76.07]**

Heterogeneity: I^2 = 51%, τ^2 = 0.0024, χ^2_2 = 4.12 (p = 0.13)

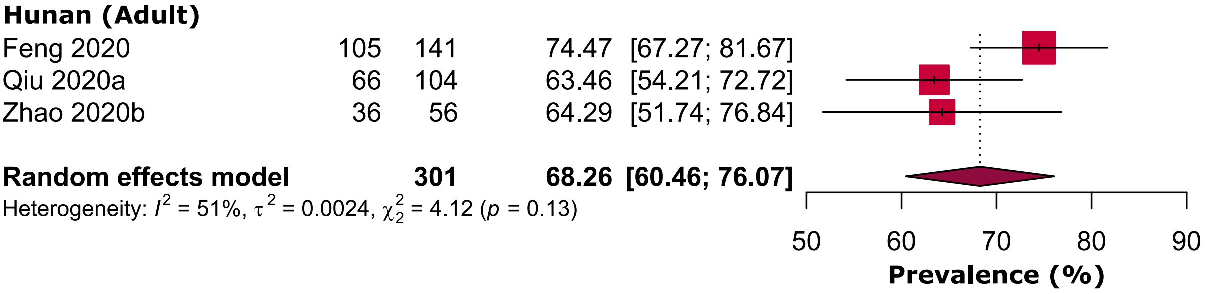

J

Study ID	Cases	Total	Prevalence	95% C.I.
**Beijing (Adult)**				
Chang 2020a	14	16	87.50	[71.30; 100.00]
Li 2020b	34	47	72.34	[59.55; 85.13]
Li 2020e	15	18	83.33	[66.12; 100.00]
Liang 2020	18	21	85.71	[70.75; 100.00]
Yang 2020b	47	55	85.45	[76.14; 94.77]
Yu 2020a	67	76	88.16	[80.89; 95.42]
**Random effects model**		**233**	**84.89**	**[80.34; 89.44]**

Heterogeneity: I^2 = 0%, τ^2 = 0, χ^2_5 = 4.63 (p = 0.46)

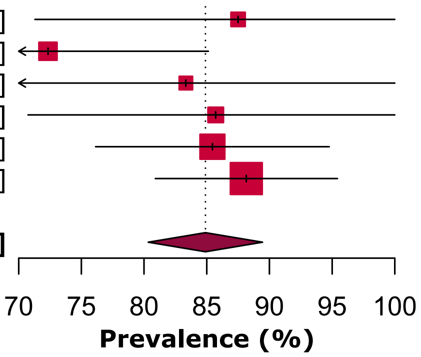

K

Study ID	Cases	Total	Prevalence	95% C.I.
**Anhui (Adult)**				
Liu 2020i	68	73	93.15	[87.36; 98.95]
Zhao 2020	15	19	78.95	[60.62; 97.28]
Zhao 2020g	71	80	88.75	[81.83; 95.67]
Zhu 2020	27	32	84.38	[71.79; 96.96]
**Random effects model**		**204**	**89.66**	**[85.09; 94.24]**

Heterogeneity: I^2 = 12%, τ^2 = 0.0003, χ^2_3 = 3.43 (p = 0.33)

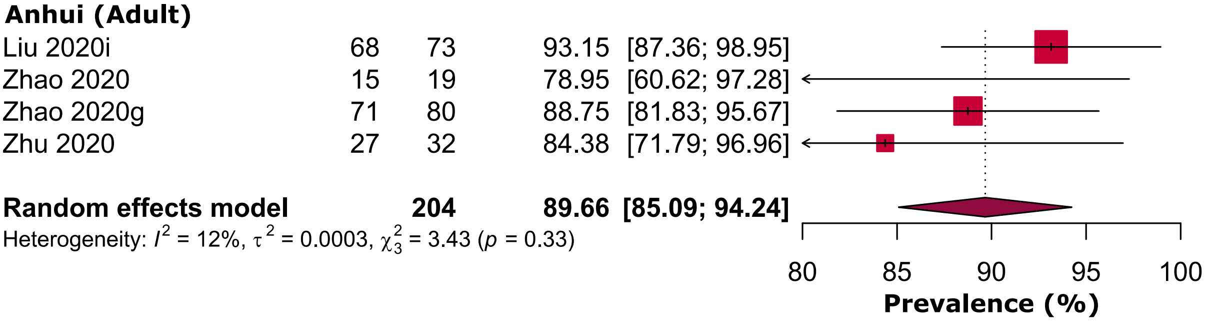

L

Study ID	Cases	Total	Prevalence	95% C.I.
**Hainan (Adult)**				
Liu 2020	41	51	80.39	[69.50; 91.29]
Liu 2020p	44	56	78.57	[67.82; 89.32]
Wu 2020b	72	91	79.12	[70.77; 87.47]
**Random effects model**		**198**	**79.31**	**[73.67; 84.95]**

Heterogeneity: $I^2 = 0\%$, $\tau^2 = 0$, $\chi^2_2 = 0.06$ ($p = 0.97$)

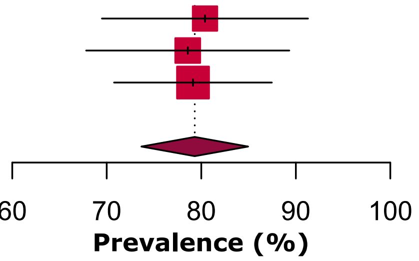

M

Study ID	Cases	Total	Prevalence	95% C.I.
**Fujian (Adult)**				
Wang 2020h	126	165	76.36	[69.88; 82.85]

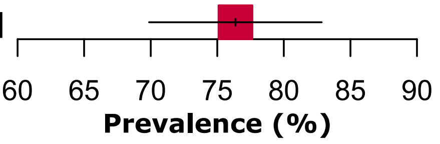

N

Study ID	Cases	Total	Prevalence	95% C.I.
**Hebei (Adult)**				
Chen 2020i	36	37	97.30	[92.07; 100.00]
Li 2020e	15	18	83.33	[66.12; 100.00]
Liang 2020	18	21	85.71	[70.75; 100.00]
Yang 2020b	47	55	85.45	[76.14; 94.77]
**Random effects model**		**131**	**89.73**	**[81.58; 97.88]**

Heterogeneity: $I^2 = 59\%$, $\tau^2 = 0.0038$, $\chi^2_3 = 7.24$ ($p = 0.06$)

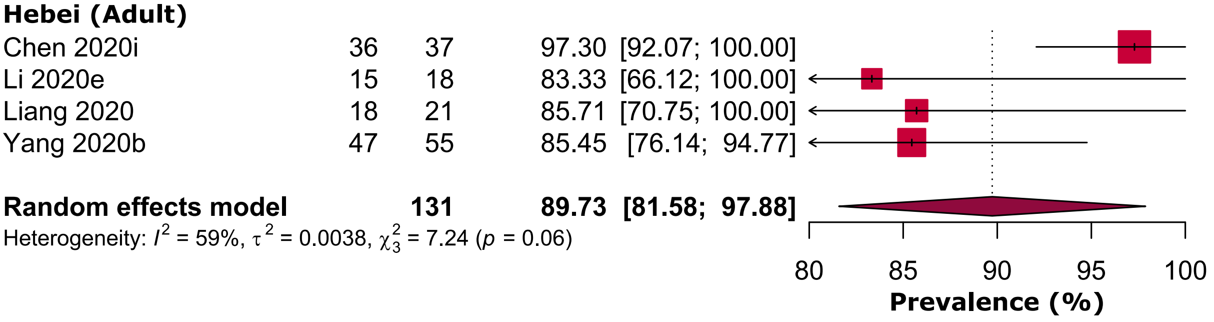

O

Study ID	Cases	Total	Prevalence	95% C.I.
**Sichuan (Adult)**				
Diao 2020	4	6	66.67	[28.95; 100.00]
Fu 2020	45	52	86.54	[77.26; 95.82]
Li 2020	12	17	70.59	[48.93; 92.25]
Xie 2020	8	9	88.89	[68.36; 100.00]
**Random effects model**		**84**	**84.02**	**[76.31; 91.73]**

Heterogeneity: $I^2 = 0\%$, $\tau^2 = 0$, $\chi^2_3 = 2.79$ ($p = 0.43$)

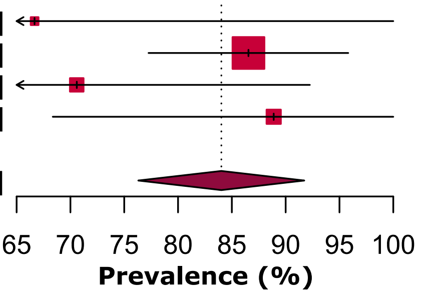

P

Study ID	Cases	Total	Prevalence	95% C.I.
**Shandong (Adult)**				
Tang 2020a	72	73	98.63	[95.96; 100]

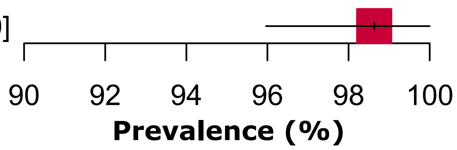

Q

Study ID	Cases	Total	Prevalence	95% C.I.
**Shaanxi (Adult)**				
Bian 2020	27	28	96.43	[89.55; 100]
Zhang 2020c	11	13	84.62	[65.00; 100]
**Random effects model**		**41**	**94.24**	**[85.24; 100]**

Heterogeneity: I^2 = 19%, τ^2 = 0.0014, χ^2_1 = 1.24 (p = 0.27)

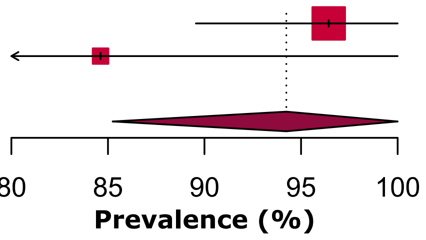

S2 Fig. Prevalence of fever in adult COVID-19 patients from (A) Hubei, (B) Hubei (Paediatric), (C) Zhejiang, (D) Shanghai, (E) Jiangsu, (F) Chongqing, (G) Guangdong, (H) Guangdong (Paediatric), (I) Hunan, (J) Beijing, (K) Anhui, (L) Hainan, (M) Fujian, (N) Hebei, (O) Sichuan, (P) Shandong, and (Q) Shaanxi.
