# Supplementary material for: Prevalence and characteristics of fever in adult and paediatric patients with coronavirus disease 2019 (COVID-19): A systematic review and meta-analysis of 17515 patients
Source: PLoS One. 2021 Apr 6;16(4):e0249788. doi: 10.1371/journal.pone.0249788 (PMC8023501; doi:10.1371/journal.pone.0249788)
Supplement: S3 Fig — (PDF) [file pone.0249788.s004.pdf]

**A**

| Study ID | Cases | Total | Prevalence | 95% C.I. |
|---|---|---|---|---|
| **Severe or critical (Adult)** | | | | |
| Bhatraju 2020 | 12 | 24 | 50.00 | [30.00; 70.00] |
| Cao 2020 | 182 | 199 | 91.46 | [87.57; 95.34] |
| Chen 2020a | 10 | 10 | 100.00 | [87.69; 100.00] |
| Chen 2020i | 36 | 37 | 97.30 | [92.07; 100.00] |
| Chu 2020 | 27 | 43 | 62.79 | [48.34; 77.24] |
| Du 2020 | 99 | 100 | 99.00 | [97.05; 100.00] |
| Feng 2020 | 13 | 15 | 86.67 | [69.46; 100.00] |
| Hu 2020a | 22 | 28 | 78.57 | [63.37; 93.77] |
| Hu 2020b | 151 | 172 | 87.79 | [82.90; 92.68] |
| Huang 2020b | 21 | 25 | 84.00 | [69.63; 98.37] |
| Li 2020b | 34 | 47 | 72.34 | [59.55; 85.13] |
| Li 2020c | 22 | 25 | 88.00 | [75.26; 100.00] |
| Liu 2020a | 6 | 7 | 85.71 | [59.79; 100.00] |
| Liu 2020f | 13 | 13 | 100.00 | [90.28; 100.00] |
| Liu 2020i | 23 | 24 | 95.83 | [87.84; 100.00] |
| Liu 2020o | 45 | 53 | 84.91 | [75.27; 94.54] |
| Lu 2020a | 20 | 22 | 90.91 | [78.90; 100.00] |
| Qi 2020 | 39 | 50 | 78.00 | [66.52; 89.48] |
| Qin 2020 | 271 | 286 | 94.76 | [92.17; 97.34] |
| To 2020 | 10 | 10 | 100.00 | [87.69; 100.00] |
| Wan 2020 | 34 | 40 | 85.00 | [73.93; 96.07] |
| Wang 2020 | 107 | 114 | 93.86 | [89.45; 98.27] |
| Wang 2020d | 11 | 11 | 100.00 | [88.69; 100.00] |
| Xu 2020e | 25 | 25 | 100.00 | [94.72; 100.00] |
| Xu 2020g | 7 | 7 | 100.00 | [83.23; 100.00] |
| Yang 2020a | 51 | 52 | 98.08 | [94.34; 100.00] |
| Zhang 2020a | 29 | 35 | 82.86 | [70.37; 95.34] |
| Zhang 2020b | 51 | 53 | 96.23 | [91.10; 100.00] |
| Zhang 2020f | 55 | 55 | 100.00 | [97.54; 100.00] |
| Zhang 2020l | 31 | 32 | 96.88 | [90.85; 100.00] |
| Zheng 2020 | 19 | 21 | 90.48 | [77.92; 100.00] |
| Zhu 2020a | 34 | 43 | 79.07 | [66.91; 91.23] |
| **Random effects model** | | . | **91.69** | **[89.18; 94.20]** |

Heterogeneity: $I^2 = 78\%$, $\tau^2 = 0.0029$, $\chi^2_{31} = 138.96$ ($p < 0.01$)

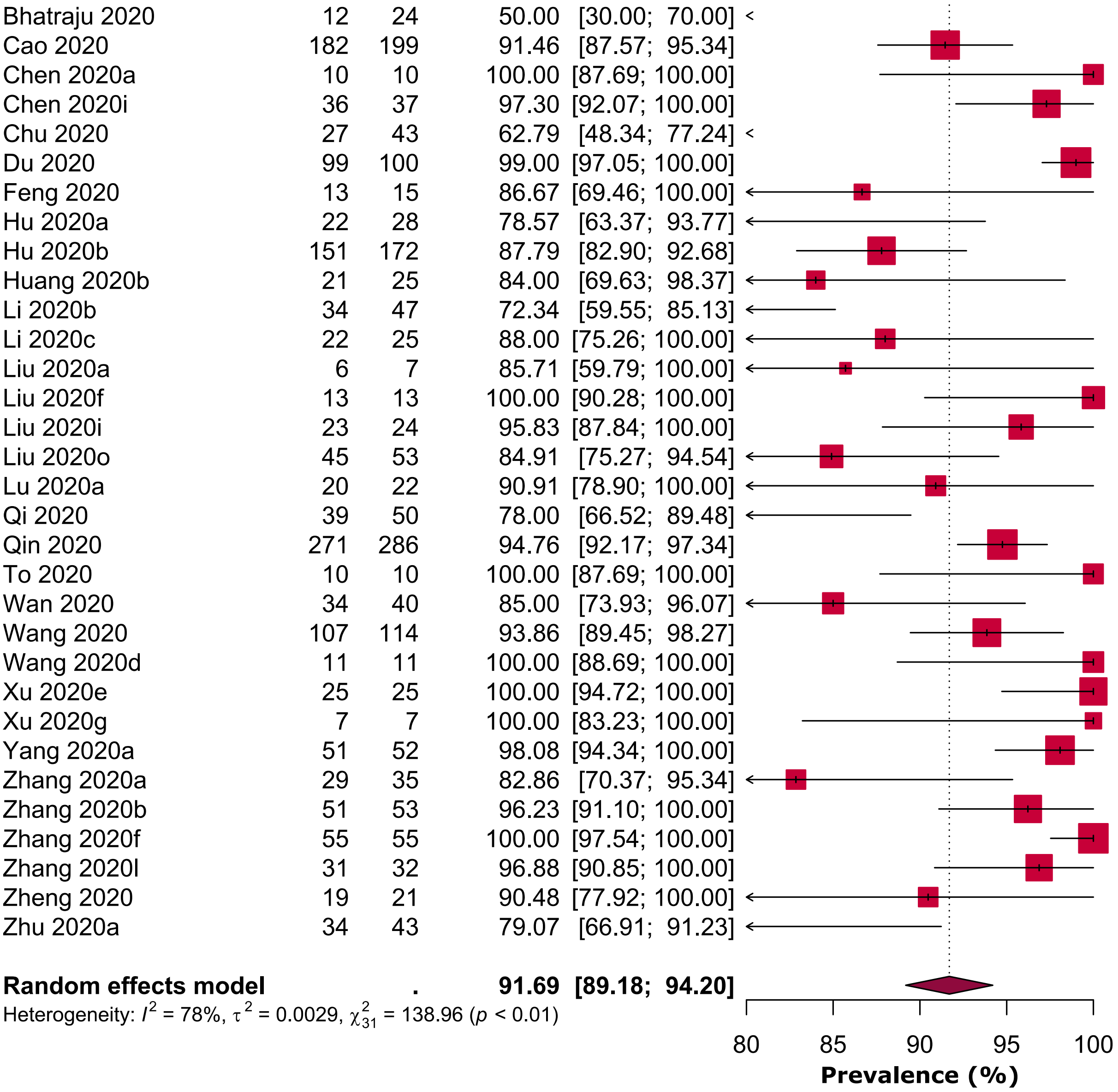

**B**

| Study ID | Cases | Total | Prevalence | 95% C.I. |
|---|---|---|---|---|
| **Non-severe (Adult)** | | | | |
| Chen 2020a | 10 | 10 | 100.00 | [87.69; 100.00] |
| Chu 2020 | 9 | 11 | 81.82 | [59.03; 100.00] |
| Du 2020 | 78 | 79 | 98.73 | [96.27; 100.00] |
| Feng 2020 | 92 | 126 | 73.02 | [65.27; 80.77] |
| Hu 2020a | 87 | 136 | 63.97 | [55.90; 72.04] |
| Hu 2020b | 130 | 151 | 86.09 | [80.57; 91.61] |
| Huang 2020b | 150 | 196 | 76.53 | [70.60; 82.46] |
| Li 2020c | 50 | 58 | 86.21 | [77.33; 95.08] |
| Liu 2020a | 37 | 44 | 84.09 | [73.28; 94.90] |
| Liu 2020f | 23 | 27 | 85.19 | [71.79; 98.58] |
| Liu 2020i | 45 | 49 | 91.84 | [84.17; 99.50] |
| Liu 2020o | 364 | 519 | 70.13 | [66.20; 74.07] |
| Lu 2020a | 200 | 243 | 82.30 | [77.51; 87.10] |
| Qi 2020 | 186 | 217 | 85.71 | [81.06; 90.37] |
| Qin 2020 | 152 | 166 | 91.57 | [87.34; 95.79] |
| To 2020 | 12 | 13 | 92.31 | [77.82; 100.00] |
| Wan 2020 | 86 | 95 | 90.53 | [84.64; 96.42] |
| Wei 2020 | 61 | 100 | 61.00 | [51.44; 70.56] |
| Xu 2020e | 43 | 44 | 97.73 | [93.32; 100.00] |
| Xu 2020g | 10 | 14 | 71.43 | [47.76; 95.09] |
| Zhang 2020a | 35 | 46 | 76.09 | [63.76; 88.41] |
| Zhang 2020b | 59 | 67 | 88.06 | [80.30; 95.82] |
| Zhang 2020f | 145 | 166 | 87.35 | [82.29; 92.41] |
| Zhang 2020l | 56 | 63 | 88.89 | [81.13; 96.65] |
| Zheng 2020 | 26 | 34 | 76.47 | [62.21; 90.73] |
| Zhu 2020a | 59 | 71 | 83.10 | [74.38; 91.82] |
| **Random effects model** | | **2745** | **83.85** | **[79.50; 88.21]** |

Heterogeneity: $I^2$ = 91%, $\tau^2$ = 0.0106, $\chi^2_{25}$ = 288.73 ($p$ < 0.01)

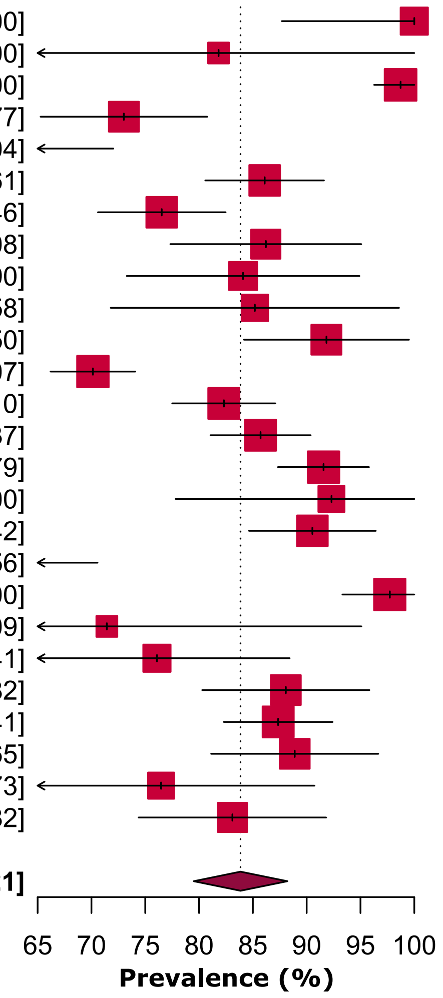

**C**

| Study ID | Cases | Total | Prevalence | 95% C.I. |
|---|---|---|---|---|
| **Survived (Adult)** | | | | |
| Chen 2020l | 145 | 161 | 90.06 | [85.44; 94.68] |
| Deng 2020 | 94 | 116 | 81.03 | [73.90; 88.17] |
| Ding 2020 | 5 | 5 | 100.00 | [77.88; 100.00] |
| Fu 2020 | 45 | 52 | 86.54 | [77.26; 95.82] |
| Han 2020a | 14 | 17 | 82.35 | [64.23; 100.00] |
| Li 2020a | 60 | 96 | 62.50 | [52.82; 72.18] |
| Li 2020f | 54 | 78 | 69.23 | [58.99; 79.47] |
| Liu 2020a | 43 | 51 | 84.31 | [74.33; 94.29] |
| Lu 2020 | 69 | 92 | 75.00 | [66.15; 83.85] |
| Luo 2020 | 232 | 303 | 76.57 | [71.80; 81.34] |
| Pan 2020 | 18 | 21 | 85.71 | [70.75; 100.00] |
| Wang 2020i | 255 | 274 | 93.07 | [90.06; 96.07] |
| Yang 2020a | 20 | 20 | 100.00 | [93.48; 100.00] |
| Yu 2020 | 6 | 7 | 85.71 | [59.79; 100.00] |
| Yuan 2020a | 15 | 17 | 88.24 | [72.92; 100.00] |
| Zhang 2020k | 219 | 273 | 80.22 | [75.49; 84.95] |
| Zhou 2020 | 129 | 137 | 94.16 | [90.23; 98.09] |
| **Random effects model** | | **1720** | **84.17** | **[79.41; 88.94]** |

Heterogeneity: $I^2 = 87\%$, $\tau^2 = 0.0073$, $\chi^2_{16} = 119.38$ ($p < 0.01$)

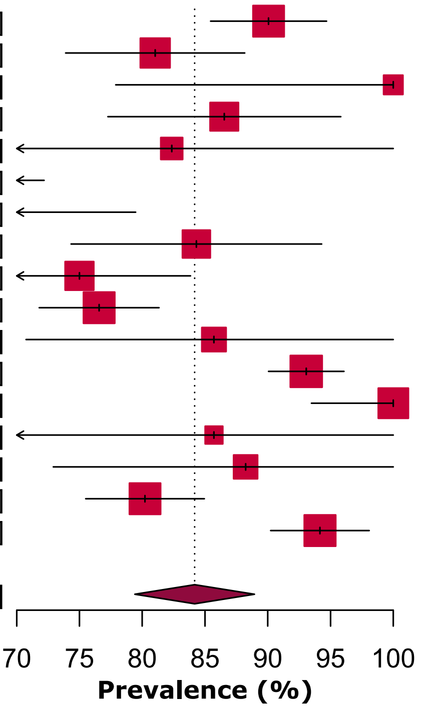


**D**

| Study ID | Cases | Total | Prevalence | 95% C.I. |
|---|---|---|---|---|
| **Non-survived (Adult)** | | | | |
| Chen 2020l | 104 | 113 | 92.04 | [87.04; 97.03] |
| Deng 2020 | 95 | 109 | 87.16 | [80.87; 93.44] |
| Fan 2020 | 91 | 101 | 90.10 | [84.27; 95.92] |
| Huang 2020c | 34 | 36 | 94.44 | [86.96; 100.00] |
| Li 2020a | 59 | 65 | 90.77 | [83.73; 97.81] |
| Lu 2020 | 29 | 31 | 93.55 | [84.90; 100.00] |
| Luo 2020 | 87 | 100 | 87.00 | [80.41; 93.59] |
| Shi 2020a | 82 | 101 | 81.19 | [73.57; 88.81] |
| Wang 2020i | 56 | 65 | 86.15 | [77.76; 94.55] |
| Yang 2020a | 31 | 32 | 96.88 | [90.85; 100.00] |
| Yuan 2020a | 6 | 10 | 60.00 | [29.64; 90.36] |
| Zhang 2020k | 40 | 46 | 86.96 | [77.22; 96.69] |
| Zhou 2020 | 51 | 54 | 94.44 | [88.33; 100.00] |
| **Random effects model** | | **863** | **90.13** | **[87.47; 92.79]** |

Heterogeneity: $I^2 = 43\%$, $\tau^2 = 0.0010$, $\chi^2_{12} = 21.15$ ($p = 0.05$)

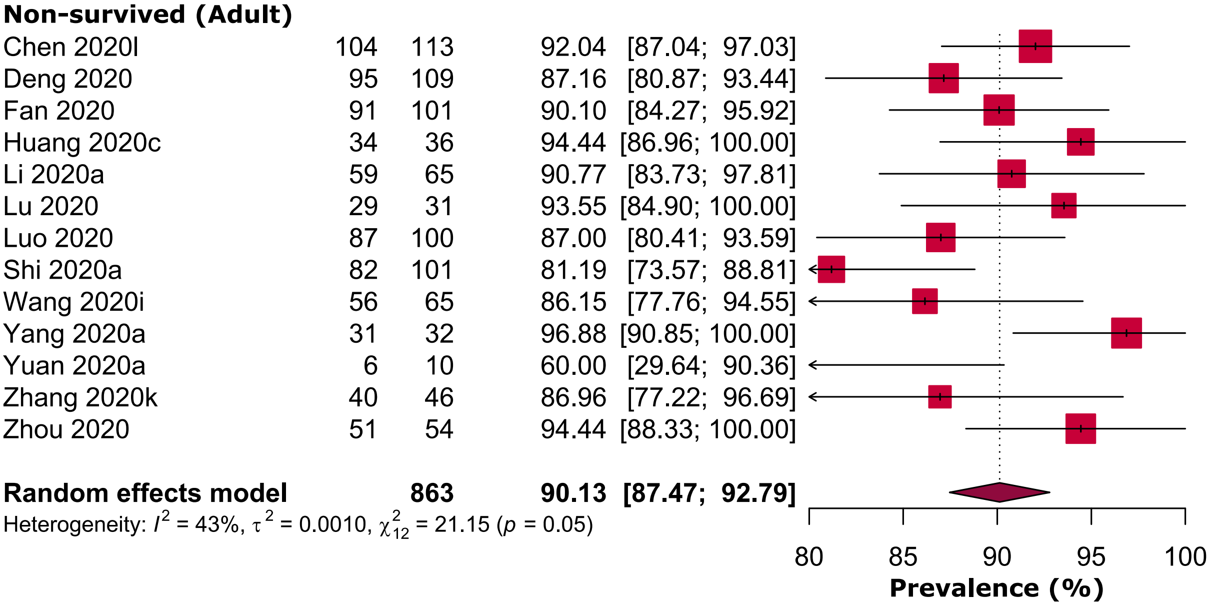

## E

| Study ID | Cases | Total | Prevalence | 95% C.I. |
|---|---|---|---|---|
| **ICU (Adult)** | | | | |
| Cao 2020a | 18 | 19 | 94.74 | [84.70; 100] |
| Huang 2020 | 13 | 13 | 100.00 | [90.28; 100] |
| Qin 2020a | 35 | 36 | 97.22 | [91.85; 100] |
| Wang 2020a | 36 | 36 | 100.00 | [96.28; 100] |
| **Random effects model** | | **104** | **98.83** | **[96.03; 100]** |

Heterogeneity: $I^2 = 0\%$, $\tau^2 = 0$, $\chi^2_3 = 1.42$ ($p = 0.70$)

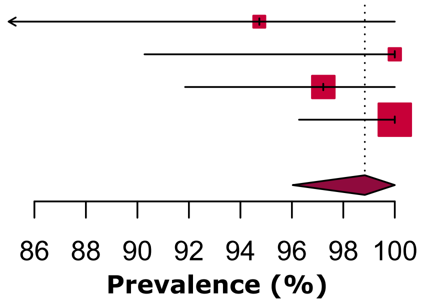


## F

| Study ID | Cases | Total | Prevalence | 95% C.I. |
|---|---|---|---|---|
| **Non-ICU (Adult)** | | | | |
| Cao 2020a | 154 | 179 | 86.03 | [80.96; 91.11] |
| Huang 2020 | 27 | 28 | 96.43 | [89.55; 100.00] |
| Qin 2020a | 51 | 53 | 96.23 | [91.10; 100.00] |
| Wang 2020a | 100 | 102 | 98.04 | [95.35; 100.00] |
| **Random effects model** | | **362** | **94.27** | **[88.70; 99.83]** |

Heterogeneity: $I^2 = 82\%$, $\tau^2 = 0.0026$, $\chi^2_3 = 16.90$ ($p < 0.01$)

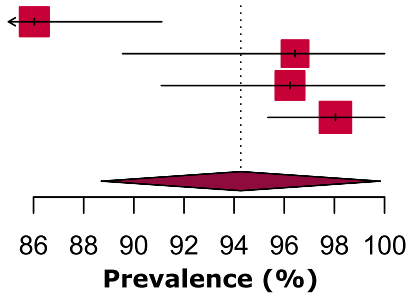


## G

| Study ID | Cases | Total | Prevalence | 95% C.I. |
|---|---|---|---|---|
| **Pregnant women or new mothers** | | | | |
| Chen 2020c | 7 | 9 | 77.78 | [50.62; 100.00] |
| Chen 2020g | 4 | 17 | 23.53 | [ 3.37; 43.69] |
| Chen 2020m | 0 | 5 | 0.00 | [ 0.00; 22.12] |
| Cheng 2020b | 15 | 31 | 48.39 | [30.80; 65.98] |
| Liu 2020c | 13 | 15 | 86.67 | [69.46; 100.00] |
| Liu 2020e | 16 | 41 | 39.02 | [24.09; 53.96] |
| Liu 2020j | 10 | 13 | 76.92 | [54.02; 99.83] |
| Nie 2020 | 21 | 33 | 63.64 | [47.22; 80.05] |
| Yu 2020 | 6 | 7 | 85.71 | [59.79; 100.00] |
| Zhang 2020h | 31 | 89 | 34.83 | [24.93; 44.73] |
| Zhu 2020c | 9 | 10 | 90.00 | [71.41; 100.00] |
| **Random effects model** | | **270** | **56.45** | **[40.15; 72.75]** |

Heterogeneity: $I^2 = 89\%$, $\tau^2 = 0.0660$, $\chi^2_{10} = 91.56$ ($p < 0.01$)

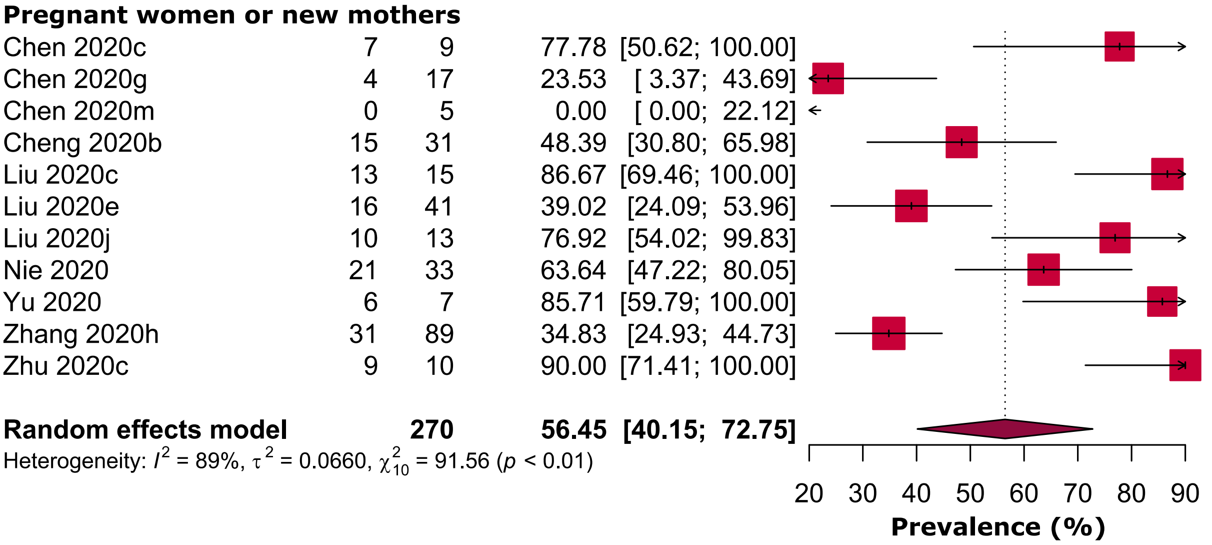


**S3 Fig. Prevalence of fever in (A) severe or critical, (B) non-severe, (C) survived (recovered or discharged), (D) non-survived, (E) ICU, (F) non-ICU, and (G) pregnant women or new mothers with COVID-19.**
