# Supplementary material for: Prevalence and characteristics of fever in adult and paediatric patients with coronavirus disease 2019 (COVID-19): A systematic review and meta-analysis of 17515 patients
Source: PLoS One. 2021 Apr 6;16(4):e0249788. doi: 10.1371/journal.pone.0249788 (PMC8023501; doi:10.1371/journal.pone.0249788)
Supplement: S4 Fig — (PDF) [file pone.0249788.s005.pdf]

A

Study ID	Cases	Total	Prevalence	95% C.I.
**Severe (Adult)**				
Chen 2020a	0	9	0.00	[0.00; 13.51]
Du 2020	12	44	27.27	[14.11; 40.43]
Hu 2020b	62	102	60.78	[51.31; 70.26]
Liu 2020f	2	13	15.38	[0.00; 35.00]
Wan 2020	27	30	90.00	[79.26; 100.00]
Zhang 2020f	6	55	10.91	[2.67; 19.15]
Zhang 2020l	2	31	6.45	[0.00; 15.10]
**Random effects model**		**284**	**30.27**	**[4.74; 55.79]**

Heterogeneity: $I^2 = 97\%$, $\tau^2 = 0.1147$, $\chi^2_6 = 230.75$ ($p < 0.01$)

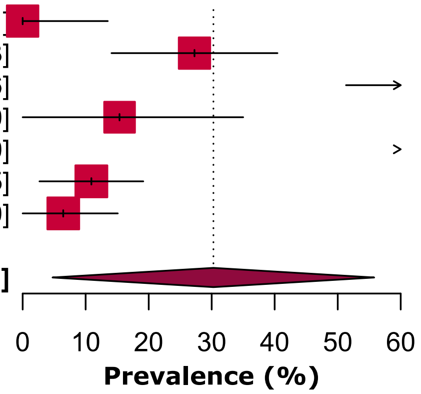

B

Study ID	Cases	Total	Prevalence	95% C.I.
**Severe (Adult)**				
Chen 2020a	5	9	55.56	[23.09; 88.02]
Du 2020	25	44	56.82	[42.18; 71.45]
Hu 2020b	33	102	32.35	[23.27; 41.43]
Liu 2020f	8	13	61.54	[35.09; 87.98]
Wan 2020	2	30	6.67	[0.00; 15.59]
Zhang 2020f	36	55	65.45	[52.89; 78.02]
Zhang 2020l	10	31	32.26	[15.80; 48.71]
**Random effects model**		**284**	**43.17**	**[24.44; 61.90]**

Heterogeneity: $I^2 = 92\%$, $\tau^2 = 0.0555$, $\chi^2_6 = 75.45$ ($p < 0.01$)

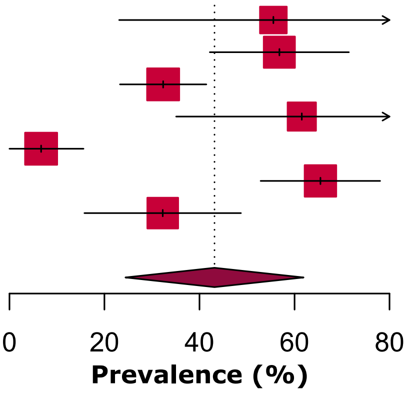

C

Study ID	Cases	Total	Prevalence	95% C.I.
**Severe (Adult)**				
Chen 2020a	4	9	44.44	[11.98; 76.91]
Du 2020	7	44	15.91	[5.10; 26.72]
Hu 2020b	7	102	6.86	[1.96; 11.77]
Liu 2020f	3	13	23.08	[0.17; 45.98]
Wan 2020	1	30	3.33	[0.00; 9.76]
Zhang 2020f	13	55	23.64	[12.41; 34.86]
Zhang 2020l	19	31	61.29	[44.14; 78.44]
**Random effects model**		**284**	**22.39**	**[10.51; 34.28]**

Heterogeneity: $I^2 = 88\%$, $\tau^2 = 0.0198$, $\chi^2_6 = 52.05$ ($p < 0.01$)

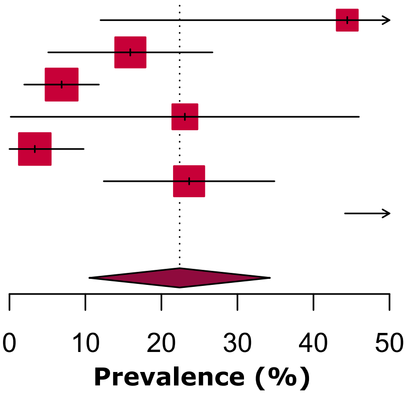

S4 Fig. Prevalence of (A) low-grade (37·3-38·0°C), (B) medium-grade (38·1-39·0°C), and (C) high-grade fever (>39·0°C) in severe or critical COVID-19 adult patients.
