# Supplementary material for: Prevalence and characteristics of fever in adult and paediatric patients with coronavirus disease 2019 (COVID-19): A systematic review and meta-analysis of 17515 patients
Source: PLoS One. 2021 Apr 6;16(4):e0249788. doi: 10.1371/journal.pone.0249788 (PMC8023501; doi:10.1371/journal.pone.0249788)
Supplement: S5 Fig — (PDF) [file pone.0249788.s006.pdf]

A

Study ID	Cases	Total	Prevalence	95% C.I.
**Non-severe (Adult)**				
Chen 2020a	0	10	0.00	[0.00; 12.31]
Du 2020	14	26	53.85	[34.68; 73.01]
Hu 2020b	43	87	49.43	[38.92; 59.93]
Liu 2020f	8	23	34.78	[15.32; 54.25]
Wan 2020	43	84	51.19	[40.50; 61.88]
Zhang 2020f	51	145	35.17	[27.40; 42.94]
Zhang 2020l	17	56	30.36	[18.31; 42.40]
**Random effects model**		**431**	**36.16**	**[22.93; 49.39]**

Heterogeneity: $I^2 = 88\%$, $\tau^2 = 0.0272$, $\chi^2_6 = 51.11$ ($p < 0.01$)

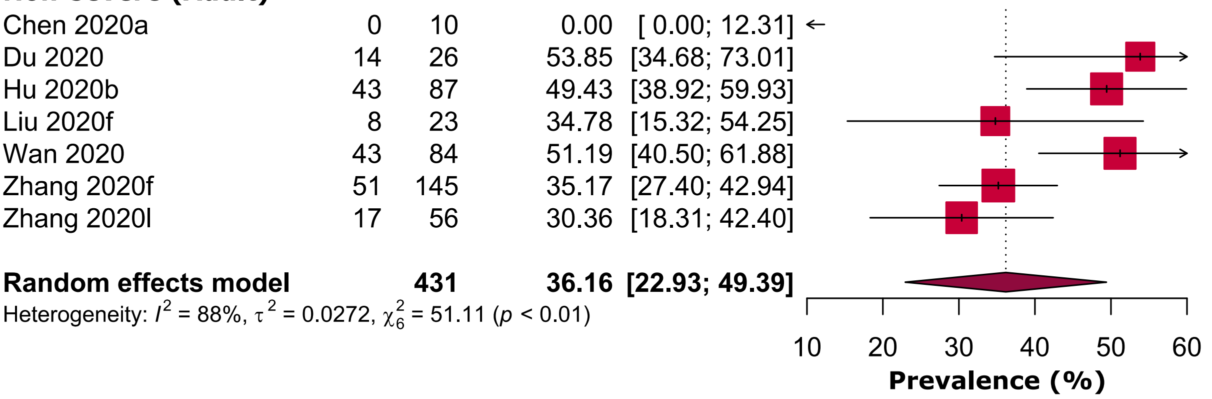

B

Study ID	Cases	Total	Prevalence	95% C.I.
**Non-severe (Adult)**				
Chen 2020a	7	10	70.00	[41.60; 98.40]
Du 2020	11	26	42.31	[23.32; 61.30]
Hu 2020b	34	87	39.08	[28.83; 49.33]
Liu 2020f	9	23	39.13	[19.19; 59.08]
Wan 2020	35	84	41.67	[31.12; 52.21]
Zhang 2020f	68	145	46.90	[38.77; 55.02]
Zhang 2020l	25	56	44.64	[31.62; 57.66]
**Random effects model**		**431**	**43.90**	**[39.24; 48.55]**

Heterogeneity: $I^2 = 0\%$, $\tau^2 = 0$, $\chi^2_6 = 5.05$ ($p = 0.54$)

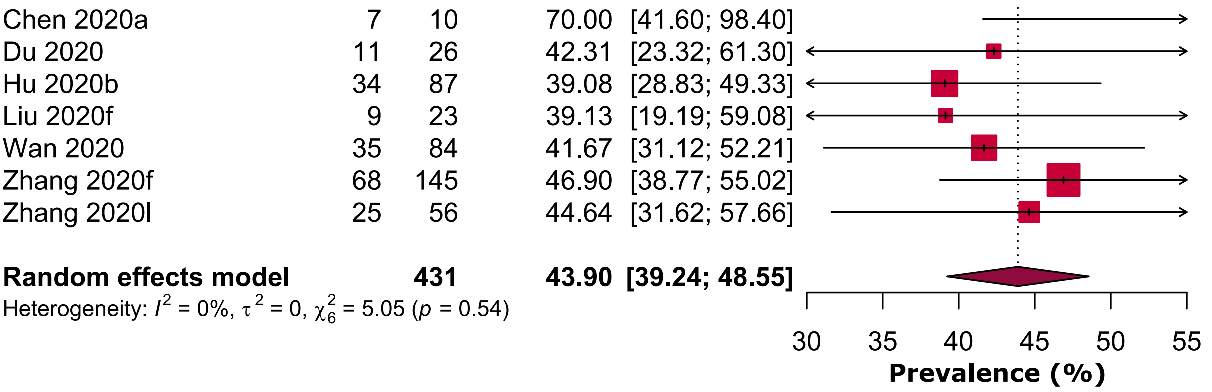

C

Study ID	Cases	Total	Prevalence	95% C.I.
**Non-severe (Adult)**				
Chen 2020a	3	10	30.00	[1.60; 58.40]
Du 2020	1	26	3.85	[0.00; 11.24]
Hu 2020b	10	87	11.49	[4.79; 18.20]
Liu 2020f	6	23	26.09	[8.14; 44.03]
Wan 2020	6	84	7.14	[1.64; 12.65]
Zhang 2020f	26	145	17.93	[11.69; 24.17]
Zhang 2020l	14	56	25.00	[13.66; 36.34]
**Random effects model**		**431**	**14.16**	**[7.99; 20.33]**

Heterogeneity: $I^2 = 70\%$, $\tau^2 = 0.0042$, $\chi^2_6 = 20.11$ ($p < 0.01$)

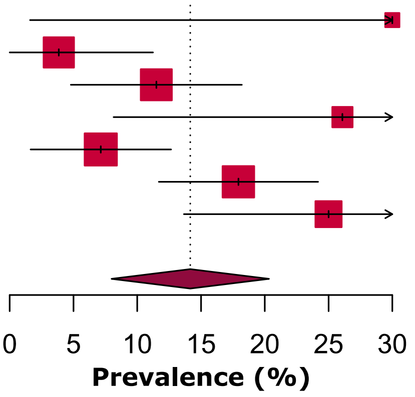

S5 Fig. Prevalence of (A) low-grade (37·3-38·0°C), (B) medium-grade (38·1-39·0°C), and (C) high-grade fever (>39·0°C) in non-severe COVID-19 adult patients.
