# Supplementary material for: Prevalence and characteristics of fever in adult and paediatric patients with coronavirus disease 2019 (COVID-19): A systematic review and meta-analysis of 17515 patients
Source: PLoS One. 2021 Apr 6;16(4):e0249788. doi: 10.1371/journal.pone.0249788 (PMC8023501; doi:10.1371/journal.pone.0249788)
Supplement: S6 Fig — (PDF) [file pone.0249788.s007.pdf]

A

Study ID	Cases	Total	Prevalence	95% C.I.
**Survived (Adult)**				
Li 2020a	25	60	41.67	[29.19; 54.14]
Li 2020f	32	54	59.26	[46.15; 72.36]
Pan 2020	6	18	33.33	[11.56; 55.11]
**Random effects model**		**132**	**46.19**	**[31.54; 60.83]**

Heterogeneity: $I^2 = 64\%$, $\tau^2 = 0.0105$, $\chi^2_2 = 5.56$ ($p = 0.06$)

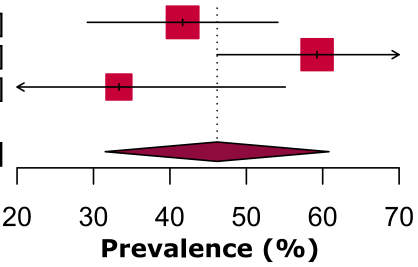

B

Study ID	Cases	Total	Prevalence	95% C.I.
**Survived (Adult)**				
Li 2020a	30	60	50.00	[37.35; 62.65]
Li 2020f	20	54	37.04	[24.16; 49.92]
Pan 2020	7	18	38.89	[16.37; 61.41]
**Random effects model**		**132**	**42.94**	**[34.23; 51.65]**

Heterogeneity: $I^2 = 6\%$, $\tau^2 = 0.0004$, $\chi^2_2 = 2.13$ ($p = 0.35$)

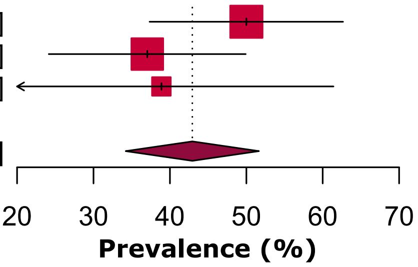

C

Study ID	Cases	Total	Prevalence	95% C.I.
**Survived (Adult)**				
Li 2020a	5	60	8.33	[1.34; 15.33]
Li 2020f	2	54	3.70	[0.00; 8.74]
Pan 2020	5	18	27.78	[7.09; 48.47]
**Random effects model**		**132**	**8.51**	**[0.38; 16.64]**

Heterogeneity: $I^2 = 63\%$, $\tau^2 = 0.0030$, $\chi^2_2 = 5.48$ ($p = 0.06$)

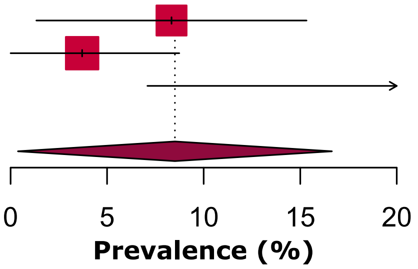

S6 Fig. Prevalence of (A) low-grade (37·3-38·0°C), (B) medium-grade (38·1-39·0°C), and (C) high-grade fever (>39·0°C) in survived (recovered or discharged) adult COVID-19 patients.
