# Supplementary material for: Prevalence and characteristics of fever in adult and paediatric patients with coronavirus disease 2019 (COVID-19): A systematic review and meta-analysis of 17515 patients
Source: PLoS One. 2021 Apr 6;16(4):e0249788. doi: 10.1371/journal.pone.0249788 (PMC8023501; doi:10.1371/journal.pone.0249788)
Supplement: S7 Fig — (PDF) [file pone.0249788.s008.pdf]

**A**

| Study ID | Cases | Total | Prevalence | 95% C.I. |
|---|---|---|---|---|
| **Non-survived (Adult)** | | | | |
| Fan 2020 | 33 | 91 | 36.26 | [26.39; 46.14] |
| Li 2020a | 17 | 59 | 28.81 | [17.26; 40.37] |
| Shi 2020 | 20 | 57 | 35.09 | [22.70; 47.48] |
| **Random effects model** | | **207** | **33.65** | **[27.23; 40.07]** |

Heterogeneity: $I^2 = 0\%$, $\tau^2 = 0$, $\chi^2_2 = 0.99$ ($p = 0.61$)

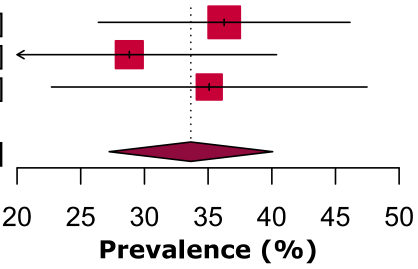


**B**

| Study ID | Cases | Total | Prevalence | 95% C.I. |
|---|---|---|---|---|
| **Non-survived (Adult)** | | | | |
| Fan 2020 | 42 | 91 | 46.15 | [35.91; 56.40] |
| Li 2020a | 34 | 59 | 57.63 | [45.02; 70.24] |
| Shi 2020 | 23 | 57 | 40.35 | [27.61; 53.09] |
| **Random effects model** | | **207** | **47.93** | **[38.60; 57.26]** |

Heterogeneity: $I^2 = 47\%$, $\tau^2 = 0.0032$, $\chi^2_2 = 3.75$ ($p = 0.15$)

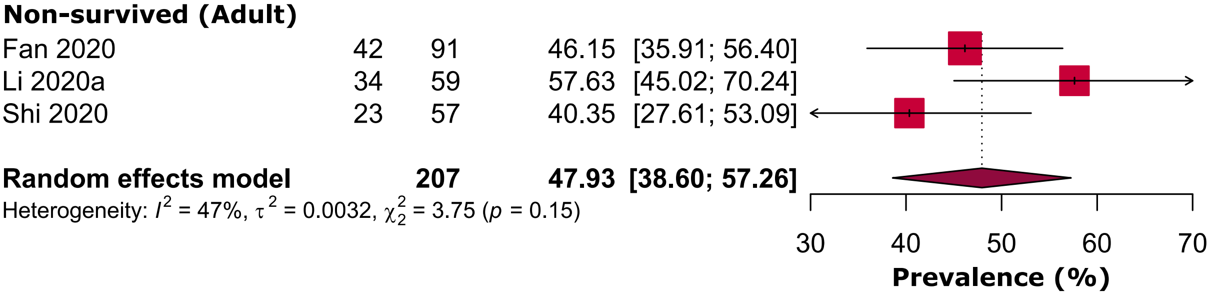


**C**

| Study ID | Cases | Total | Prevalence | 95% C.I. |
|---|---|---|---|---|
| **Non-survived (Adult)** | | | | |
| Fan 2020 | 16 | 91 | 17.58 | [ 9.76; 25.40] |
| Li 2020a | 8 | 59 | 13.56 | [ 4.82; 22.30] |
| Shi 2020 | 14 | 57 | 24.56 | [13.39; 35.74] |
| **Random effects model** | | **207** | **17.76** | **[12.16; 23.35]** |

Heterogeneity: $I^2 = 13\%$, $\tau^2 = 0.0003$, $\chi^2_2 = 2.31$ ($p = 0.31$)

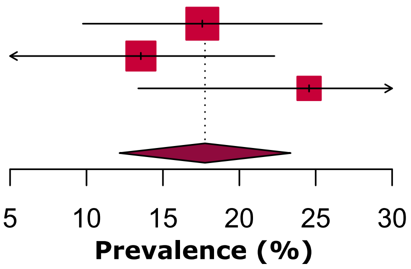


**S7 Fig. Prevalence of (A) low-grade (37·3-38·0°C), (B) medium-grade (38·1-39·0°C), and (C) high-grade fever (>39·0°C) in non-survived adult COVID-19 patients.**
