# Supplementary material for: Prevalence and characteristics of fever in adult and paediatric patients with coronavirus disease 2019 (COVID-19): A systematic review and meta-analysis of 17515 patients
Source: PLoS One. 2021 Apr 6;16(4):e0249788. doi: 10.1371/journal.pone.0249788 (PMC8023501; doi:10.1371/journal.pone.0249788)
Supplement: S9 Fig — (PDF) [file pone.0249788.s010.pdf]

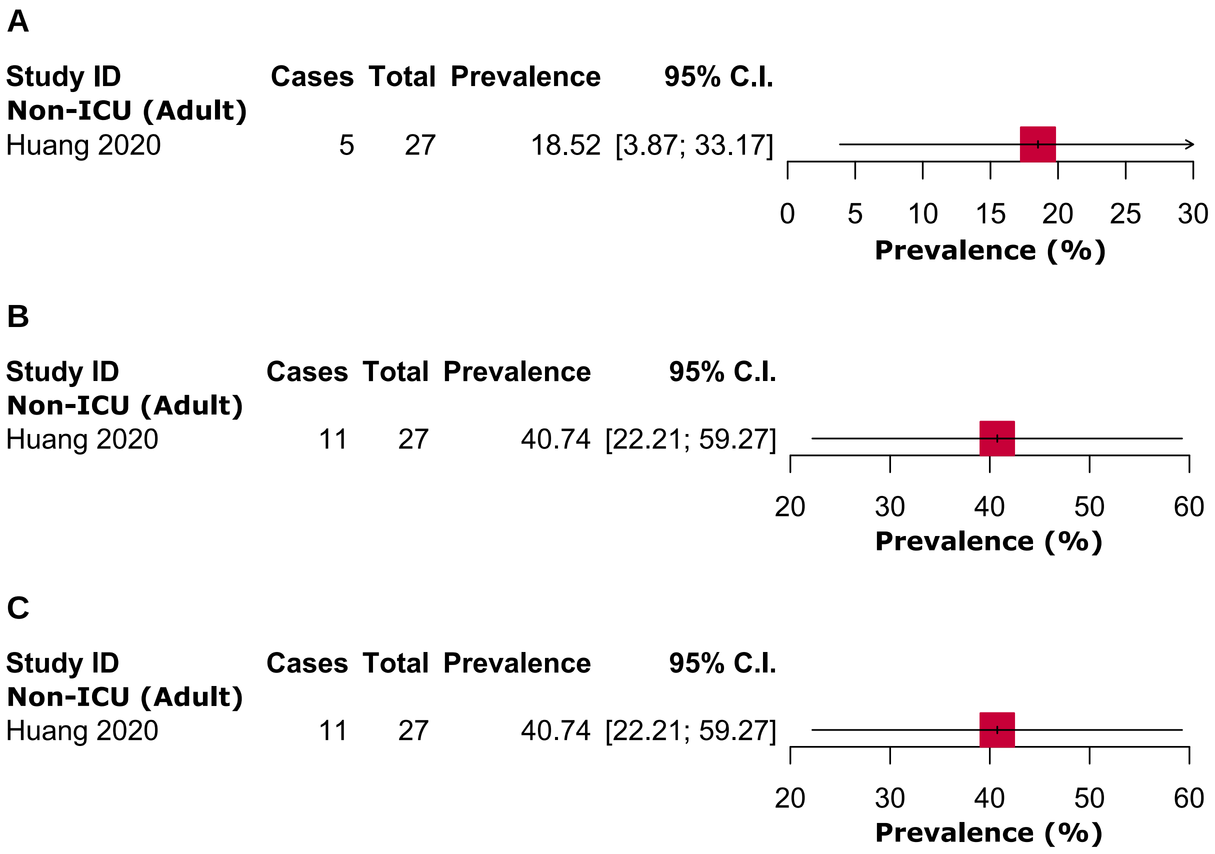

S9 Fig. Prevalence of (A) low-grade (37·3-38·0°C), (B) medium-grade (38·1-39·0°C), and (C) high-grade fever (>39·0°C) in adult COVID-19 non-ICU patients.
