# Supplementary material for: Prevalence and characteristics of fever in adult and paediatric patients with coronavirus disease 2019 (COVID-19): A systematic review and meta-analysis of 17515 patients
Source: PLoS One. 2021 Apr 6;16(4):e0249788. doi: 10.1371/journal.pone.0249788 (PMC8023501; doi:10.1371/journal.pone.0249788)
Supplement: S10 Fig — (PDF) [file pone.0249788.s011.pdf]

A

Study or Subgroup	Medium-grade fever Events	Medium-grade fever Total	Low-grade fever Events	Low-grade fever Total	Weight	Risk Ratio M-H, Random, 95% CI
Wan 2020	2	30	27	30	13.9%	0.07 [0.02, 0.28]
Hu 2020b	33	102	62	102	17.5%	0.53 [0.39, 0.73]
Du 2020	25	44	12	44	17.0%	2.08 [1.21, 3.60]
Liu 2020f	8	13	2	13	13.9%	4.00 [1.04, 15.36]
Zhang 2020l	10	31	2	31	13.5%	5.00 [1.19, 20.98]
Zhang 2020f	36	55	6	55	16.2%	6.00 [2.75, 13.08]
Chen 2020a	5	9	0	9	8.1%	11.00 [0.70, 173.66]
**Total (95% CI)**		**284**		**284**	**100.0%**	**1.73 [0.59, 5.03]**
Total events	119		111			

Heterogeneity: Tau² = 1.67; Chi² = 68.73, df = 6 (P < 0.00001); I² = 91%
Test for overall effect: Z = 1.01 (P = 0.31)

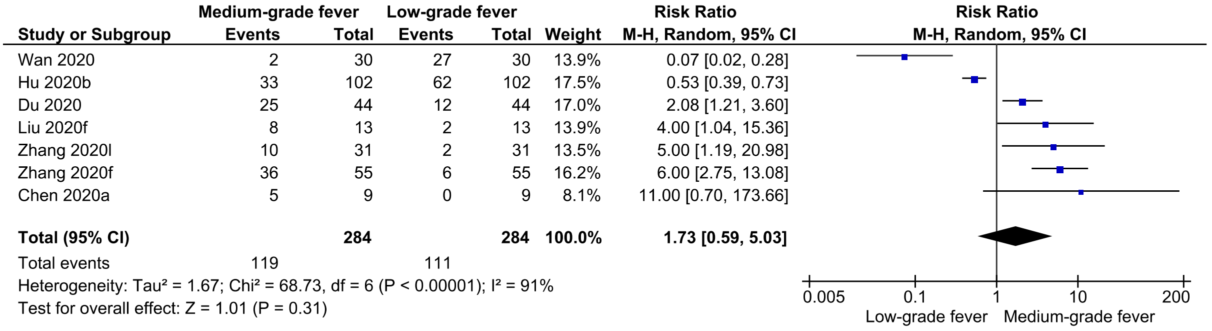

B

Study or Subgroup	Low-grade fever Events	Low-grade fever Total	High-grade fever Events	High-grade fever Total	Weight	Risk Ratio M-H, Random, 95% CI
Zhang 2020l	2	31	19	31	14.7%	0.11 [0.03, 0.41]
Chen 2020a	0	9	4	9	10.1%	0.11 [0.01, 1.80]
Zhang 2020f	6	55	13	55	16.0%	0.46 [0.19, 1.13]
Liu 2020f	2	13	3	13	13.9%	0.67 [0.13, 3.35]
Du 2020	12	44	7	44	16.1%	1.71 [0.75, 3.94]
Hu 2020b	62	102	7	102	16.3%	8.86 [4.26, 18.41]
Wan 2020	27	30	1	30	12.9%	27.00 [3.92, 186.15]
**Total (95% CI)**		**284**		**284**	**100.0%**	**1.14 [0.29, 4.57]**
Total events	111		54			

Heterogeneity: Tau² = 2.91; Chi² = 57.22, df = 6 (P < 0.00001); I² = 90%
Test for overall effect: Z = 0.19 (P = 0.85)

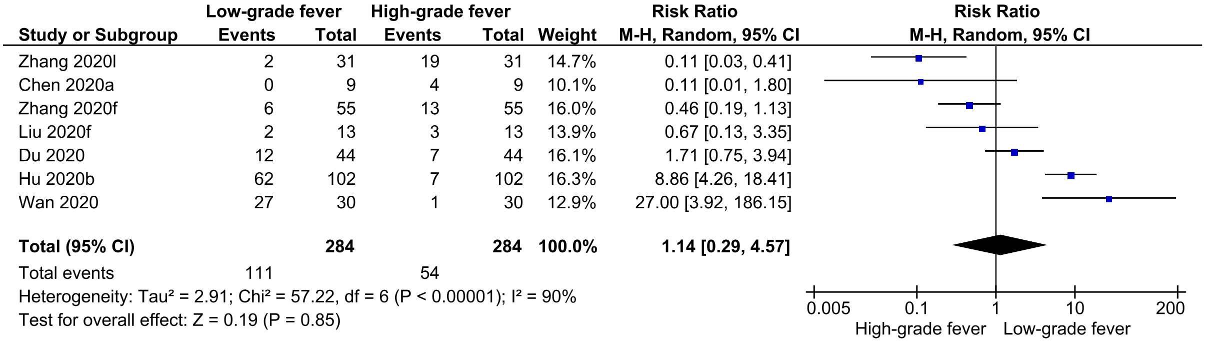

C

Study or Subgroup	Medium-grade fever Events	Medium-grade fever Total	High-grade fever Events	High-grade fever Total	Weight	Risk Ratio M-H, Random, 95% CI
Zhang 2020l	10	31	19	31	17.1%	0.53 [0.29, 0.94]
Chen 2020a	5	9	4	9	14.4%	1.25 [0.49, 3.19]
Wan 2020	2	30	1	30	6.1%	2.00 [0.19, 20.90]
Liu 2020f	8	13	3	13	13.2%	2.67 [0.90, 7.86]
Zhang 2020f	36	55	13	55	17.6%	2.77 [1.66, 4.62]
Du 2020	25	44	7	44	16.0%	3.57 [1.73, 7.39]
Hu 2020b	33	102	7	102	15.7%	4.71 [2.19, 10.16]
**Total (95% CI)**		**284**		**284**	**100.0%**	**2.05 [1.02, 4.12]**
Total events	119		54			

Heterogeneity: Tau² = 0.65; Chi² = 31.04, df = 6 (P < 0.0001); I² = 81%
Test for overall effect: Z = 2.03 (P = 0.04)

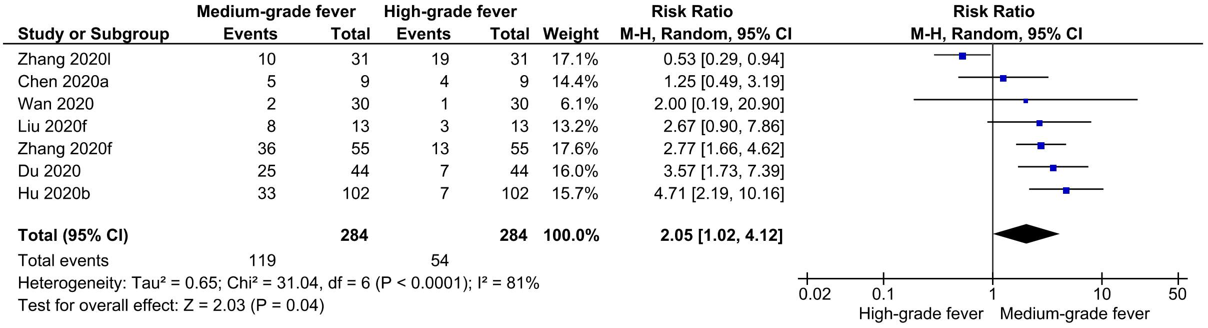

S10 Fig. Risks of (A) low-grade fever (37·3-38·0°C) vs medium-grade fever (38·1-39·0°C), (B) high-grade fever (>39·0°C) vs low-grade fever (37·3-38·0°C), (C) and high-grade fever (>39·0°C) vs medium-grade fever (38·1-39·0°C) in adult severe or critical COVID-19 patients.
