# Supplementary material for: Prevalence and characteristics of fever in adult and paediatric patients with coronavirus disease 2019 (COVID-19): A systematic review and meta-analysis of 17515 patients
Source: PLoS One. 2021 Apr 6;16(4):e0249788. doi: 10.1371/journal.pone.0249788 (PMC8023501; doi:10.1371/journal.pone.0249788)
Supplement: S11 Fig — (PDF) [file pone.0249788.s012.pdf]

A

Study or Subgroup	Medium-grade fever Events	Medium-grade fever Total	Low-grade fever Events	Low-grade fever Total	Weight	Risk Ratio M-H, Random, 95% CI
Du 2020	11	26	14	26	12.8%	0.79 [0.44, 1.39]
Hu 2020b	34	87	43	87	19.9%	0.79 [0.56, 1.11]
Wan 2020	35	84	43	84	20.3%	0.81 [0.59, 1.13]
Liu 2020f	9	23	8	23	9.1%	1.13 [0.53, 2.40]
Zhang 2020f	68	145	51	145	21.9%	1.33 [1.01, 1.77]
Zhang 2020l	25	56	17	56	15.0%	1.47 [0.90, 2.41]
Chen 2020a	7	10	0	10	1.0%	15.00 [0.97, 231.84]
**Total (95% CI)**		**431**		**431**	**100.0%**	**1.04 [0.79, 1.37]**
Total events	189		176			

Heterogeneity: Tau² = 0.07; Chi² = 14.46, df = 6 (P = 0.02); I² = 59%
Test for overall effect: Z = 0.27 (P = 0.78)

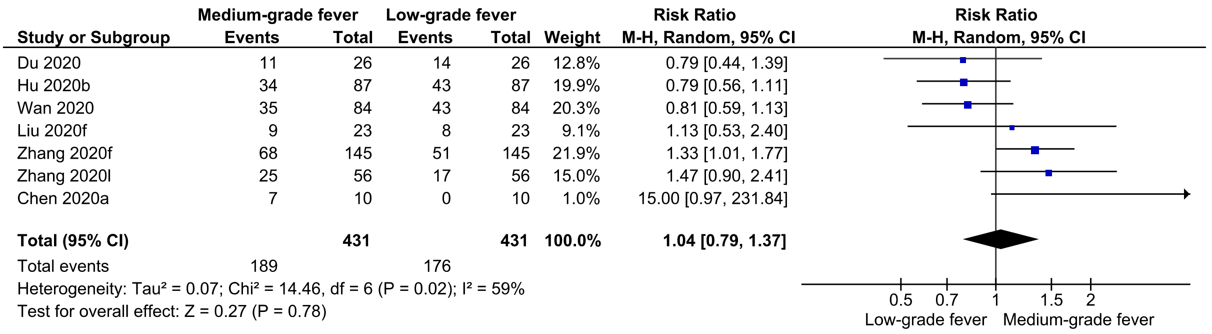

B

Study or Subgroup	Low-grade fever Events	Low-grade fever Total	High-grade fever Events	High-grade fever Total	Weight	Risk Ratio M-H, Random, 95% CI
Chen 2020a	0	10	3	10	4.1%	0.14 [0.01, 2.45]
Zhang 2020l	17	56	14	56	18.4%	1.21 [0.66, 2.22]
Liu 2020f	8	23	6	23	15.5%	1.33 [0.55, 3.24]
Zhang 2020f	51	145	26	145	20.2%	1.96 [1.30, 2.96]
Hu 2020b	43	87	10	87	18.2%	4.30 [2.31, 8.00]
Wan 2020	43	84	6	84	16.4%	7.17 [3.22, 15.93]
Du 2020	14	26	1	26	7.2%	14.00 [1.98, 98.85]
**Total (95% CI)**		**431**		**431**	**100.0%**	**2.50 [1.32, 4.73]**
Total events	176		66			

Heterogeneity: Tau² = 0.48; Chi² = 25.95, df = 6 (P = 0.0002); I² = 77%
Test for overall effect: Z = 2.81 (P = 0.005)

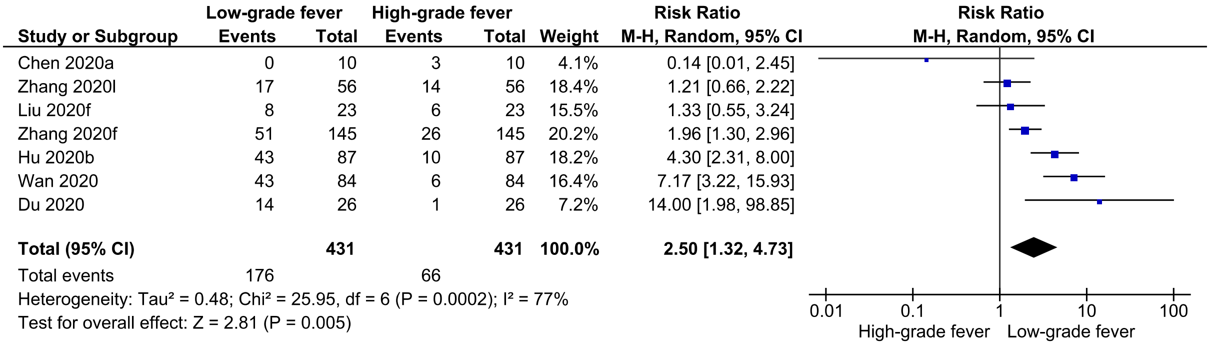

C

Study or Subgroup	Medium-grade fever Events	Medium-grade fever Total	High-grade fever Events	High-grade fever Total	Weight	Risk Ratio M-H, Random, 95% CI
Liu 2020f	9	23	6	23	12.0%	1.50 [0.64, 3.53]
Zhang 2020l	25	56	14	56	20.1%	1.79 [1.04, 3.06]
Chen 2020a	7	10	3	10	9.2%	2.33 [0.83, 6.54]
Zhang 2020f	68	145	26	145	25.6%	2.62 [1.77, 3.86]
Hu 2020b	34	87	10	87	17.0%	3.40 [1.79, 6.44]
Wan 2020	35	84	6	84	12.9%	5.83 [2.59, 13.13]
Du 2020	11	26	1	26	3.1%	11.00 [1.53, 79.16]
**Total (95% CI)**		**431**		**431**	**100.0%**	**2.72 [1.89, 3.90]**
Total events	189		66			

Heterogeneity: Tau² = 0.09; Chi² = 10.51, df = 6 (P = 0.10); I² = 43%
Test for overall effect: Z = 5.41 (P < 0.00001)

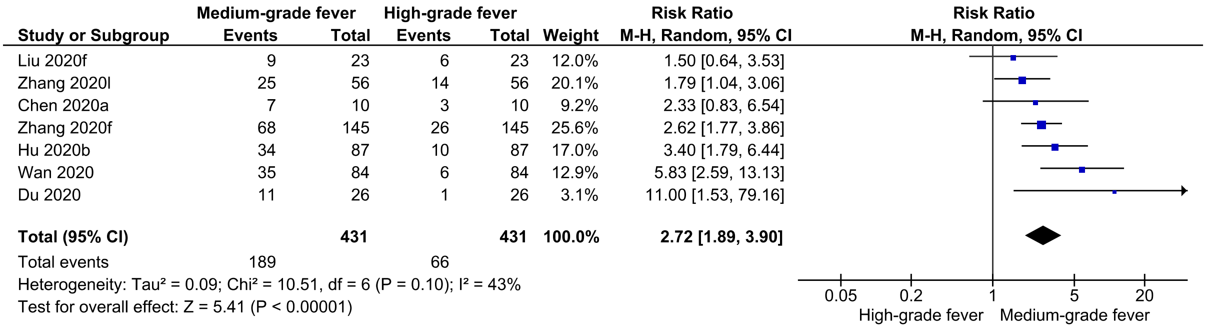

S11 Fig. Risks of (A) low-grade fever (37·3-38·0°C) vs medium-grade fever (38·1-39·0°C), (B) high-grade fever (>39·0°C) vs low-grade fever (37·3-38·0°C), and (C) high-grade fever (>39·0°C) vs medium-grade fever (38·1-39·0°C) in adult non-severe COVID-19 patients.
