# Supplementary material for: Prevalence and characteristics of fever in adult and paediatric patients with coronavirus disease 2019 (COVID-19): A systematic review and meta-analysis of 17515 patients
Source: PLoS One. 2021 Apr 6;16(4):e0249788. doi: 10.1371/journal.pone.0249788 (PMC8023501; doi:10.1371/journal.pone.0249788)
Supplement: S12 Fig — (PDF) [file pone.0249788.s013.pdf]

A

Study or Subgroup	Medium-grade fever Events	Total	Low-grade fever Events	Total	Weight	Risk Ratio M-H, Random, 95% CI
Li 2020f	20	54	32	54	39.5%	0.63 [0.41, 0.94]
Pan 2020	7	18	6	18	19.9%	1.17 [0.49, 2.79]
Li 2020a	30	60	25	60	40.6%	1.20 [0.81, 1.78]
**Total (95% CI)**		**132**		**132**	**100.0%**	**0.92 [0.57, 1.50]**
Total events	57		63			

Heterogeneity: Tau² = 0.11; Chi² = 5.41, df = 2 (P = 0.07); I² = 63%
Test for overall effect: Z = 0.33 (P = 0.74)

Risk Ratio M-H, Random, 95% CI

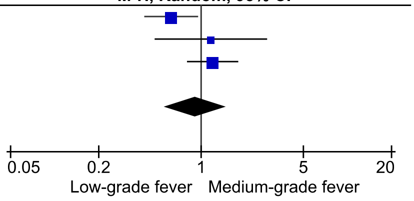

B

Study or Subgroup	Low-grade fever Events	Total	High-grade fever Events	Total	Weight	Risk Ratio M-H, Random, 95% CI
Pan 2020	6	18	5	18	34.5%	1.20 [0.45, 3.23]
Li 2020a	25	60	5	60	35.6%	5.00 [2.05, 12.19]
Li 2020f	32	54	2	54	30.0%	16.00 [4.03, 63.46]
**Total (95% CI)**		**132**		**132**	**100.0%**	**4.33 [1.02, 18.45]**
Total events	63		12			

Heterogeneity: Tau² = 1.33; Chi² = 11.05, df = 2 (P = 0.004); I² = 82%
Test for overall effect: Z = 1.98 (P = 0.05)

Risk Ratio M-H, Random, 95% CI

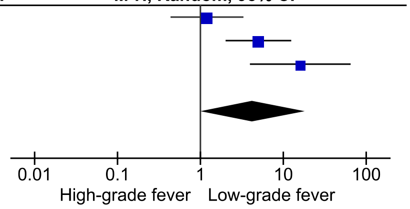

C

Study or Subgroup	Medium-grade fever Events	Total	High-grade fever Events	Total	Weight	Risk Ratio M-H, Random, 95% CI
Pan 2020	7	18	5	18	35.5%	1.40 [0.54, 3.60]
Li 2020a	30	60	5	60	36.6%	6.00 [2.50, 14.42]
Li 2020f	20	54	2	54	28.0%	10.00 [2.46, 40.70]
**Total (95% CI)**		**132**		**132**	**100.0%**	**4.13 [1.25, 13.68]**
Total events	57		12			

Heterogeneity: Tau² = 0.82; Chi² = 7.79, df = 2 (P = 0.02); I² = 74%
Test for overall effect: Z = 2.32 (P = 0.02)

Risk Ratio M-H, Random, 95% CI

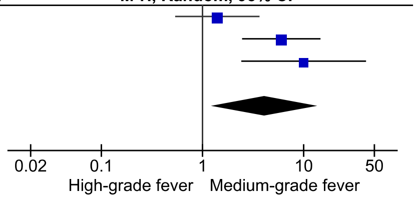

S12 Fig. Risks of (A) low-grade fever (37·3-38·0°C) vs medium-grade fever (38·1-39·0°C), (B) high-grade fever (>39·0°C) vs low-grade fever (37·3-38·0°C), and (C) high-grade fever (>39·0°C) vs medium-grade fever (38·1-39·0°C) in survived (recovered or discharged) adult COVID-19 patients.
