# Supplementary material for: Prevalence and characteristics of fever in adult and paediatric patients with coronavirus disease 2019 (COVID-19): A systematic review and meta-analysis of 17515 patients
Source: PLoS One. 2021 Apr 6;16(4):e0249788. doi: 10.1371/journal.pone.0249788 (PMC8023501; doi:10.1371/journal.pone.0249788)
Supplement: S13 Fig — (PDF) [file pone.0249788.s014.pdf]

A

Study or Subgroup	Medium-grade fever Events	Medium-grade fever Total	Low-grade fever Events	Low-grade fever Total	Weight	Risk Ratio M-H, Random, 95% CI
Fan 2020	42	91	33	91	55.4%	1.27 [0.90, 1.81]
Li 2020a	34	59	17	59	44.6%	2.00 [1.27, 3.16]
**Total (95% CI)**		**150**		**150**	**100.0%**	**1.56 [1.00, 2.42]**
Total events	76		50			

Heterogeneity: Tau² = 0.06; Chi² = 2.37, df = 1 (P = 0.12); I² = 58%
Test for overall effect: Z = 1.97 (P = 0.05)

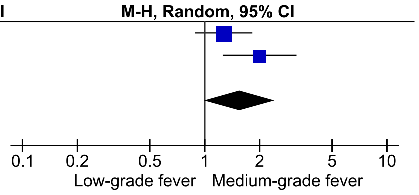

B

Study or Subgroup	Low-grade fever Events	Low-grade fever Total	High-grade fever Events	High-grade fever Total	Weight	Risk Ratio M-H, Random, 95% CI
Fan 2020	33	91	16	91	67.9%	2.06 [1.22, 3.47]
Li 2020a	17	59	8	59	32.1%	2.13 [0.99, 4.54]
**Total (95% CI)**		**150**		**150**	**100.0%**	**2.08 [1.35, 3.20]**
Total events	50		24			

Heterogeneity: Tau² = 0.00; Chi² = 0.00, df = 1 (P = 0.95); I² = 0%
Test for overall effect: Z = 3.34 (P = 0.0008)

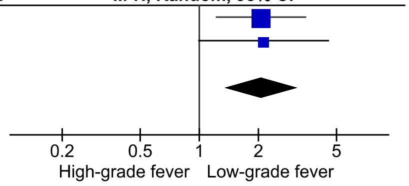

C

Study or Subgroup	Medium-grade fever Events	Medium-grade fever Total	High-grade fever Events	High-grade fever Total	Weight	Risk Ratio M-H, Random, 95% CI
Fan 2020	42	91	16	91	62.0%	2.63 [1.60, 4.32]
Li 2020a	34	59	8	59	38.0%	4.25 [2.15, 8.39]
**Total (95% CI)**		**150**		**150**	**100.0%**	**3.15 [1.99, 4.99]**
Total events	76		24			

Heterogeneity: Tau² = 0.02; Chi² = 1.27, df = 1 (P = 0.26); I² = 21%
Test for overall effect: Z = 4.89 (P < 0.00001)

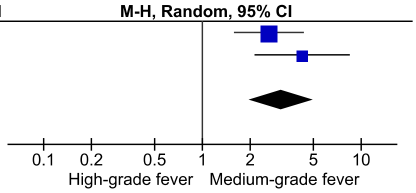

S13 Fig. Risks of (A) low-grade fever (37·3-38·0°C) vs medium-grade fever (38·1-39·0°C), (B) high-grade fever (>39·0°C) vs low-grade fever (37·3-38·0°C), and (C) high-grade fever (>39·0°C) vs medium-grade fever (38·1-39·0°C) in non-survived adult COVID-19 patients.
