# Supplementary material for: Prevalence and characteristics of fever in adult and paediatric patients with coronavirus disease 2019 (COVID-19): A systematic review and meta-analysis of 17515 patients
Source: PLoS One. 2021 Apr 6;16(4):e0249788. doi: 10.1371/journal.pone.0249788 (PMC8023501; doi:10.1371/journal.pone.0249788)
Supplement: S15 Fig — (PDF) [file pone.0249788.s016.pdf]

A

Study ID	Cases	Total	Prevalence	95% C.I.
**Excluding low-quality studies (Paediatric)**				
Du 2020a	5	14	35.71	[10.61; 60.81]
Feng 2020a	5	15	33.33	[9.48; 57.19]
Jiehao 2020	6	10	60.00	[29.64; 90.36]
Liu 2020e	3	4	75.00	[32.57; 100.00]
Qiu 2020	13	36	36.11	[20.42; 51.80]
Sun 2020	6	8	75.00	[44.99; 100.00]
Tang 2020	11	26	42.31	[23.32; 61.30]
Wang 2020b	20	31	64.52	[47.67; 81.36]
Wu 2020c	20	74	27.03	[16.91; 37.15]
Xia 2020	12	20	60.00	[38.53; 81.47]
Xu 2020d	7	10	70.00	[41.60; 98.40]
Zheng 2020a	13	25	52.00	[32.42; 71.58]
Zhou 2020d	5	9	55.56	[23.09; 88.02]
**Random effects model**		**282**	**49.94**	**[40.10; 59.77]**

Heterogeneity: $I^2 = 63\%$, $\tau^2 = 0.0186$, $\chi^2_{12} = 32.39$ ($p < 0.01$)

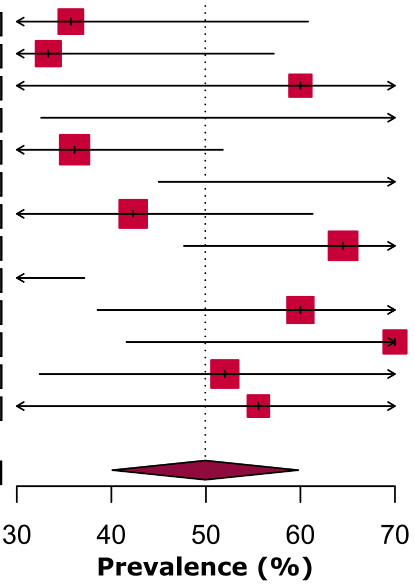

B

Study ID	Cases	Total	Prevalence	95% C.I.
**Excluding studies without RT-PCR confirmation or method not reported (Paediatric)**				
Du 2020a	5	14	35.71	[10.61; 60.81]
Feng 2020a	5	15	33.33	[9.48; 57.19]
Jiehao 2020	6	10	60.00	[29.64; 90.36]
Liu 2020e	3	4	75.00	[32.57; 100.00]
Qiu 2020	13	36	36.11	[20.42; 51.80]
Sun 2020	6	8	75.00	[44.99; 100.00]
Tang 2020	11	26	42.31	[23.32; 61.30]
Wang 2020b	20	31	64.52	[47.67; 81.36]
Wu 2020c	20	74	27.03	[16.91; 37.15]
Xu 2020d	7	10	70.00	[41.60; 98.40]
Yu 2020b	14	82	17.07	[8.93; 25.22]
Zheng 2020a	13	25	52.00	[32.42; 71.58]
Zhou 2020d	5	9	55.56	[23.09; 88.02]
**Random effects model**		**344**	**46.37**	**[34.94; 57.80]**

Heterogeneity: $I^2 = 79\%$, $\tau^2 = 0.0305$, $\chi^2_{12} = 56.48$ ($p < 0.01$)

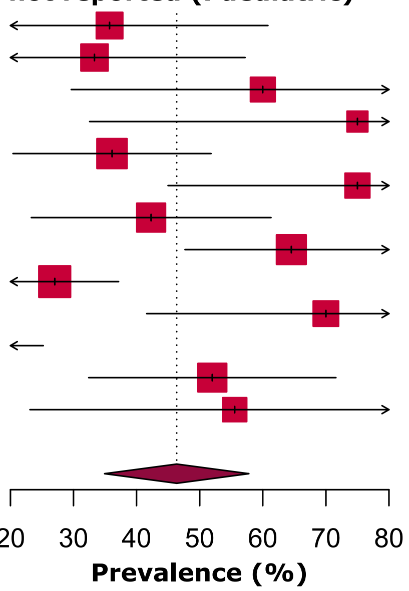

C

Study ID	Cases	Total	Prevalence	95% C.I.
**Excluding non-English studies (Paediatric)**				
Du 2020a	5	14	35.71	[10.61; 60.81]
Feng 2020a	5	15	33.33	[9.48; 57.19]
Jiehao 2020	6	10	60.00	[29.64; 90.36]
Liu 2020e	3	4	75.00	[32.57; 100.00]
Qiu 2020	13	36	36.11	[20.42; 51.80]
Su 2020	2	9	22.22	[0.00; 49.38]
Sun 2020	6	8	75.00	[44.99; 100.00]
Tang 2020	11	26	42.31	[23.32; 61.30]
Wu 2020c	20	74	27.03	[16.91; 37.15]
Xia 2020	12	20	60.00	[38.53; 81.47]
Xu 2020d	7	10	70.00	[41.60; 98.40]
Yu 2020b	14	82	17.07	[8.93; 25.22]
Zheng 2020a	13	25	52.00	[32.42; 71.58]
Zhou 2020d	5	9	55.56	[23.09; 88.02]
**Random effects model**		**342**	**43.93**	**[33.51; 54.35]**

Heterogeneity: $I^2 = 74\%$, $\tau^2 = 0.0254$, $\chi^2_{13} = 50.14$ ($p < 0.01$)

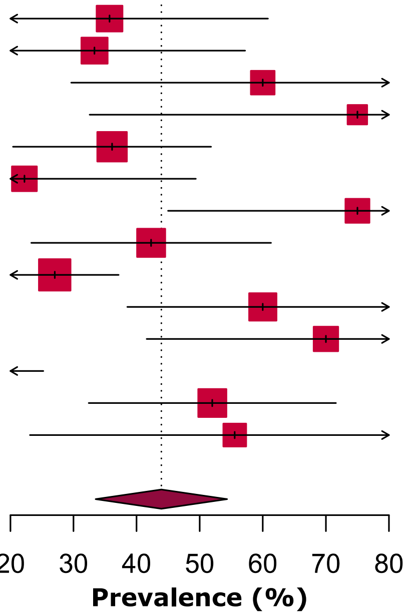

S15 Fig. Sensitivity analyses: Prevalence of fever in paediatric COVID-19 patients (A) excluding low-quality studies, (B) excluding non-English studies, and (C) considering only cross-sectional studies.
