# Supplementary material for: Prevalence and characteristics of fever in adult and paediatric patients with coronavirus disease 2019 (COVID-19): A systematic review and meta-analysis of 17515 patients
Source: PLoS One. 2021 Apr 6;16(4):e0249788. doi: 10.1371/journal.pone.0249788 (PMC8023501; doi:10.1371/journal.pone.0249788)
Supplement: S1 Table — (DOCX) [file pone.0249788.s017.docx]

| **S1 Table. Keywords used to search databases.** | |
| --- | --- |
| **Databases** | **Search terms** |
| PubMed | (((((((COVID-19[Title]) OR COVID19[Title]) OR coronavirus[Title]) OR nCoV[Title]) OR SARS-CoV-2[Title]) OR SARS-CoV2[Title])) AND ((((((((((((((((((clinical[Title/Abstract]) OR symptom[Title/Abstract]) OR symptoms[Title/Abstract]) OR characteristic[Title/Abstract]) OR characteristics[Title/Abstract]) OR feature[Title/Abstract]) OR features[Title/Abstract]) OR condition[Title/Abstract]) OR conditions[Title/Abstract]) OR comorbid[Title/Abstract]) OR co-morbid[Title/Abstract]) OR comorbidity[Title/Abstract]) OR co-morbidity[Title/Abstract]) OR comorbidities[Title/Abstract]) OR co-morbidities[Title/Abstract]) OR epidemiological[Title/Abstract]) OR epidemiology[Title/Abstract]) OR fever[Title/Abstract]) |
| Scopus | TITLE-ABS(COVID-19 OR COVID19 OR coronavirus OR nCoV OR SARS-CoV-2 OR SARS-CoV2) AND TITLE-ABS(clinical OR symptom OR symptoms OR characteristic OR characteristics OR feature OR features OR condition OR conditions OR comorbid OR co-morbid OR comorbidity OR co-morbidity OR comorbidities OR co-morbidities OR epidemiological OR epidemiology OR fever) AND ( LIMIT-TO ( PUBYEAR,2020) OR LIMIT-TO ( PUBYEAR,2019)) |
| ScienceDirect | Title, abstract, keywords: (COVID-19 OR COVID19 OR coronavirus OR nCoV OR SARS-CoV-2 OR SARS-CoV2) AND (clinical OR symptom OR symptoms OR characteristic OR characteristics OR feature OR features OR condition OR conditions OR comorbid OR co-morbid OR comorbidity OR co-morbidity OR comorbidities OR co-morbidities OR epidemiological OR epidemiology OR fever) |
| Google Scholar | allintitle:(COVID-19 OR COVID19 OR coronavirus OR nCoV OR SARS-CoV-2 OR SARS-CoV2) (clinical OR symptom OR symptoms OR characteristic OR characteristics OR feature OR features OR condition OR conditions OR comorbid OR co-morbid OR comorbidity OR co-morbidity OR comorbidities OR co-morbidities OR epidemiological OR epidemiology OR fever) |
