# Supplementary material for: Prevalence and characteristics of fever in adult and paediatric patients with coronavirus disease 2019 (COVID-19): A systematic review and meta-analysis of 17515 patients
Source: PLoS One. 2021 Apr 6;16(4):e0249788. doi: 10.1371/journal.pone.0249788 (PMC8023501; doi:10.1371/journal.pone.0249788)
Supplement: S2 Table — (DOCX) [file pone.0249788.s018.docx]

| **S2 Table. Major characteristics of the included studies.** | | | | | | | | | |  |
| --- | --- | --- | --- | --- | --- | --- | --- | --- | --- | --- |
| **No.** | **Study ID**  **(Reference)** | **Country**  **(province/** **municipalities/** **special administrative regions / city)** | **Data collection period** | **COVID-19 confirmation procedure** | **Total number of COVID-19 patients (female)** | **Age category of COVID-19 patients** | **Age (years) (mean±SD / median (IQR) / range** | **Subgroups of COVID-19 patients** | **Body temperature (°C) (mean±SD / median (IQR) / range** | |
| 1 | Ai 2020  [1] | China  (Hubei) | Until 9 Feb, 2020 | RT-PCR | 102 (50) | Adult (n=100) and paediatric (n=2) | 50·3±16·8 | None | 37·2-38·5 | |
| 2 | Bhatraju 2020  [2] | USA  (Seattle) | 24 Feb to 9 Mar, 2020 | RT-PCR | 24 (9) | Adult | 64·0±18·0 | All critical ICU patients | NR | |
| 3 | Bian 2020  [3] | China  (Shaanxi) | 3 Feb to 10 Feb, 2020 | RT-PCR | 28 (6) | Adult | 51·0 (49·0-67·0) | Severe or critical (n=20) and non-severe (n=8) | NR | |
| 4 | Cao 2020  [4] | China  (Hubei) | 18 Jan to 3 Feb, 2020 | RT-PCR | 199 (79) | Adult | 58·0 (49·0-68·0) | All severe patients | 36·5 (36·4-36·8) | |
| 5 | Cao 2020a  [5] | China  (Shanghai) | 20 Jan to 15 Feb, 2020 | RT-PCR | 198 (97) | Adult | 50·1±16·3 | ICU (n=19) and non-ICU (n=179) | 38·3 (38·0-38·9) | |
| 6 | Cao 2020b  [6] | China  (Hubei) | 1 Jan to 16 Feb, 2020 | RT-PCR | 128 (68) | Adult (n=126) and paediatric (n=2) | 0·0-65·0+ | Severe (n=21) and non-severe (n=107) | NR | |
| 7 | Chang 2020  [7] | China  (Hebei) | 16 Jan to  29 Jan, 2020 | RT-PCR | 13 (NR) | Adult (n=11) and paediatric (n=2) | 34·0 (34·0-48·0) | None | 38·4±0·8 | |
| 8 | Chang 2020a  [8] | China  (Beijing) | 28 Jan to 9 Feb, 2020 | RT-PCR | 16 (5) | Adult | 35·5 (24·0-43·0) | None | NR | |
| 9 | Chen 2020  [9] | China  (Hubei) | 28 Feb to 4 Mar, 2020 | RT-PCR | 31 (16) | Adult | 18·0- 86·0 | Severe or critical (n=5) and non-severe (n=26) | NR | |
| 10 | Chen 2020a  [10] | China  (Hubei) | 19 Dec, 2019 to 27 Jan, 2020 | RT-PCR | 21 (4) | Adult | 56·3±14·3 | Severe (n=11) and non-severe (n=10) | 38·8±0·5 | |
| 11 | Chen 2020b  [11] | China  (Shanghai) | 20 Jan to 6 Feb, 2020 | RT-PCR | 249 (123) | Adult | 51·0 (36·0-64·0) | ICU (n=22) and non-ICU (n=227) | NR | |
| 12 | Chen 2020c  [12] | China  (Hubei) | 20 Jan to 30 Jan, 2020 | RT-PCR | 9 (9) | Adult | 29·9±4·8 | New mothers | NR | |
| 13 | Chen 2020d  [13] | China  (Hubei) | 16 Jan to 29 Jan, 2020 | RT-PCR | 89 (59) | Adult | 33·4±9·1 | None | NR | |
| 14 | Chen 2020e  [14] | China  (Hubei) | 1 Feb to 1 Mar, 2020 | RT-PCR | 534 (266) | Adult | Mobile cabin hospital: 40·0 (16·0-68·0); hospital: 50·0 (19·0-65·0) | Mobile cabin hospital (n=263) and hospital (n=271) | NR | |
| 15 | Chen 2020f  [15] | China  (Hubei) | 1 Jan to 20 Jan, 2020 | RT-PCR | 99 (32) | Adult | 55·5±13·1 | None | NR | |
| 16 | Chen 2020g  [16] | China  (Hubei) | 30 Jan to 23  Feb, 2020 | RT-PCR | 17 (17) | Adult | 29·1±2·4 | New mothers | NR | |
| 17 | Chen 2020h  [17] | China  (Hunan) | 23 Jan to 14  Feb, 2020 | RT-PCR | 291 (146) | Adult (n=280) and paediatric (n=11) | 46·0 (34·0-59·0) | Severe or critical (n=50) and non-severe (n=241) | 36·9 (36·5-37·4) | |
| 18 | Chen 2020i  [18] | China  (Hebei) | 22 Jan to 14 Feb, 2020 | RT-PCR | 37 (16) | Adult | 58·7±13·7 | All severe or critical patients | 37·9±0·7 | |
| 19 | Chen 2020j  [19] | China  (Hubei) | Jan to Mar, 2020 | RT-PCR | 30 (16) | Adult | 60·5 (32·0-77·0) | None | NR | |
| 20 | Chen 2020k  [20] | China  (Hubei) | 5 Jan to 7 Mar, 2020 | RT-PCR | 123 (73) | Adult | 51·0 (35·0-66·0) | With HBV  infection  (n=15) and  without HBV  infection  (n=108) | 37·4-39·1 | |
| 21 | Chen 2020l  [21] | China  (Hubei) | 13 Jan to 12 Feb, 2020 | RT-PCR | 274 (103) | Adult | 62·0 (44·0-70·0) | Survived (n=161) and non-survived (n=274) | NR | |
| 22 | Chen 2020m  [22] | China  (Hubei) | 20 Jan to 10 Feb, 2020 | RT-PCR | 5 (5) | Adult | 25·0-31·0 | New mothers | 37·4-38·5 | |
| 23 | Chen 2020n  [23] | China  (Hubei) | 20 Feb to 12 Mar, 2020 | RT-PCR | 236 (126) | Adult | NR | None | NR | |
| 24 | Cheng 2020  [24] | China  (Hubei) | 28 Jan to 11  Feb, 2020 | NR | 701 (334) | Adult | 63·0 (50·0-71·0) | Normal (n=600) and elevated (n=101) serum  creatinine | NR | |
| 25 | Cheng 2020a  [25] | China  (Shanghai) | 19 Jan to 6  Feb, 2020 | RT-PCR | 11 (3) | Adult | 50·3±15·5 | None | 37·9±0·7 | |
| 26 | Cheng 2020b  [26] | China  (Hubei) | 15 Jan to 23 Feb, 2020 | RT-PCR | 111 (111) | Adult | 32·0 (22·0-41·0) | Pregnant  (n=31) and  non-pregnant  (n=80) | NR | |
| 27 | Chu 2020  [27] | China  (Hubei) | 7 Jan to 11 Feb, 2020 | RT-PCR | 54 (18) | Adult | 26·0-73·0 | Severe (n=43) and non-severe (n=11) | NR | |
| 28 | Chuan 2020  [28] | China  (Shaanxi, Gansu, Liaoning, Zhejiang and Hebei) | 23 Jan to 8  Feb, 2020 | RT-PCR | 32 (12) | Adult | 38·5 (26·2-45·7) | Severe or critical (n=4) and non-severe (n=28) | NR | |
| 29 | Chung 2020  [29] | China  (Guangdong, Jiangxi and Shandong) | 18 Jan to 27 Jan, 2020 | NR | 21 (8) | Adult | 51·0±41·0 | None | NR | |
| 30 | Cui 2020  [30] | China  (Hubei) | 28 Jan to 18  Feb, 2020 | RT-PCR | 35 (35) | Adult | 61·5±11·2 | All female, severe or critical (n=34) and non-severe (n=1) | NR | |
| 31 | Deng 2020  [31] | China  (Hubei) | 1 Jan to 21 Feb, 2020 | RT-PCR | 225 (101) | Adult | Non-survived: 69·0 (62·0-74·0) and survived 40·0 (33·0-57·0) | Survived (n=116) and non-survived (n=109) | NR | |
| 32 | Diao 2020  [32] | China  (Sichuan) | 17 Jan to 5  Feb, 2020 | RT-PCR | 6 (3) | Adult | 42·7±14·1 | None | NR | |
| 33 | Ding 2020  [33] | China  (Hubei) | NR | NR | 5 (3) | Adult | 39·0-66·0 | None | NR | |
| 34 | Du 2020  [34] | China  (Hubei) | 25 Dec to 7  Feb, 2020 | RT-PCR | 179 (82) | Adult | 57·6±13·7 | Severe or critical (n=100) and non-severe (n=79) | NR | |
| 35 | Du 2020a  [35] | China  (Shandong) | 23 Jan to 25  Feb, 2020 | RT-PCR | 67 (35) | Adult (n=53) and paediatric (n=14) | Adult: 41·4 (median); paediatric: 6·2 (median) | Severe (n=1) and non-severe (n=66) | NR | |
| 36 | Easom 2020  [36] | UK  (Yorkshire) | 29 Jan to 24 Feb, 2020 | RT-PCR | 68 (36) | Adult (n=66) and paediatric (n=2) | 0·5-76·0 | Antimicrobial / medical admission group (n=14) and remaining patients (n=52) | 33·5-38·8 | |
| 37 | Fan 2020  [37] | China  (Hubei) | 30 Dec to 16  Feb, 2020 | RT-PCR | 101 (37) | Adult | 65·4±9·7 | All non-survived patients | NR | |
| 38 | Fan 2020a  [38] | China  (Shanghai) | 20 Jan to 31 Jan, 2020 | RT-PCR | 148 (73) | Adult | 50·5 (36·0-64·0) | None | NR | |
| 39 | Feng 2020  [39] | China  (Hunan) | 17 Jan to 1  Feb, 2020 | RT-PCR | 141 (69) | Adult | 44·0 (34·0-55·0) | Severe (n=15) and non-severe (n=126) | NR | |
| 40 | Feng 2020a  [40] | China  (Guangdong) | 16 Jan to 6 Feb, 2020 | RT-PCR | 15 (10) | Paediatric | 7·0 (4·0-14·0) | None | NR | |
| 41 | Fu 2020  [41] | China  (Sichuan) | 1 Jan to 20 Feb, 2020 | RT-PCR | 52 (24) | Adult | 44·5 (33·0-56·5) | Severe or critical (n=14) and non-severe (n=38) | NR | |
| 42 | Guan 2020  [42] | China  (30 provinces / autonomous regions / provincial municipalities) | 11 Dec to 29  Jan, 2020 | RT-PCR | 1096 (459) | Adult (n=1002) and paediatric (n=9) | 47·0 (35·0-58·0) | Severe (n=173) and non-severe (n=926) | 37·3 (36·7-38·0) | |
| 43 | Guan 2020a  [43] | China  (31 provinces / autonomous regions / provincial municipalities) | 21 Nov, 2019 to 31 Jan, 2020 | RT-PCR | 1590 (686) | Adult | 48·9±16·3 | With comorbidity (n=399) or without comorbidity (n=1191) | 37·4±0·9 | |
| 44 | Han 2020  [44] | China  (Hubei) | 4 Jan to 3  Feb, 2020 | RT-PCR | 108 (70) | Adult | 21·0-90·0 | None | NR | |
| 45 | Han 2020a  [45] | China  (Hubei) | 20 Dec to 2  Feb, 2020 | RT-PCR | 17 (11) | Adult | 40·0±10·0 | None | 37·9±0·9 | |
| 46 | He 2020  [46] | China  (Hubei) | 23 Jan to 14 Feb, 2020 | RT-PCR | 31 (18) | Adult | 34·0 (25·5-47·0) | With HDs (n=15) and without HDs (n=16) | NR | |
| 47 | Hou 2020  [47] | China  (Hubei) | 13 Jan to 23 Jan, 2020 | RT-PCR | 14 (9) | Adult | 59·0 (56·0-74·0) | None | NR | |
| 48 | Hu 2020  [48] | China  (Jiangsu) | 28 Jan to 9  Feb, 2020 | RT-PCR | 24 (16) | Adult (n=19) and paediatric (n=5) | 5·0-95·0 | Asymptomatic patients | NR | |
| 49 | Hu 2020a  [49] | China  (Chongqing) | 28 Jan to 24  Feb, 2020 | RT-PCR | 164 (66) | Adult | NR | Severe (n=28) and non-severe (n=136) | NR | |
| 50 | Hu 2020b  [50] | China  (Hubei) | 8 Jan to 10 March, 2020 | RT-PCR | 323 (157) | Adult | 61·0 (23·0-91·0) | Severe (n=151) and non-severe or critical (n=172) | NR | |
| 51 | Huang 2020  [51] | China  (Hubei) | 16 Dec to 2  Jan, 2020 | RT-PCR | 41 (11) | Adult | 49·0 (41·0-58·0) | ICU (n=13) and non-ICU (n=28) | NR | |
| 52 | Huang 2020a  [52] | China  (Chongqing) | 21 Jan to 8 Feb, 2020 | RT-PCR | 197 (88) | Adult (n=5) and paediatric (n=192) | 49·0 (41·0-58·0) | ICU (n=55) and non-ICU (n=142) | NR | |
| 53 | Huang 2020b  [53] | China  (Jiangsu) | 22 Jan to 10 February, 2020 | RT-PCR | 221 (95) | Adult | 45·0 (33·5-56·0) | Severe or critical (n=25) and non-severe (n=196) | NR | |
| 54 | Huang 2020c  [54] | China  (Hubei) | 21 Jan 21 to 10 Feb, 2020 | RT-PCR | 36 (11) | Adult | 69·2±9·6 | All non-survived patients | NR | |
| 55 | Huang 2020d  [55] | China  (Hubei) | 21 Dec to 28 Jan, 2020 | RT-PCR | 34 (20) | Adult | 56·2±17·1 | None | 38·6±0·8 | |
| 56 | Ji 2020  [56] | China  (Zhejiang) | 21 Dec to 28 Jan, 2020 | RT-PCR | 33 (16) | Adult | 44·6±12·6 | None | 38·1±0·5 | |
| 57 | Jiehao 2020  [57] | China  (Shanghai, Hainan, Anhui and Shandong) | 19 Jan to 3 Feb, 2020 | RT-PCR | 10 (6) | Paediatric | 0·2-10·9 | None | 37·7-39·2 | |
| 58 | Jin 2020  [58] | China  (Zhejiang) | 17 Jan to 8  Feb, 2020 | RT-PCR | 651 (320) | Adult | 45·6±14·3 | With GI symptoms (n=74) and without GI symptoms (n=577) | NR | |
| 59 | Lei 2020  [59] | China  (Hubei) | 14 Jan to 29 Jan, 2020 | NR | 29 (8) | Adult | 26·0-79·0 | Severe or critical (n=14) and non-severe (n=15) | NR | |
| 60 | Lei 2020a  [60] | China  (Sichuan) | 4 Jan to 28 Feb, 2020 | RT-PCR | 67 (28) | Adult (n=59) and paediatric (n=8) | 39·3±18·3 | Asymptomatic (n=36) and symptomatic (n=31) | NR | |
| 61 | Lei 2020b  [61] | China  (Guangdong) | 22 Jan to 12 Feb, 2020 | RT-PCR | 20 (10) | Adult | 43·2±14·0 | None | NR | |
| 62 | Lescure 2020  [62] | France  (Paris) | 24 Jan to 14 Feb, 2020 | RT-PCR | 5 (2) | Adult | 47·0±20·2 | None | NR | |
| 63 | Li 2020  [63] | China  (Sichuan) | 22 Jan to 10 Feb, 2020 | RT-PCR | 17 (8) | Adult | 45·1±12·8 | Discharged (n=5) and non-discharged (n=5) | NR | |
| 64 | Li 2020a  [64] | China  (Hubei) | 21 Jan to 14 Feb, 2020 | RT-PCR | 161 (58) | Adult | Survived: 46·0 (22·0-87·0) and non-survived 67·0 (31·0-87·0) | Survived (n=96) and non-survived (n=65) | NR | |
| 65 | Li 2020b  [65] | China  (Beijing) | 8 Feb to 22 Feb, 2020 | RT-PCR | 47 (19) | Adult | 62·0 (51·0-70·0) | All severe patients | NR | |
| 66 | Li 2020c  [66] | China  (Chongqing) | 1 Jan to 29 Feb, 2020 | RT-PCR | 83 (39) | Adult | 45·5±12·3 | Severe or critical (n=25) and non-severe (n=58) | 37·8±0·7 | |
| 67 | Li 2020d  [67] | China  (Hubei) | 28 Jan to 26 Feb, 2020 | RT-PCR | 77 (24) | Adult | 70·0 (62·0-78·0) | Respiratory failure in the terminal stage | NR | |
| 68 | Li 2020e  [68] | China  (Beijing) | 19 Jan to 22 Feb, 2020 | RT-PCR | 18 (7) | Adult | 39·0 (34·0-60·2) | None | 38·0 (37·6-38·5) | |
| 69 | Li 2020f  [69] | China  (Guangdong) | 18 Jan to 7 Feb, 2020 | RT-PCR | 78 (40) | Adult | 44·6±17·9 | None | NR | |
| 70 | Lian 2020  [70] | China  (Zhejiang) | 17 Jan to 12 Feb, 2020 | RT-PCR | 788 (381) | Adult | 54·7±9·3 | Older (≥ 60 years) (n=136) and younger (< 60 years) (n=652) | NR | |
| 71 | Liang 2020  [71] | China  (Beijing) | 21 Jan to 15 Feb, 2020 | RT-PCR | 21 (10) | Adult | 42·0 (34·5-66·0) | None | NR | |
| 72 | Liao 2020  [72] | China  (Chongqing) | 25 Jan to 18 Feb, 2020 | RT-PCR | 32 (15) | Adult | 25·0-35·0 | None | NR | |
| 73 | Liu 2020  [73] | China  (Hainan) | 1 Jan to 16 Feb, 2020 | NR | 51 (23) | Adult | 50·4±13·0 | None | NR | |
| 74 | Liu 2020a  [74] | China  (Chongqing) | 20 Jan to 3 Feb, 2020 | NR | 51 (19) | Adult | 45·0 (34·0-51·0) | Severe (n=7) and non-severe (n=44) | NR | |
| 75 | Liu 2020b  [75] | China  (Guangdong) | 10 Jan to 24 Feb, 2020 | RT-PCR | 291 (158) | Adult | 48·1 (34·0-62·0) | With cardiac injury (n=15) and without cardiac injury (n=276) | 36·9 (36·6-37·5) | |
| 76 | Liu 2020c  [76] | China  (Hubei) | 20 Jan to 10 Feb, 2020 | RT-PCR | 15 (15) | Adult | 32·0±5·0 | New mothers | 37·6-39·0 | |
| 77 | Liu 2020d  [77] | China  (Zhejiang) | 22 Jan to 11  Feb, 2020 | RT-PCR | 10 (6) | Adult | 43·0±10·4 | None | 37·3-38·0 | |
| 78 | Liu 2020e  [78] | China  (Hubei) | 27 Jan to 14 Feb, 2020 | RT-PCR | 55 (50) | Adult (n=51) and paediatric (n=4) | NR | Pregnant (n=41), non-pregnant (n=10) and children (n=4) | NR | |
| 79 | Liu 2020f  [79] | China  (Hubei) | 5 Jan to 24 Jan, 2020 | RT-PCR | 40 (25) | Adult | 48·7±13·9 | Severe (n=13) and non-severe (n=27) | NR | |
| 80 | Liu 2020g  [80] | China  (Hubei) | 16 Jan to 15 Feb, 2020 | RT-PCR | 64 (41) | Adult | 35·0 (29·0-43·0) | Symptoms onset ≤10 days (n=37) and symptoms onset >10 days (n=25) | NR | |
| 81 | Liu 2020h  [81] | China  (Hubei) | 30 Dec, 2019 to 24 Jan, 2020 | RT-PCR | 137 (76) | Adult | 57·0 (20·0-83·0) | None | NR | |
| 82 | Liu 2020i  [82] | China  (Anhui) | 21 Jan to 3 Feb, 2020 | RT-PCR | 73 (32) | Adult | 42·5±14·1 | Severe or critical (n=24) and non-severe (n=49) | NR | |
| 83 | Liu 2020j  [83] | China  (Hubei, Fujian, Shanxi, Hebei, Guangdong, Jiangxi, Heilongjiang and Anhui) | 8 Dec, 2019 to 25 Jan, 2020 | RT-PCR | 13 (13) | Adult | 29·7±4·0 | Pregnant women | NR | |
| 84 | Liu 2020k  [84] | China  (Hubei) | 2 Jan to 1 Feb, 2020 | RT-PCR | 109 (50) | Adult | 55·0 (43·0-66·0) | COVID-19 with ARDS (n=53) and without ARDS (n=56) | NR | |
| 85 | Liu 2020l  [85] | China  (Hubei) | 30 Dec, 2019 to 15 Jan, 2020 | RT-PCR | 78 (39) | Adult | 38·0 (33·0-57·0) | Improvement/  stabilization (n=67) and progression (n=11) | 37·8 (37·1-38·2) | |
| 86 | Liu 2020m  [86] | China  (Guangdong) | 11 Jan to 20 Jan, 2020 | RT-PCR | 12 (4) | Adult (n=11) and paediatric (n=1) | 53·7±18·0 | None | NR | |
| 87 | Liu 2020n  [87] | China  (Hunan) | 8 Jan to 8 Feb, 2020 | RT-PCR | 24 (16) | Adult (n=23) and paediatric (n=1) | 43·0 (12·0-84·0) | None | NR | |
| 88 | Liu 2020o  [88] | China  (Jiangsu) | 10 Jan to 18 Feb, 2020 | RT-PCR | 620 (294) | Adult | 44·4±17·1 | Severe or critical (n=53) and non-severe (n=519) | 37·0± 0·7 | |
| 89 | Liu 2020p  [89] | China  (Hainan) | 1 Jan to 15 Feb, 2020 | RT-PCR | 56 (25) | Adult | 68·0 (65·2-69·7) | Young (n=38) and elderly (n=18) | NR | |
| 90 | Lo 2020  [90] | China  (Guangdong) | 21 Jan to 16 Feb, 2020 | RT-PCR | 10 (7) | Adult (n=9) and paediatric (n=1) | 54·0 (27·0-64·0) | None | NR | |
| 91 | Lu 2020  [91] | China  (Hubei) | 1 Jan to 15 Feb, 2020 | RT-PCR | 123 (62) | Adult | 62·5±11·5 | Survived (n=92) and non-survived (n=31) | 38·3±0·8 | |
| 92 | Lu 2020a  [92] | China  (Shanghai) | 20 Jan to 19 Feb, 2020 | RT-PCR | 265 (NR) | Adult | NR | Severe or critical (n=243) and non-severe (n=22) | 37·4±1·8 | |
| 93 | Lu 2020b  [93] | China  (Hubei) | 21 Jan to 10 Feb, 2020 | RT-PCR | 91 (42) | Adult | 47·6±15·6 | None | NR | |
| 94 | Luo 2020  [94] | China  (Hubei) | 17 Jan to 25 Feb, 2020 | RT-PCR | 403 (210) | Adult | 56·0 (39·0-68·0) | Survived (n=303) and non-survived (n=100) | NR | |
| 95 | Mao 2020  [95] | China  (Shanghai) | 17 Jan to 16 Feb, 2020 | RT-PCR | 188 (94) | Adult | 46·7±16·1 | None | 38·1±0·5 | |
| 96 | Miao 2020  [96] | China  (Shanghai) | 12 Jan to 13 Feb, 2020 | RT-PCR | 62 (30) | Adult | 43·8±13·9 | None | 37·7±0·7 | |
| 97 | Min 2020  [97] | China  (Hubei) | 3 Jan to 11 Jan, 2020 | NR | 30 (20) | Adult | 35·0±8·0 | Severe (n=22) and non-severe (n=243) | 37·4±1·8 | |
| 98 | Mo 2020  [98] | China  (Hubei) | 1 Jan to 5 Feb, 2020 | NAT | 155 (69) | Adult | 54·0 (42·0-66·0) | General (n=70) and refractory (n=85) | 38·5 (38·0-39·0) | |
| 99 | Nie 2020  [99] | China  (Hubei) | 1 Jan to 20 Feb, 2020 | RT-PCR | 33 (33) | Adult | 30·5±3·1 | Pregnant women | NR | |
| 100 | Pan 2020  [100] | China  (Hubei) | 12 Jan to 6 Feb, 2020 | RT-PCR | 21 (15) | Adult | 40·0±9·0 | None | NR | |
| 101 | Pung 2020[101] | Singapore | 18 Jan to 10 Feb, 2020 | RT-PCR | 17 (10) | Adult | 40·0 (36·0-51·0) | None | NR | |
| 102 | Qi 2020  [102] | China  (Chongqing) | 19 Jan to 16 Feb, 2020 | RT-PCR | 267 (118) | Adult | 48·0 (35·0-65·0) | Severe (n=217) and non-severe (n=50) | 37·6 (36·1-40·4) | |
| 103 | Qian 2020  [103] | China  (Hubei) | 20 Jan to 11 Feb, 2020 | RT-PCR | 91 (54) | Adult | 50·0 (36·5-57·0) | Severe (n=82) and non-severe (n=9) | NR | |
| 104 | Qin 2020  [104] | China  (Hubei) | 10 Jan to 12 Feb, 2020 | RT-PCR | 452 (217) | Adult | 58·0 (47·0-67·0) | Severe (n=286) and non-severe (n=166) | NR | |
| 105 | Qin 2020a  [105] | China  (Hubei) | 23 Jan to 11 Feb, 2020 | RT-PCR | 89 (43) | Adult | 55·0 (23·0-86·0) | ICU (n=35) and non-ICU (n=53) | NR | |
| 106 | Qiu 2020  [106] | China  (Zhejiang) | 17 Jan to 1 Mar, 2020 | RT-PCR | 36 (13) | Paediatric | 8·3±3·5 | All non-severe patients | 37·8±0·4 | |
| 107 | Qiu 2020a  [107] | China  (Hunan) | 22 Jan to 12 Feb, 2020 | RT-PCR | 104 (55) | Adult | 43·0±7·5 | NR | 36·9±0·5 | |
| 108 | Shi 2020  [108] | China  (Hubei) | 20 Dec to 23 Jan, 2020 | RT-PCR | 81 (39) | Adult | 49·5±11·0 | NR | NR | |
| 109 | Shi 2020a  [109] | China  (Hubei) | Until 15 Feb, 2020 | NR | 101 (41) | Adult | 71·0 (59·0-80·0) | All non-survived patients | 38·5 (37·8-39·0) | |
| 110 | Shu 2020  [110] | China  (Hubei) | 13 Feb to 29 Feb, 2020 | RT-PCR | 545 (281) | Adult | 50·0 (38·0- 58·0) | None | NR | |
| 111 | Song 2020  [111] | China  (Shanghai) | 20 Jan to 27 Jan, 2020 | RT-PCR | 51 (26) | Adult | 49·6±16·0 | None | NR | |
| 112 | Su 2020  [112] | China  (Shandong) | 24 Jan to 24 Feb, 2020 | NAT | 23 (12) | Adult (n=14) and paediatric (n=9) | Adult: 37·0 (median); paediatric: 3·5 (median) | None | NR | |
| 113 | Sun 2020  [113] | China  (Hubei) | 24 Jan to 24 Feb, 2020 | RT-PCR | 8 (2) | Paediatric | 0·1-15·0 | All severe or critical patients | NR | |
| 114 | Tang 2020  [114] | China  (Guangdong) | 16 Jan to 8 Feb, 2020 | RT-PCR | 26 (9) | Paediatric | 6·9±0·7 | All non-severe patients | NR | |
| 115 | Tang 2020a  [115] | China  (Hubei) | 24 Dec, 2019 to 7 Feb, 2020 | RT-PCR | 73 (28) | Adult | 67·0 (57·0-72·0) | None | 36·8 (36·5-38·2) | |
| 116 | Tian 2020  [116] | China  (Beijing) | 20 Jan to 10 Feb, 2020 | RT-PCR | 262 (135) | Adult (n=251) and paediatric (n=11) | 47·5 (1·0-94·0) | Severe (n=46) and  non-severe (216) | NR | |
| 117 | Tian 2020a  [117] | China  (Shandong) | NR | RT-PCR | 37 (20) | Adult | 44·3±1·6 | Quarantine onset duration ≤20 days (n=19) and >20 days (n=18) | NR | |
| 118 | To 2020  [118] | China  (Guangdong) | 22 Jan to 12 Feb, 2020 | RT-PCR | 23 (10) | Adult | 62·0 (37·0-75·0) | Severe (n=10) and  non-severe (n=13) | NR | |
| 119 | Wan 2020  [119] | China  (Chongqing) | 23 Jan to 8 Feb, 2020 | RT-PCR | 135 (63) | Adult | 47·0 (36·0-55·0) | Severe (n=40) and non-severe (n=95) | NR | |
| 120 | Wang 2020  [120] | China  (Hubei) | 25 Jan to 9 Feb, 2020 | RT-PCR | 114 (56) | Adult | 53·0 (23·0-78·0) | All severe patients (n=26), early stage (n=30) and progressing stage (n=54) | NR | |
| 121 | Wang 2020a  [121] | China  (Hubei) | 1 Jan to 3 Feb, 2020 | RT-PCR | 138 (63) | Adult | 56·0 (42·0-68·0) | ICU (n=36) and non-ICU (n=102) | NR | |
| 122 | Wang 2020b  [122] | China  (Shaanxi, Gansu, Ningxia, Hebei, Henan and Shandong) | 25 Jan to 21 Feb, 2020 | RT-PCR | 31 (16) | Paediatric | 0·5-17·0 | All non-severe patients | 37·3-41·0 | |
| 123 | Wang 2020c  [123] | China  (Henan) | 21 Jan to 5 Feb, 2020 | RT-PCR | 18 (8) | Adult (n=16) and paediatric (n=2) | 39·0 (29·0-55·0) | None | NR | |
| 124 | Wang 2020d  [124] | China  (Guangdong) | 26 Jan to 19 Feb, 2020 | RT-PCR | 11 (1) | Adult | 58·0 (49·0-72·0) | All severe or critical patients | 38·5 (38-39·6） | |
| 125 | Wang 2020e  [125] | China  (Hubei) | 16 Jan to 17 Feb, 2020 | RT-PCR | 90 (57) | Adult | 45·0±14·0 | None | NR | |
| 126 | Wang 2020f  [126] | China  (Hubei) | 1 Jan to 10 Feb, 2020 | NAT | 110 (62) | Adult | NR | Severe pneumonia (n=38) and non-severe pneumonia (n=72) | NR | |
| 127 | Wang 2020g  [127] | China  (Hubei) | 16 Jan to 29 Jan, 2020 | RT-PCR | 69 (37) | Adult | 42·0 (35·0-62·0) | SpO_2_ ≥90% (n=55) and  SpO_2_ <90% (n=14) | 38·5 (38·0-38·8) | |
| 128 | Wang 2020h  [128] | China  (Fujian) | 22 Jan to 16 Feb, 2020 | RT-PCR | 165 (73) | Adult | 44·0 (33·0-59·5) | Aged > 65 years (n=29) and aged ≤ 60 years (n=136) | NR | |
| 129 | Wang 2020i  [129] | China  (Hubei) | 1 Jan to 6 Feb, 2020 | RT-PCR | 339 (173) | Adult | 69·0 (65·0-76·0) | Survived (n=174) and non-survived (n=65) | NR | |
| 130 | Wang 2020j  [130] | China  (Hubei) | 7 Jan to 14 Feb, 2020 | NAT | 60 (28) | Adult | 60·0 (38·0-66·0) | None | NR | |
| 131 | Wei 2020  [131] | China  (Hubei) | 1 Feb to 28 Feb, 2020 | RT-PCR | 100 (60) | Adult | 49·1±17·2 | All non-severe patients | NR | |
| 132 | Wei 2020a  [132] | China  (Hubei) | 19 Jan to 7 Feb, 2020 | RT-PCR | 84 (56) | Adult | 37·0 (24·0-74·0) | Diarrhoea group  (n=26)  non-diarrhoea  group  (n=58) | NR | |
| 133 | Wen 2020  [133] | China  (Guangdong) | 1 Jan to 28 Feb, 2020 | RT-PCR | 417 (220) | Adult (n=388) and paediatric (n=29) | 45·4±17·7 | Severe or critical (n=36) and non-severe (n=381) | NR | |
| 134 | Wu 2020  [134] | China  (Jiangsu) | 22 Jan to 14 Feb, 2020 | RT-PCR | 80 (41) | Adult (n=70) and paediatric (n=10) | 46·1±15·4 | Severe or critical (n=3) and non-severe (n=77) | NR | |
| 135 | Wu 2020a  [135] | China  (Jiangsu and Anhui) | 20 Jan to 20 Feb, 2020 | RT-PCR | 280 (129) | Adult (n=245) and paediatric (n=35) | 43·1±19·0 | Severe or critical (n=83) and non-severe (n=197) | NR | |
| 136 | Wu 2020b  [136] | China  (Hainan) | 20 Jan to 19 Feb, 2020 | RT-PCR | 91 (39) | Adult | 50·0±14·0 | None | 38·0 (37·5-38·7) | |
| 137 | Wu 2020c  [137] | China  (Hubei) | 20 Jan to 27 Feb, 2020 | RT-PCR | 74 (30) | Paediatric | 6·0 (0·1-15·0) | None | 38·6 (37·6-40·1) | |
| 138 | Xia 2020  [138] | China  (Hubei) | 23 Jan to 8 Feb, 2020 | NAT | 20 (7) | Paediatric | 0·2-14·7 | None | NR | |
| 139 | Xie 2020  [139] | China  (Sichuan) | 30 Jan to 5 Feb, 2020 | RT-PCR | 9 (5) | Adult | 38·3±15·2 | None | NR | |
| 140 | Xiong 2020  [140] | China  (Hubei) | 11 Jan to 5 Feb, 2020 | RT-PCR | 42 (17) | Adult | 49·5±14·1 | None | 39·9±0·8 | |
| 141 | Xu 2020  [141] | China  (Hubei) | 6 Jan to 31 Jan, 2020 | RT-PCR | 48 (35) | Adult | 39·0±13·8 | Non-hospitalized  patients (n=42) and  hospitalized  patients (n=6) | NR | |
| 142 | Xu 2020a  [142] | China  (Jiangsu) | 10 Jan to 18 Feb, 2020 | RT-PCR | 87 (41) | Adult (n=84) and paediatric (n=3) | Adult: 46·0 (median); paediatric: 1·6-11 (range) | Severe or critical (n=44) and non-severe (n=43) | NR | |
| 143 | Xu 2020b  [143] | China  (Jiangsu) | 23 Jan to 18 Feb, 2020 | RT-PCR | 51 (26) | Adult | NR | NR | NR | |
| 144 | Xu 2020c  [144] | China  (Hubei) | 10 Jan to 26 Jan, 2020 | RT-PCR | 62 (27) | Adult (n=60) and paediatric (n=2) | 41·0 (32·0-52·0) | None | NR | |
| 145 | Xu 2020d  [145] | China  (Guangdong) | 22 Jan to 20 Feb, 2020 | RT-PCR | 10 (4) | Paediatric | 0·2-15·0 | None | 37·8-39·2 | |
| 146 | Xu 2020e  [146] | China  (Hubei, Beijing and Shanghai) | 7 Feb to 28 Feb, 2020 | RT-PCR | 69 (34) | Adult | 57·0 (43·0-69·0) | Severe or critical (n=25) and non-severe (n=44) | NR | |
| 147 | Xu 2020f  [147] | China  (Beijing) | Jan to Feb, 2020 | NAT | 50 (21) | Adult (n=45) and paediatric (n=5) | 43·9±16·8 | Severe or critical (n=13) and non-severe (n=37) | NR | |
| 148 | Xu 2020g  [148] | China  (Guangdong) | 20 Jan to 6 Feb, 2020 | NAT | 21 (11) | Adult | 43·1±17·1 | Severe (n=7) and non-severe (n=14) | NR | |
| 149 | Yan 2020  [149] | China  (Hainan) | 22 Jan to 13 Mar, 2020 | RT-PCR | 168 (87) | Adult (n=160) and paediatric (n=8) | 51·0 (36·0-62·0) | Severe (n=36) and non-severe (n=132) | 36·8 (36·5-37·3) | |
| 150 | Yang 2020  [150] | China  (Shanghai) | 20 Jan to 1 Feb, 2020 | RT-PCR | 7 (2) | Adult | 47·4±14·6 | None | 39·2±0·6 | |
| 151 | Yang 2020a  [151] | China  (Hubei) | 24 Dec, 2019 to 26 Jan, 2020 | RT-PCR | 52 (17) | Adult | 59·7±13·3 | All severe or critical patients, survived  (n=20) and  non-survived  (n=32) | NR | |
| 152 | Yang 2020b  [152] | China  (Beijing) | 27 Dec, 2019 to 18 Feb, 2020 | RT-PCR | 55 (22) | Adult | 44·0 (34·0-54·0) | Severe (n=14) and non-severe (n=41) | NR | |
| 153 | Yang 2020c  [153] | China  (Shanghai) | 20 Jan to 30 Jan, 2020 | RT-PCR | 44 (19) | Adult | 48·5 (20·0-76·0) | None | NR | |
| 154 | Yao 2020  [154] | China  (Hubei) | 26 Jan to 18 Feb, 2020 | RT-PCR | 55 (18) | Adult | 70·7±13·5 | None | NR | |
| 155 | Young 2020  [155] | Singapore | 23 Jan to 25 Feb, 2020 | RT-PCR | 18 (9) | Adult | 47·0 (31·0-73·0) | Required supplemental O_2_ (n=6) and did not require  supplemental O_2_ (n=12) | 37·7 (36·1-39·6) | |
| 156 | Yu 2020  [156] | China  (Hubei) | 1 Jan to 8 Feb, 2020 | RT-PCR | 7 (7) | Adult | 32·0 (29·0-34·0) | New mothers | NR | |
| 157 | Yu 2020a  [157] | China  (Beijing) | 5 Feb to 19 Feb, 2020 | RT-PCR | 76 (38) | Adult | 40·0 (32·0-63·0) | None | NR | |
| 158 | Yu 2020b  [158] | China  (Hubei) | 1 Feb to 20 Feb, 2020 | RT-PCR | 82 (51) | Paediatric | 0·0-16·0 | None | NR | |
| 159 | Yuan 2020  [159] | China  (Guangdong) | 23 Jan to 21 Feb, 2020 | RT-PCR | 25 (17) | Adult (n=19) and paediatric (n=6) | 28·0 (16·2-42·0) | None | NR | |
| 160 | Yuan 2020a  [160] | China  (Hubei) | 1 Jan to 25 Jan, 2020 | RT-PCR | 27 (15) | Adult | 60·0 (47·0-69·0) | Survived (n=17) and non-survived (n=10) | NR | |
| 161 | Yuan 2020b  [161] | China  (Guangdong) | 5 Jan to 13 Feb, 2020 | RT-PCR | 94 (52) | Adult (n=82) and paediatric (n=12) | 40·0 (1·0-78·0) | Severe (n=11) and non-severe (n=83) | NR | |
| 162 | Yuanyuan 2020  [162] | China  (Hubei) | Jan to Feb, 2020 | RT-PCR | 31 (16) | Adult | 54·0±13·0 | Severe (n=11) and non-severe (n=20) | 38·4±1·0 | |
| 163 | Zeng 2020  [163] | China  (Guangdong) | 11 Jan to 29 Feb, 2020 | RT-PCR | 338 (176) | Adult | 49·0±14·5 | Severe (n=76) and non-severe (n=262) | NR | |
| 164 | Zhang 2020  [164] | China  (Hubei) | 11 Jan to 10 Feb, 2020 | RT-PCR | 82 (28) | Adult | 72·5 (65·0-80·0) | None | 38·8 (38·0-39·0) | |
| 165 | Zhang 2020a  [165] | China  (Hubei) | 30 Jan to 15 Feb, 2020 | RT-PCR | 81 (36) | Adult | NR | Severe or critical (n=35) and non-severe (n=46) | NR | |
| 166 | Zhang 2020b  [166] | China  (Hubei) | 16 Jan to 3 Feb, 2020 | RT-PCR | 140 (69) | Adult | 57·0 (25·0-87·0) | Severe (n=58) and non-severe (n=82) | NR | |
| 167 | Zhang 2020c  [167] | China  (Shaanxi) | 28 Jan to 24 Feb, 2020 | RT-PCR | 13 (6) | Adult | 49·6±11·0 | None | 36·4-38·6 | |
| 168 | Zhang 2020d  [168] | China  (Zhejiang) | 27 Jan to 10 Feb, 2020 | RT-PCR | 14 (7) | Adult | 18·0-87·0 | None | NR | |
| 169 | Zhang 2020e  [169] | China  (Beijing, Chongqing,  Jinan and Nanning) | 20 Jan to 20 Feb, 2020 | RT-PCR | 478 (240) | Adult (n=446) and paediatric (n=32) | 46·9 (1·0-94·0) | None | NR | |
| 170 | Zhang 2020f  [170] | China  (Hubei) | 2 Jan to 10 Feb, 2020 | RT-PCR | 221 (113) | Adult | 55·0 (39·0-66·5) | Severe (n=55) and non-severe (n=166) | NR | |
| 171 | Zhang 2020g  [171] | China  (Zhejiang) | 17 Jan to 8 Feb, 2020 | NAT | 645 (317) | Adult | 40·8±14·0 | Normal imaging findings (n=72) and  abnormal imaging findings (n=573) | NR | |
| 172 | Zhang 2020h  [172] | China  (Hubei) | Until Mar 20, 2020 | NAT | 89 (89) | Adult | 31·9±3·8 | New mothers | NR | |
| 173 | Zhang 2020i  [173] | China  (Hubei) | 18 Feb to 12 Mar, 2020 | NAT | 212 (127) | Adult | 48·5±13·1 | None | NR | |
| 174 | Zhang 2020j  [174] | China  (Hubei) | 13 Jan to 26 Feb, 2020 | RT-PCR | 28 (11) | Adult | 65·0 (56·0-70·0) | Cancer patients | NR | |
| 175 | Zhang 2020k  [175] | China  (Hubei) | 2 Jan to 13 Feb, 2020 | RT-PCR | 319 (171) | Adult | 47·0 (34·0-61·0) | Survived (n=273) and non-survived (n=46) | NR | |
| 176 | Zhang 2020l  [176] | China  (Hubei) | 16 Feb to 25 Mar, 2020 | RT-PCR | 95 (42) | Adult | 49·0 (39·0-58·0) | Severe (n=32) and non-severe (n=63) | NR | |
| 177 | Zhao 2020  [177] | China  (Anhui) | 23 Jan to 5 Feb, 2020 | RT-PCR | 19 (8) | Adult | 48·0 (27·0-56·0) | None | NR | |
| 178 | Zhao 2020a  [178] | China  (Hubei) | 23 Jan to 31 Jan, 2020 | RT-PCR | 37 (23) | Adult | 41·0±19·6 | None | NR | |
| 179 | Zhao 2020b  [179] | China  (Hunan) | 16 Jan to 6 Feb, 2020 | RT-PCR | 56 (30) | Adult | 50·3±15·6 | None | NR | |
| 180 | Zhao 2020c  [180] | China  (Beijing) | 21 Jan to 8 Feb, 2020 | RT-PCR | 77 (43) | Adult (n=73) and paediatric (n=4) | 52·0±20·0 | Severe (n=20) and non-severe  (n=57) | NR | |
| 181 | Zhao 2020d  [181] | China  (Hunan) | 16 Jan to 4 Feb, 2020 | RT-PCR | 118 (58) | Adult (n=115) and paediatric (n=3) | 44·0±13·6 | None | NR | |
| 182 | Zhao 2020e  [182] | China  (Hunan) | NR | RT-PCR | 101 (45) | Adult (n=100) and paediatric (n=1) | 44·4 (17·0-75·0) | None | NR | |
| 183 | Zhao 2020f  [183] | China  (Anhui) | 21 Jan to 16 Feb, 2020 | RT-PCR | 75 (33) | Adult (n=74) and paediatric (n=1) | 47·0 (34·0-55·0) | Hospitalized patients | NR | |
| 184 | Zhao 2020g  [184] | China  (Anhui) | Jan 2020 to Feb, 2020 | RT-PCR | 80 (37) | Adult | 44·0±11·7 | None | 37·4-39·5 | |
| 185 | Zhao 2020h  [185] | China  (Zhejiang) | 27 Jan to 12 Feb, 2020 | RT-PCR | 459 (215) | Adult | 48·0 (2·0-93·0) | Severe (n=42) and non-severe (n=417) | NR | |
| 186 | Zheng 2020  [186] | China  (Hubei) | Feb 15, 2020 | RT-PCR | 55 (31) | Adult | Severe: 62·0 (29·0-91·0) and non-severe: 59·0 (29·0-77·0) | Severe (n=21) and non-severe (n=34) | NR | |
| 187 | Zheng 2020a  [187] | China  (Hubei) | 1 Feb to 10 Feb, 2020 | RT-PCR | 25 (11) | Paediatric | 3·0 (2·0-9·0) | None | NR | |
| 188 | Zhong 2020  [188] | China  (Hubei) | 1 Jan to 14 Feb, 2020 | RT-PCR | 49 (42) | Adult | 31·0 (29·0-34·0) | None | NR | |
| 189 | Zhou 2020  [189] | China  (Hubei) | 23 Jan to 5 Feb, 2020 | RT-PCR | 191(72) | Adult | 56·0 (46·0-67·0) | Survived  (n=137) and non-survived  (n=54) | NR | |
| 190 | Zhou 2020a  [190] | China  (Hubei) | 16 Jan to 30 Jan, 2020 | NAT | 62 (23) | Adult | 52·8±12·2 | Early-stage (n=40) and progressive-stage (n=22) | NR | |
| 191 | Zhou 2020b  [191] | China  (Hubei) | 20 Dec, 2019 to 9 Feb, 2020 | NAT | 254 (139) | Adult | 50·0 (36·0-65·0) | Medical staff (n=93) and  non-medical staff (n=161) | NR | |
| 192 | Zhou 2020c  [192] | China  (Chongqing) | 21 Jan to 4 Feb, 2020 | RT-PCR | 62 (28) | Adult | 47·6±13·4 | Early-stage (n=34) and progressive-stage (n=28) | NR | |
| 193 | Zhou 2020d  [193] | China  (Guangdong) | 20 Jan to 10  Feb, 2020 | RT-PCR | 9 (5) | Paediatric | 0·0-3·0 | None | NR | |
| 194 | Zhu 2020  [194] | China  (Anhui) | 24 Jan to 20 Feb, 2020 | NAT | 32 (17) | Adult | 46·0 (35·0‐52·0) | None | 37·7±0·8 | |
| 195 | Zhu 2020a  [195] | China  (Hubei) | 20 Jan to 15 Feb, 2020 | NR | 114 (47) | Adult | 76·0 (72·0-82·0) | Severe (n=43) and non-severe (n=71) | NR | |
| 196 | Zhu 2020b  [196] | China  (Guangdong) | 24 Jan to 5 Feb, 2020 | RT-PCR | 6 (6) | Adult | 27·0-63·0 | Female only | NR | |
| 197 | Zhu 2020c  [197] | China  (Hubei) | 20 Jan to 5 Feb, 2020 | RT-PCR | 10 (10) | Adult | 30·9±3·3 | New mothers | NR | |
| SD: Standard deviation; IQR: interquartile range; RT-PCR: reverse transcription polymerase chain reaction; ICU: intensive care unit; ARDS: acute respiratory distress syndrome; NAT: nucleic acid test; HDs: haematological disorders; GI: gastrointestinal; HBV: hepatitis B virus; NR: not reported | | | | | | | | | |  |

**References**

1. Ai J, Chen J, Wang Y, Liu X, Fan W, Qu G, et al. The cross-sectional study of hospitalized coronavirus disease 2019 patients in Xiangyang, Hubei province. medRxiv. 2020; doi: 10.1101/2020.02.19.20025023

2. Bhatraju PK, Ghassemieh BJ, Nichols M, Kim R, Jerome KR, Nalla AK, et al. Covid-19 in critically ill patients in the Seattle region-case series. N Engl J Med. 2020; doi: 10.1056/NEJMoa2004500

3. Bian H, Zheng Z-H, Wei D, Zhang Z, Kang W-Z, Hao C-Q, et al. Meplazumab treats COVID-19 pneumonia: an open-labelled, concurrent controlled add-on clinical trial. medRxiv. 2020; doi: 10.1101/2020.03.21.20040691

4. Cao B, Wang Y, Wen D, Liu W, Wang J, Fan G, et al. A trial of lopinavir–ritonavir in adults hospitalized with severe Covid-19. N Engl J Med. 2020; doi: 10.1056/NEJMoa2001282

5. Cao M, Zhang D, Wang Y, Lu Y, Zhu X, Li Y, et al. Clinical features of patients infected with the 2019 novel coronavirus (COVID-19) in Shanghai, China. medRxiv. 2020; doi: 10.1101/2020.03.04.20030395

6. Cao W. Clinical features and laboratory inspection of novel coronavirus pneumonia (COVID-19) in Xiangyang, Hubei. medRxiv. 2020; doi: 10.1101/2020.02.23.20026963

7. Chang D, Lin M, Wei L, Xie L, Zhu G, Cruz CSD, et al. Epidemiologic and clinical characteristics of novel coronavirus infections involving 13 patients outside Wuhan, China. JAMA. 2020; 323(11):1092-3. doi: 10.1001/jama.2020.1623

8. Chang D, Mo G, Yuan X, Tao Y, Peng X, Wang F, et al. Time kinetics of viral clearance and resolution of symptoms in Novel coronavirus infection. Am J Respir Crit Care Med. 2020; doi: 10.1164/rccm.202003-0524LE

9. Chen L, Zhao J, Peng J, Li X, Deng X, Geng Z, et al. Detection of 2019-nCoV in Saliva and Characterization of Oral Symptoms in COVID-19 Patients. SSRN. 2020; doi: 10.2139/ssrn.3557140

10. Chen G, Wu D, Guo W, Cao Y, Huang D, Wang H, et al. Clinical and immunologic features in severe and moderate forms of Coronavirus Disease 2019. medRxiv. 2020; doi: 10.1101/2020.02.16.20023903

11. Chen J, Qi T, Liu L, Ling Y, Qian Z, Li T, et al. Clinical progression of patients with COVID-19 in Shanghai, China. J Infect. 2020; 80(5):1-6. doi: 10.1016/j.jinf.2020.03.004

12. Chen H, Guo J, Wang C, Luo F, Yu X, Zhang W, et al. Clinical characteristics and intrauterine vertical transmission potential of COVID-19 infection in nine pregnant women: a retrospective review of medical records. Lancet. 2020; 395(10226):809-15. doi: 10.1016/S0140-6736(20)30360-3

13. Chen Z, Hu J, Zhang Z, Jiang S, Wang T, Shi Z, et al. Caution: The clinical characteristics of COVID-19 patients at admission are changing. medRxiv. 2020; doi: 10.1101/2020.03.03.20030833

14. Chen L, Deng C, Chen X, Zhang X, Chen B, Yu H, et al. Ocular manifestations and clinical characteristics of 534 cases of COVID-19 in China: A cross-sectional study. medRxiv. 2020; doi: 10.1101/2020.03.12.20034678

15. Chen N, Zhou M, Dong X, Qu J, Gong F, Han Y, et al. Epidemiological and clinical characteristics of 99 cases of 2019 novel coronavirus pneumonia in Wuhan, China: a descriptive study. Lancet. 2020; 395(10223):507-13. doi: 10.1016/S0140-6736(20)30211-7

16. Chen R, Zhang Y, Huang L, Cheng B-h, Xia Z-y, Meng Q-t. Safety and efficacy of different anesthetic regimens for parturients with COVID-19 undergoing Cesarean delivery: a case series of 17 patients. Can J Anesth. 2020; doi: 10.1007/s12630-020-01630-7

17. Chen X, Zheng F, Qing Y, Ding S, Yang D, Lei C, et al. Epidemiological and clinical features of 291 cases with coronavirus disease 2019 in areas adjacent to Hubei, China: a double-center observational study. medRxiv. 2020; doi: 10.1101/2020.03.03.20030353

18. Chen Y, Zhang K, Zhu G, Liu L, Yan X, Cai Z, et al. Clinical Characteristics and Current Treatment of Critically Ill Patients with COVID-19 Outside Wuhan, China: A Multicenter, Retrospective, Observational Study. SSRN. 2020; doi: 10.2139/ssrn.3551426

19. Chen X, Yuan W, Shao Z, Liu G, Wang W, Zhang S, et al. The Desynchrony between Clinical Course and RT-PCR Test Results in Patients with COVID-19 Infected Pneumonia During the Treatment in Wuhan, China. SSRN. 2020; doi: 10.2139/ssrn.3551404

20. Chen X, Jiang Q, Ma Z, Ling J, Hu W, Cao Q, et al. Clinical Characteristics of Hospitalized Patients with SARS-CoV-2 and Hepatitis B virus Co-infection. medRxiv. 2020; doi: 10.1101/2020.03.23.20040733

21. Chen T, Wu D, Chen H, Yan W, Yang D, Chen G, et al. Clinical characteristics of 113 deceased patients with coronavirus disease 2019: retrospective study. BMJ. 2020; 368(doi: 10.1136/bmj.m1091

22. Chen S, Liao E, Shao Y. Clinical analysis of pregnant women with 2019 novel coronavirus pneumonia. J Med Virol. 2020; doi: 10.1002/jmv.25789

23. Chen C, Huang J, Cheng Z, Wu J, Chen S, Zhang Y, et al. Favipiravir versus Arbidol for COVID-19: a randomized clinical trial. medRxiv. 2020; doi: 10.1101/2020.03.17.20037432

24. Cheng Y, Luo R, Wang K, Zhang M, Wang Z, Dong L, et al. Kidney disease is associated with in-hospital death of patients with COVID-19. Kidney Int. 2020; 97 (5):829-38. doi: 10.1016/j.kint.2020.03.005

25. Cheng Z, Lu Y, Cao Q, Qin L, Pan Z, Yan F, et al. Clinical features and chest CT manifestations of coronavirus disease 2019 (COVID-19) in a single-center study in Shanghai, China. Am J Roentgenol. 2020; doi: 10.2214/AJR.20.22959

26. Cheng B, Jiang T, Zhang L, Hu R, Tian J, Jiang Y, et al. Clinical Characteristics of Pregnant Women with Coronavirus Disease 2019 in Wuhan, China. SSRN. 2020; doi: 10.2139/ssrn.3555240

27. Chu J, Yang N, Wei Y, Yue H, Zhang F, Zhao J, et al. Clinical Characteristics of 54 medical staff with COVID‐19: A retrospective study in a single center in Wuhan, China. J Med Virol. 2020; doi: 10.1002/jmv.25793

28. Liu C, Jiang Z, Shao C, Zhang H, Yue H, Chen Z, et al. Preliminary study of the relationship between novel coronavirus pneumonia and liver function damage: a multicenter study. Zhonghua Gan Zang Bing Za Zhi. 2020; 28(2):148-52. doi: 10.3760/cma.j.issn.1007-3418.2020.02.003

29. Chung M, Bernheim A, Mei X, Zhang N, Huang M, Zeng X, et al. CT imaging features of 2019 novel coronavirus (2019-nCoV). Radiology. 2020; 295(1):202-7. doi: 10.1148/radiol.2020200230

30. Cui P, Chen Z, Wang T, Dai J, Zhang J, Ding T, et al. Clinical features and sexual transmission potential of SARS-CoV-2 infected female patients: a descriptive study in Wuhan, China. medRxiv. 2020; doi: 10.1101/2020.02.26.20028225

31. Deng Y, Liu W, Liu K, Fang Y-Y, Shang J, Wang K, et al. Clinical characteristics of fatal and recovered cases of coronavirus disease 2019 (COVID-19) in Wuhan, China: a retrospective study. Chin Med J (Engl). 2020; doi: 10.1097/CM9.0000000000000824

32. Diao K, Han P, Pang T, Li Y, Yang Z. HRCT imaging features in representative imported cases of 2019 novel coronavirus pneumonia. Precis Clin Med. 2020; 3(1):9-13. doi: 10.1093/pcmedi/pbaa004

33. Ding Q, Lu P, Fan Y, Xia Y, Liu M. The clinical characteristics of pneumonia patients co‐infected with 2019 novel coronavirus and influenza virus in Wuhan, China. J Med Virol. 2020; doi: 10.1002/jmv.25781

34. Du R, Liang L, Yang C, Li M, Guo G, van Halm-Lutterodt N, et al. Patient Predisposition at Hospital Admission Indirectly Dictates Disease Severity, Clinical Course and Outcomes of COVID-19 Pneumonia Patients in Wuhan, China. SSRN. 2020; doi: 10.2139/ssrn.3543584

35. Du W, Yu J, Wang H, Zhang X, Zhang S, Li Q, et al. Clinical characteristics of COVID-19 in children compared with adults in Shandong Province, China. Infection. 2020; doi: 10.1007/s15010-020-01427-2

36. Easom N, Moss P, Barlow G, Samson A, Taynton T, Adams K, et al. Sixty-eight consecutive patients assessed for COVID-19 infection: Experience from a UK Regional infectious diseases Unit. Influenza Other Respi Viruses. 2020; doi: 10.1111/irv.12739

37. Fan H, Zhang L, Huang B, Zhu M, Zhou Y, Zhang H, et al. Retrospective Analysis of Clinical Features in 101 Death Cases with COVID-19. medRxiv. 2020; doi: 10.1101/2020.03.09.20033068

38. Fan Z, Chen L, Li J, Cheng X, Yang J, Tian C, et al. Clinical Features of COVID-19-Related Liver Damage. Clin Gastroenterol Hepatol. 2020; doi: 10.1016/j.cgh.2020.04.002

39. Feng Z, Yu Q, Yao S, Luo L, Duan J, Yan Z, et al. Early prediction of disease progression in 2019 novel coronavirus pneumonia patients outside Wuhan with CT and clinical characteristics. medRxiv. 2020; doi: 10.1101/2020.02.19.20025296

40. Feng K, Yun Y, Wang X, Yang G, Zheng Y, Lin C, et al. Analysis of CT features of 15 children with 2019 novel coronavirus infection. Zhonghua Er Ke Za Zhi. 2020; doi: 10.3760/cma.j.issn.0578-1310.2020.0007

41. Fu H, Xu H, Zhang N, Xu H, Li Z, Chen H, et al. Association between Clinical, Laboratory and CT Characteristics and RT-PCR Results in the Follow-up of COVID-19 patients. medRxiv. 2020; doi: 10.1101/2020.03.19.20038315

42. Guan W-j, Ni Z-y, Hu Y, Liang W-h, Ou C-q, He J-x, et al. Clinical characteristics of coronavirus disease 2019 in China. N Engl J Med. 2020; doi: 10.1056/NEJMoa2002032

43. Guan W-j, Liang W-h, Zhao Y, Liang H-r, Chen Z-s, Li Y-m, et al. Comorbidity and its impact on 1590 patients with Covid-19 in China: A Nationwide Analysis. Eur Respir J. 2020; doi: 10.1183/13993003.00547-2020

44. Han R, Huang L, Jiang H, Dong J, Peng H, Zhang D. Early clinical and CT manifestations of coronavirus disease 2019 (COVID-19) pneumonia. Am J Roentgenol. 2020; doi: 10.2214/AJR.20.22961

45. Han X, Cao Y, Jiang N, Chen Y, Alwalid O, Zhang X, et al. Novel Coronavirus Pneumonia (COVID-19) Progression Course in 17 Discharged Patients: Comparison of Clinical and Thin-Section CT Features During Recovery. Clin Infect Dis. 2020; doi: 10.1093/cid/ciaa271

46. He W, Chen L, Chen L, Yuan G, Fang Y, Chen W, et al. Clinical Characteristics and Outcome of 31 Cases with Covid-19 Diagnosed in Haematology Units in Wuhan, China: A Retrospective Cohort Study. SSRN. 2020; doi: 10.2139/ssrn.3558004

47. Hou J, Wan X, Shen Q, Leng Y, Xia Z, Zhao B, et al. Epidemiologic and Clinical Characteristics of Surgical Patients Infected with COVID-19 in Wuhan. SSRN. 2020; doi: 10.2139/ssrn.3550044

48. Hu Z, Song C, Xu C, Jin G, Chen Y, Xu X, et al. Clinical characteristics of 24 asymptomatic infections with COVID-19 screened among close contacts in Nanjing, China. Sci China Life Sci. 2020; doi: 10.1007/s11427-020-1661-4

49. Hu X, Zeng W, Zhang Y, Zhen Z, Cheng L, Wang X, et al. CT Imaging Features of Different Clinical Types of COVID-19: A Chinese Multicenter Study. SSRN. 2020; doi: 10.2139/ssrn.3550043

50. Hu L, Chen S, Fu Y, Gao Z, Long H, Ren H-w, et al. Risk factors associated with clinical outcomes in 323 COVID-19 patients in Wuhan, China. Clin Infect Dis. 2020; doi: 10.1093/cid/ciaa539

51. Huang C, Wang Y, Li X, Ren L, Zhao J, Hu Y, et al. Clinical features of patients infected with 2019 novel coronavirus in Wuhan, China. Lancet. 2020; 395(10223):497-506. doi: 10.1016/S0140-6736(20)30183-5

52. Huang M, Zhan F, Wang J, Yi Q, Zhu F, Yang H, et al. Epidemiological and Clinical Features of 197 Patients Infected with 2019 Novel Coronavirus in Chongqing, China: A Single Center Descriptive Study. SSRN. 2020; doi: 10.2139/ssrn.3539687

53. Huang R, Zhu L, Xue L, Liu L, Yan X, Wang J, et al. Clinical Findings of Patients with Coronavirus Disease 2019 in Jiangsu Province, China: A Retrospective, Multi-Center Study. PLoS Negl Trop Dis. 2020; 14(5):e0008280. doi: 10.1371/journal.pntd.0008280

54. Huang Y, Zhou H, Yang R, Xu Y, Feng X, Gong P. Clinical characteristics of 36 non-survivors with COVID-19 in Wuhan, China. medRxiv. 2020; doi: 10.1101/2020.02.27.20029009

55. Huang Y, Tu M, Wang S, Chen S, Zhou W, Chen D, et al. Clinical characteristics of laboratory confirmed positive cases of SARS-CoV-2 infection in Wuhan, China: a retrospective single center analysis. Travel Med Infect Dis. 2020; doi: 10.1016/j.tmaid.2020.101606

56. Ji W, Bishnu G, Cai Z, Shen X. Analysis clinical features of COVID-19 infection in secondary epidemic area and report potential biomarkers in evaluation. medRxiv. 2020; doi: 10.1101/2020.03.10.20033613

57. Cai J, Xu J, Lin D, Xu L, Qu Z, Zhang Y, et al. A Case Series of children with 2019 novel coronavirus infection: clinical and epidemiological features. Clin Infect Dis. 2020; doi: 10.1093/cid/ciaa198/5766430

58. Jin X, Lian J-S, Hu J-H, Gao J, Zheng L, Zhang Y-M, et al. Epidemiological, clinical and virological characteristics of 74 cases of coronavirus-infected disease 2019 (COVID-19) with gastrointestinal symptoms. Gut. 2020; doi: 10.1136/gutjnl-2020-320926

59. Chen L, Liu H, Liu W, Liu J, Liu K, Shang J. Analysis of clinical characteristics of 29 cases of new coronavirus pneumonia in 2019. Zhonghua Jie He He Hu Xi Za Zhi. 2020; doi: 10.3760/cma.j.issn.1001-0939.2020.0005

60. Lei Y. Clinical features of imported cases of coronavirus disease 2019 in Tibetan patients in the Plateau area. medRxiv. 2020; doi: 10.1101/2020.03.09.20033126

61. Lei Z-Y, Cao H-J, Jie Y-S, Huang Z-L, Guo X-Y, Chen J-F, et al. Comparison of Epidemiological and Clinical Features of Patients with Coronavirus Disease (COVID-19) in Wuhan and Outside Wuhan, China. SSRN. 2020; doi: 10.2139/ssrn.3546082

62. Lescure F-X, Bouadma L, Nguyen D, Parisey M, Wicky P-H, Behillil S, et al. Clinical and virological data of the first cases of COVID-19 in Europe: a case series. Lancet Infect Dis. 2020; doi: 10.1016/S1473-3099(20)30200-0

63. Li J, Li S, Cai Y, Liu Q, Li X, Zeng Z, et al. Epidemiological and Clinical Characteristics of 17 Hospitalized Patients with 2019 Novel Coronavirus Infections Outside Wuhan, China. medRxiv. 2020; doi: 10.1101/2020.02.11.20022053

64. Li J, Long X, Luo H, Fang F, Lv X, Zhang D, et al. Clinical Characteristics of Deceased Patients Infected with SARS-CoV-2 in Wuhan, China. SSRN. 2020; doi: 10.2139/ssrn.3546043

65. Li J, Zhang Y, Wang F, Liu B, Li H, Tang G, et al. Sex differences in clinical findings among patients with coronavirus disease 2019 (COVID-19) and severe condition. medRxiv. 2020; doi: 10.1101/2020.02.27.20027524

66. Li K, Wu J, Wu F, Guo D, Chen L, Fang Z, et al. The Clinical and Chest CT Features Associated with Severe and Critical COVID-19 Pneumonia. Investig Radiol. 2020; doi: 10.1097/RLI.0000000000000672

67. Li Y, Wang Z, Hui Y, Tong X, Mao X, Huang L, et al. Clinical Characteristics of 77 Novel Coronavirus 2019 Infected Patients with Respiratory Failure in the Terminal Stage in Wuhan. Int J Infect Dis. 2020; 96(266–9. doi: 10.1016/j.ijid.2020.05.008

68. Li Y, Wang J, Wang C, Yang Q, Xu Y, Xu J, et al. Characteristics of Respiratory Virus Infection During the Outbreak of 2019 Novel Coronavirus in Beijing. SSRN. 2020; doi: 10.2139/ssrn.3548768

69. Li K, Fang Y, Li W, Pan C, Qin P, Zhong Y, et al. CT image visual quantitative evaluation and clinical classification of coronavirus disease (COVID-19). Eur Radiol. 2020; doi: 10.1007/s00330-020-06817-6

70. Lian J, Jin X, Hao S, Cai H, Zhang S, Zheng L, et al. Analysis of Epidemiological and Clinical features in older patients with Corona Virus Disease 2019 (COVID-19) out of Wuhan. Clin Infect Dis. 2020; doi: 10.1093/cid/ciaa242/5811557

71. Liang Y, Liang J, Zhou Q, Li X, Lin F, Deng Z, et al. Prevalence and clinical features of 2019 novel coronavirus disease (COVID-19) in the Fever Clinic of a teaching hospital in Beijing: a single-center, retrospective study. medRxiv. 2020; doi: 10.1101/2020.02.25.20027763

72. Zhang J, Guo L, Liu H, Lv J, Xu L. Epidemiological and clinical characteristics of COVID-19 in adolescents and young adults. Cell. 2020; doi: 10.1016/j.xinn.2020.04.001

73. Liu K, Chen Y, Wu D, Lin R, Wang Z, Pan L. Effects of progressive muscle relaxation on anxiety and sleep quality in patients with COVID-19. Complement Ther Clin Pract. 2020; doi: 10.1016/j.ctcp.2020.101132

74. Jian-ya G. Clinical characteristics of 51 patients discharged from hospital with COVID-19 in Chongqing, China. medRxiv. 2020; doi: 10.1101/2020.02.20.20025536

75. Li J, Song H, Hu Z. Clinical features and outcomes of 2019 novel coronavirus-infected patients with cardiac injury. medRxiv. 2020; doi: 10.1101/2020.03.11.20030957

76. Liu D, Li L, Wu X, Zheng D, Wang J, Yang L, et al. Pregnancy and perinatal outcomes of women with coronavirus disease (COVID-19) pneumonia: a preliminary analysis. Am J Roentgenol. 2020; doi: 10.2214/AJR.20.23072

77. Liu F, Xu A, Zhang Y, Xuan W, Yan T, Pan K, et al. Patients of COVID-19 may benefit from sustained lopinavir-combined regimen and the increase of eosinophil may predict the outcome of COVID-19 progression. Int J Infect Dis. 2020; doi: 10.1016/j.ijid.2020.03.013

78. Liu H, Liu F, Li J, Zhang T, Wang D, Lan W. Clinical and CT imaging features of the COVID-19 pneumonia: Focus on pregnant women and children. J Infect. 2020; doi: 10.1016/j.jinf.2020.03.007

79. Liu J, Li S, Liu J, Liang B, Wang X, Wang H, et al. Longitudinal characteristics of lymphocyte responses and cytokine profiles in the peripheral blood of SARS-CoV-2 infected patients. EBioMedicine. 2020; 102763. doi: 10.1016/j.ebiom.2020.102763

80. Liu J, Ouyang L, Guo P, sheng Wu H, Fu P, liang Chen Y, et al. Epidemiological, Clinical Characteristics and Outcome of Medical Staff Infected with COVID-19 in Wuhan, China: A Retrospective Case Series Analysis. medRxiv. 2020; doi: 10.1101/2020.03.09.20033118

81. Liu K, Fang Y-Y, Deng Y, Liu W, Wang M-F, Ma J-P, et al. Clinical characteristics of novel coronavirus cases in tertiary hospitals in Hubei Province. Chin Med J (Engl). 2020; doi: 10.1097/CM9.0000000000000744

82. Liu K-C, Xu P, Lv W-F, Qiu X-H, Yao J-L, Jin-Feng G. CT manifestations of coronavirus disease-2019: a retrospective analysis of 73 cases by disease severity. Eur J Radiol. 2020; 126(1-5. doi: 10.1016/j.ejrad.2020.108941

83. Liu Y, Chen H, Tang K, Guo Y. Clinical manifestations and outcome of SARS-CoV-2 infection during pregnancy. J Infect. 2020; doi: 10.1016/j.jinf.2020.02.028

84. Liu Y, Sun W, Chen L, Wang Y, Zhang L, Yu L. Clinical Characteristics and Progression of 2019 Novel Coronavirus-Infected Patients Concurrent Acute Respiratory Distress Syndrome. medRxiv. 2020; doi: 10.1101/2020.02.17.20024166

85. Liu W, Tao Z-W, Wang L, Yuan M-L, Liu K, Zhou L, et al. Analysis of factors associated with disease outcomes in hospitalized patients with 2019 novel coronavirus disease. Chin Med J (Engl). 2020; doi: 10.1097/CM9.0000000000000775

86. Liu Y, Yang Y, Zhang C, Huang F, Wang F, Yuan J, et al. Clinical and biochemical indexes from 2019-nCoV infected patients linked to viral loads and lung injury. Sci China Life Sci. 2020; 63(3):364-74. doi: 10.1007/s11427-020-1643-8

87. Liu YSL, Zhang D, Tang S, Chen H, Chen L, He X, et al. The Epidemiological and Clinical Characteristics of 2019 Novel Coronavirus Infection in Changsha, China. SSRN. 2020; doi: 10.2139/ssrn.3537093

88. Liu S, Luo H, Wang Y, Wang D, Ju S, Yang Y. Characteristics and Associations with Severity in COVID-19 Patients: A Multicentre Cohort Study from Jiangsu Province, China. SSRN. 2020; doi: 10.2139/ssrn.3548753

89. Liu K, Chen Y, Lin R, Han K. Clinical features of COVID-19 in elderly patients: A comparison with young and middle-aged patients. J Infect. 2020; doi: 10.1016/j.jinf.2020.03.005

90. Lo IL, Lio CF, Cheong HH, Lei CI, Cheong TH, Zhong X, et al. Evaluation of SARS-CoV-2 RNA shedding in clinical specimens and clinical characteristics of 10 patients with COVID-19 in Macau. Int J Biol Sci. 2020; 16(10):1698-707. doi: 10.7150/ijbs.45357

91. Chen M, Fan Y, Wu X, Zhang L, Guo T, Deng K, et al. Clinical characteristics and risk factors for fatal outcome in patients with 2019-coronavirus infected disease (COVID-19) in Wuhan, China. SSRN. 2020; doi: 10.2139/ssrn.3546069

92. Lu H, Ai J, Shen Y, Li Y, Li T, Zhou X, et al. A descriptive study of the impact of diseases control and prevention on the epidemics dynamics and clinical features of SARS-CoV-2 outbreak in Shanghai, lessons learned for metropolis epidemics prevention. medRxiv. 2020; doi: 10.1101/2020.02.19.20025031

93. Lu C, Yang W, Hu Y, Hui J, Zhou G, Shu J, et al. Coronavirus Disease 2019 (COVID-19) Pneumonia: Early Stage Chest CT Imaging Features and Clinical Relevance. SSRN. 2020; doi: 10.2139/ssrn.3543606

94. Luo XM, Zhou W, Xia H, Yang W, Yan X, Wang B, et al. Characteristics of SARS-CoV-2 Infected Patients with Clinical Outcome During Epidemic Ongoing Outbreak in Wuhan, China. SSRN. 2020; doi: 10.2139/ssrn.3552812

95. Mao B, Liu Y, Chai Y-h, Jin X-y, Luo HW, Yang J-w, et al. Early Discern COVID-19 from the Suspected Patients via Fever Clinics: A Multicenter Cohort Study from Shanghai. SSRN. 2020; doi: 10.2139/ssrn.3550037

96. Miao C, Zhuang J, Jin M, Xiong H, Huang P, Zhao Q, et al. A comparative multi-centre study on the clinical and imaging features of comfirmed and uncomfirmed patients with COVID-19. medRxiv. 2020; doi: 10.1101/2020.03.22.20040782

97. Liu M, He P, Liu H, Wang X, Li F, Chen S, et al. Clinical characteristics of 30 medical workers infected with new coronavirus pneumonia. Zhonghua Jie He He Hu Xi Za Zhi. 2020; 43(1-7. doi: 10.3760/cma.j.issn.1001-0939.2020.0016

98. Mo P, Xing Y, Xiao Y, Deng L, Zhao Q, Wang H, et al. Clinical characteristics of refractory COVID-19 pneumonia in Wuhan, China. Clin Infect Dis. 2020; doi: 10.1093/cid/ciaa270/5805508

99. Nie R, Wang S-s, Yang Q, Fan C-f, Liu Y-l, He W-c, et al. Clinical features and the maternal and neonatal outcomes of pregnant women with coronavirus disease 2019. medRxiv. 2020; doi: 10.1101/2020.03.22.20041061

100. Pan F, Ye T, Sun P, Gui S, Liang B, Li L, et al. Time course of lung changes on chest CT during recovery from 2019 novel coronavirus (COVID-19) pneumonia. Radiology. 2020; doi: 10.1148/radiol.2020200370

101. Pung R, Chiew CJ, Young BE, Chin S, Chen MI, Clapham HE, et al. Investigation of three clusters of COVID-19 in Singapore: implications for surveillance and response measures. Lancet. 2020; 395(10229):1039-46. doi: 10.1016/S0140-6736(20)30528-6

102. Qi D, Yan X, Tang X, Peng J, Yu Q, Feng L, et al. Epidemiological and clinical features of 2019-nCoV acute respiratory disease cases in Chongqing municipality, China: a retrospective, descriptive, multiple-center study. medRxiv. 2020; doi: 10.1101/2020.03.01.20029397

103. Qian G-Q, Yang N-B, Ding F, Ma AHY, Wang Z-Y, Shen Y-F, et al. Epidemiologic and Clinical Characteristics of 91 Hospitalized Patients with COVID-19 in Zhejiang, China: A retrospective, multi-centre case series. QJM. 2020; doi: 10.1093/qjmed/hcaa089

104. Qin C, Zhou L, Hu Z, Zhang S, Yang S, Tao Y, et al. Dysregulation of immune response in patients with COVID-19 in Wuhan, China. Clin Infect Dis. 2020; doi: 10.1093/cid/ciaa248/5803306

105. Qin X, Qiu S, Yuan Y, Zong Y, Tuo Z, Li J, et al. Clinical characteristics and treatment of patients infected with COVID-19 in Shishou, China. SSRN. 2020; doi: 10.2139/ssrn.3541147

106. Qiu H, Wu J, Hong L, Luo Y, Song Q, Chen D. Clinical and epidemiological features of 36 children with coronavirus disease 2019 (COVID-19) in Zhejiang, China: an observational cohort study. Lancet Infect Dis. 2020; doi: 10.1016/S1473-3099(20)30198-5

107. Qiu C, Xiao Q, Liao X, Deng Z, Liu H, Shu Y, et al. Transmission and clinical characteristics of coronavirus disease 2019 in 104 outside-Wuhan patients, China. J Med Virol. 2020; doi: 10.1002/jmv.25975

108. Shi H, Han X, Jiang N, Cao Y, Alwalid O, Gu J, et al. Radiological findings from 81 patients with COVID-19 pneumonia in Wuhan, China: a descriptive study. Lancet Infect Dis. 2020; doi: 10.1016/S1473-3099(20)30086-4

109. Shi Q, Zhao K, Yu J, Feng J, Zhao K, Zhang X, et al. Clinical characteristics of 101 non-surviving hospitalized patients with COVID-19: A single center, retrospective study. medRxiv. 2020; doi: 10.1101/2020.03.04.20031039

110. Shu L, Wang X, Li M, Chen X, Shi L, Wu M, et al. Clinical Characteristics of 545 Cases Confirmed COVID-19 in Wuhan Stadium Cabin Hospital. SSRN. 2020; doi: 10.2139/ssrn.3552844

111. Song F, Shi N, Shan F, Zhang Z, Shen J, Lu H, et al. Emerging 2019 novel coronavirus (2019-nCoV) pneumonia. Radiology. 2020; 295(1):210-7. doi: 10.1148/radiol.2020200274

112. Su L, Ma X, Yu H, Zhang Z, Bian P, Han Y, et al. The different clinical characteristics of corona virus disease cases between children and their families in China–the character of children with COVID-19. Emerg Microbes Infect. 2020; 9(1):707-13. doi: 10.1080/22221751.2020.1744483

113. Sun D, Li H, Lu X-X, Xiao H, Ren J, Zhang F-R, et al. Clinical features of severe pediatric patients with coronavirus disease 2019 in Wuhan: a single center’s observational study. World J Pediatr. 2020; doi: 10.1007/s12519-020-00354-4

114. Tang A, Xu W, Chen P, Li G, Liu Y, Liu L. A retrospective study of the clinical characteristics of COVID-19 infection in 26 children. medRxiv. 2020; doi: 10.1101/2020.03.08.20029710

115. Tang X, Du R, Wang R, Cao T, Guan L, Yang C, et al. Comparison of hospitalized patients with acute respiratory distress syndrome caused by COVID-19 and H1N1. Chest. 2020; doi: 10.1016/j.chest.2020.03.032

116. Tian S, Hu N, Lou J, Chen K, Kang X, Xiang Z, et al. Characteristics of COVID-19 infection in Beijing. J Infect. 2020; doi: 10.1016/j.jinf.2020.02.018

117. Tian S, Chang Z, Wang Y, Wu M, Zhang W, Zhou G, et al. Clinical characteristics and reasons of different duration from onset to release from quarantine for patients with COVID-19 Outside Hubei province, China. medRxiv. 2020; doi: 10.1101/2020.03.21.20038778

118. To KK-W, Tsang OT-Y, Leung W-S, Tam AR, Wu T-C, Lung DC, et al. Temporal profiles of viral load in posterior oropharyngeal saliva samples and serum antibody responses during infection by SARS-CoV-2: an observational cohort study. Lancet Infect Dis. 2020; doi: 10.1016/S1473-3099(20)30196-1

119. Wan S, Xiang Y, Fang W, Zheng Y, Li B, Hu Y, et al. Clinical Features and Treatment of COVID‐19 Patients in Northeast Chongqing. J Med Virol. 2020; doi: 10.1002/jmv.25783

120. Wang K, Kang S, Tian R, Zhang X, Wang Y. Imaging manifestations and diagnostic value of chest CT of coronavirus disease 2019 (COVID-19) in the Xiaogan area. Clin Radiol. 2020; doi: 10.1016/j.crad.2020.03.004

121. Wang D, Hu B, Hu C, Zhu F, Liu X, Zhang J, et al. Clinical characteristics of 138 hospitalized patients with 2019 novel coronavirus–infected pneumonia in Wuhan, China. JAMA. 2020; 323(11):1061-9. doi: 10.1001/jama.2020.1585

122. Wang D, Ju X, Xie F, Lu Y, Li F, Huang H, et al. Clinical analysis of 31 cases of 2019 novel coronavirus infection in children from six provinces (autonomous region) of northern China. Zhonghua Er Ke Za Zhi. 2020; 58(4):1-8. doi: 10.3760/cma.j.cn112140-20200225-00138

123. Wang L, Gao Y-h, Zhang G-J. The clinical dynamics of 18 cases of COVID-19 outside of Wuhan, China. Eur Respir J. 2020; doi: 10.1183/13993003.00398-2020

124. Wang W, He J, Wu S. The definition and risks of cytokine release syndrome-like in 11 COVID-19-infected pneumonia critically ill patients: disease characteristics and retrospective analysis. medRxiv. 2020; doi: 10.1101/2020.02.26.20026989

125. Wang Y, Dong C, Hu Y, Li C, Ren Q, Zhang X, et al. Temporal changes of CT findings in 90 patients with COVID-19 pneumonia: a longitudinal study. Radiology. 2020; doi: 10.1148/radiol.2020200843

126. Wang Y, Zhou Y, Yang Z, Xia D, Geng S. Clinical characteristics of patients with severe pneumonia caused by the 2019 novel coronavirus in Wuhan, China. medRxiv. 2020; doi: 10.1101/2020.03.02.20029306

127. Wang Z, Yang B, Li Q, Wen L, Zhang R. Clinical features of 69 cases with coronavirus disease 2019 in Wuhan, China. Clin Infect Dis. 2020; doi: 10.1093/cid/ciaa272/5807944

128. Wang S, Chen Z, Lin Y, Lin L, Lin Q, Fang S, et al. Epidemical and Clinical Characteristics of 165 Patients Infected with SARS-CoV-2 in Fujian Province, China. SSRN. 2020; doi: 10.2139/ssrn.3551402

129. Wang L, He W, Yu X, Hu D, Bao M, Liu H, et al. Coronavirus Disease 2019 in elderly patients: characteristics and prognostic factors based on 4-week follow-up. J Infect. 2020; doi: 10.1016/j.jinf.2020.03.019

130. Wang F, Nie J, Wang H, Zhao Q, Xiong Y, Deng L, et al. Characteristics of peripheral lymphocyte subset alteration in COVID-19 pneumonia. J Infect Dis. 2020; doi: 10.1093/infdis/jiaa150/5813618

131. Wei L, Jin D, Zhang J, Wang B, Sun M, Li X, et al. Clinical Findings of 100 Mild Cases of COVID-19 in Wuhan: A Descriptive Study. SSRN. 2020; doi: 10.2139/ssrn.3551332

132. Wei X-S, Wang X, Niu Y-R, Ye L-L, Peng W-B, Wang Z-H, et al. Clinical Characteristics of SARS-CoV-2 Infected Pneumonia with Diarrhea. SSRN. 2020; doi: 10.2139/ssrn.3546120

133. Wen Y, Wei L, Li Y, Tang X, Feng S, Leung K, et al. Epidemiological and clinical characteristics of COVID-19 in Shenzhen, the largest migrant city of China. medRxiv. 2020; doi: 10.1101/2020.03.22.20035246

134. Wu J, Liu J, Zhao X, Liu C, Wang W, Wang D, et al. Clinical Characteristics of Imported Cases of COVID-19 in Jiangsu Province: A Multicenter Descriptive Study. Clin Infect Dis. 2020; doi: 10.1093/cid/ciaa199/5766408

135. Wu J, Li W, Shi X, Chen Z, Jiang B, Liu J, et al. Early antiviral treatment contributes to alleviate the severity and improve the prognosis of patients with novel coronavirus disease (COVID‐19). J Intern Med. 2020; doi: 10.1111/joim.13063

136. Wu B, Lei Z-Y, Wu K-L, He J-R, Cao H-J, Fu J, et al. Epidemiological and Clinical Features of Imported and Local Patients with Coronavirus Disease 2019 (COVID-19) in Hainan, China. SSRN. 2020; doi: 10.2139/ssrn.3555222

137. Wu Q, Xing Y, Shi L, Li W, Gao Y, Pan S, et al. Epidemiological and Clinical Characteristics of Children with Coronavirus Disease 2019. medRxiv. 2020; doi: 10.1101/2020.03.19.20027078

138. Xia W, Shao J, Guo Y, Peng X, Li Z, Hu D. Clinical and CT features in pediatric patients with COVID‐19 infection: Different points from adults. Pediatr Pulmonol. 2020; doi: 10.1002/ppul.24718

139. Xie C, Jiang L, Huang G, Pu H, Gong B, Lin H, et al. Comparison of different samples for 2019 novel coronavirus detection by nucleic acid amplification tests. Int J Infect Dis. 2020; doi: 10.1016/j.ijid.2020.02.050

140. Xiong Y, Sun D, Liu Y, Fan Y, Zhao L, Li X, et al. Clinical and high-resolution CT features of the COVID-19 infection: comparison of the initial and follow-up changes. Investig Radiol. 2020; doi: 10.1097/RLI.0000000000000674

141. Xu H, Huang S, Liu S, Deng J, Jiao B, Ai L, et al. Evaluation of the clinical characteristics of suspected or confirmed cases of COVID-19 during home care with isolation: A new retrospective analysis based on O2O. medRxiv. 2020; doi: 10.1101/2020.02.26.20028084

142. Xu W, Qu S, Xing M, Zhang M, Lu G, Liao Z, et al. Epidemiologic Features and Clinical Findings of COVID-19-Infected Patients in Suzhou. SSRN. 2020; doi: 10.2139/ssrn.3551352

143. Xu T, Chen C, Zhu Z, Cui M, Chen C, Dai H, et al. Clinical features and dynamics of viral load in imported and non-imported patients with COVID-19. Int J Infect Dis. 2020; doi: 10.1016/j.ijid.2020.03.022

144. Xu X-W, Wu X-X, Jiang X-G, Xu K-J, Ying L-J, Ma C-L, et al. Clinical findings in a group of patients infected with the 2019 novel coronavirus (SARS-Cov-2) outside of Wuhan, China: retrospective case series. BMJ. 2020; 368(doi: 10.1136/bmj.m606

145. Xu Y, Li X, Zhu B, Liang H, Fang C, Gong Y, et al. Characteristics of pediatric SARS-CoV-2 infection and potential evidence for persistent fecal viral shedding. Nat Med. 2020; 26(502–5. doi: 10.1038/s41591-020-0817-4

146. Xu Y, Li Y-r, Zeng Q, Lu Z-b, Li Y-z, Wu W, et al. Clinical characteristics of SARS-CoV-2 pneumonia compared to controls in Chinese Han population. medRxiv. 2020; doi: 10.1101/2020.03.08.20031658

147. Xu Y-H, Dong J-H, An W-M, Lv X-Y, Yin X-P, Zhang J-Z, et al. Clinical and computed tomographic imaging features of novel coronavirus pneumonia caused by SARS-CoV-2. J Infect. 2020; doi: 10.1016/j.jinf.2020.02.017

148. Xu Z, Wu W, Jin Y, Pan A. Key Points of Clinical and CT Imaging Features of 2019 Novel Coronavirus (2019-nCoV) Imported Pneumonia Based On 21 Cases Analysis. medRxiv. 2020; doi: 10.1101/2020.03.03.20030775

149. Yan S, Song X, Lin F, Zhu H, Wang X, Li M, et al. Clinical Characteristics of Coronavirus Disease 2019 in Hainan, China. medRxiv. 2020; doi: 10.1101/2020.03.19.20038539

150. Yang W, Cheng Z, Cao Q, Yang Z, Zhou H, Qin L, et al. Clinical Features and Computed Tomographic Manifestations of Patients with 2019 Novel Coronavirus Pneumonia: A Single Center Study in Shanghai, China. SSRN. 2020; doi: 10.2139/ssrn.3534223

151. Yang X, Yu Y, Xu J, Shu H, Liu H, Wu Y, et al. Clinical course and outcomes of critically ill patients with SARS-CoV-2 pneumonia in Wuhan, China: a single-centered, retrospective, observational study. Lancet Respir Med. 2020; doi: 10.1016/S2213-2600(20)30079-5

152. Yang P, Ding Y, Xu Z, Pu R, Li P, Yan J, et al. Epidemiological and clinical features of COVID-19 patients with and without pneumonia in Beijing, China. medRxiv. 2020; doi: 10.1101/2020.02.28.20028068

153. Yang S, Shi Y, Lu H, Xu J, Li F, Qian Z, et al. Clinical and CT features of early-stage patients with COVID-19: a retrospective analysis of imported cases in Shanghai, China. Eur Respir J. 2020; doi: 10.1183/13993003.00407-2020

154. Yao T, Gao Y, Cui Q, Shen J, Peng B, Chen Y, et al. Clinical Characteristics of 55 Cases of Deaths with COVID-19 Pneumonia in Wuhan, China: Retrospective Case Series. SSRN. 2020; doi: 10.2139/ssrn.3550019

155. Young BE, Ong SWX, Kalimuddin S, Low JG, Tan SY, Loh J, et al. Epidemiologic features and clinical course of patients infected with SARS-CoV-2 in Singapore. JAMA. 2020; doi: 10.1001/jama.2020.3204

156. Yu N, Li W, Kang Q, Xiong Z, Wang S, Lin X, et al. Clinical features and obstetric and neonatal outcomes of pregnant patients with COVID-19 in Wuhan, China: a retrospective, single-centre, descriptive study. Lancet Infect Dis. 2020; doi: 10.1016/S1473-3099(20)30176-6

157. Yu F, Yan L, Wang N, Yang S, Wang L, Tang Y, et al. Quantitative detection and viral load analysis of SARS-CoV-2 in infected patients. Clin Infect Dis. 2020; doi: 10.1093/cid/ciaa345/5812997

158. Yu H, Cai Q, Dai X, Liu X, Sun H. The clinical and epidemiological features and hints of 82 confirmed COVID-19 pediatric cases aged 0-16 in Wuhan, China. medRxiv. 2020; doi: 10.1101/2020.03.15.20036319

159. Yuan J, Kou S, Liang Y, Zeng J, Pan Y, Liu L. PCR Assays Turned Positive in 25 Discharged COVID-19 Patients. Clin Infect Dis. 2020; doi: 10.1093/cid/ciaa398

160. Yuan M, Yin W, Tao Z, Tan W, Hu Y. Association of radiologic findings with mortality of patients infected with 2019 novel coronavirus in Wuhan, China. PLoS One. 2020; 15(3):e0230548. doi: 10.1371/journal.pone.0230548

161. Yuan J, Zou R, Zeng L, Kou S, Lan J, Li X, et al. The correlation between viral clearance and biochemical outcomes of 94 COVID-19 infected discharged patients. Inflamm Res. 2020; doi: 10.1007/s00011-020-01342-0

162. Li Y, Wang W, Lei Y, Zhang B, Yang J, Hu J, et al. Comparison of the clinical characteristics between RNA positive and negative patients clinically diagnosed with 2019 novel coronavirus pneumonia. Zhonghua Jie He He Hu Xi Za Zhi. 2020; 43(1-10. doi: 10.3760/cma.j.cn112147-20200214-00095

163. Zeng L, Li J, Liao M, Hua R, Huang P, Zhang M, et al. Risk assessment of progression to severe conditions for patients with COVID-19 pneumonia: a single-center retrospective study. medRxiv. 2020; doi: 10.1101/2020.03.25.20043166

164. Zhang B, Zhou X, Qiu Y, Feng F, Feng J, Jia Y, et al. Clinical characteristics of 82 death cases with COVID-19. medRxiv. 2020; doi: 10.1101/2020.02.26.20028191

165. Zhang F, He L, Ouyang Y, Gong J, Li X, Wei Y, et al. Clinical Features of 81 Hospitalized Patients with 2019 Novel Coronavirus-Infected Pneumonia in Jingzhou, China: A Descriptive Study. SSRN. 2020; doi: 10.2139/ssrn.3544834

166. Zhang J-j, Dong X, Cao Y-y, Yuan Y-d, Yang Y-b, Yan Y-q, et al. Clinical characteristics of 140 patients infected with SARS‐CoV‐2 in Wuhan, China. Allergy. 2020; doi: 10.1111/all.14238

167. Zhang J, Liu Z-H, Luo X-H, Xi Y, Ren Z-Q, Li Y-N, et al. Clinical Hallmarks of 13 COVID-19 Patients Revealing SAA Biomarker. SSRN. 2020; doi: 10.2139/ssrn.3546066

168. Zhang J, Wang S, Xue Y. Fecal specimen diagnosis 2019 novel coronavirus–infected pneumonia. J Med Virol. 2020; 92(6):680-2. doi: 10.1002/jmv.25742

169. Zhang J, Yang S, Xu Y, Liu J, Guo J, Tian S, et al. Epidemiological and Clinical Characteristics of COVID-19 Infection Outside Wuhan, China: A Multicenter Study. SSRN. 2020; doi: 10.2139/ssrn.3546040

170. Zhang G, Hu C, Luo L, Fang F, Chen Y, Li J, et al. Clinical features and outcomes of 221 patients with COVID-19 in Wuhan, China. medRxiv. 2020; doi: 10.1101/2020.03.02.20030452

171. Zhang X, Cai H, Hu J, Lian J, Gu J, Zhang S, et al. Epidemiological, clinical characteristics of cases of SARS-CoV-2 infection with abnormal imaging findings. Int J Infect Dis. 2020; doi: 10.1016/j.ijid.2020.03.040

172. Zhang Y, Chen R, Wang J, Gong Y, Zhou Q, Cheng H-h, et al. Anaesthetic managment and clinical outcomes of parturients with COVID-19: a multicentre, retrospective, propensity score matched cohort study. medRxiv. 2020; doi: 10.1101/2020.03.24.20042176

173. Zhang Y. Gastrointestinal tract symptoms in coronavirus disease 2019: Analysis of clinical symptoms in adult patients. medRxiv. 2020; doi: 10.1101/2020.03.23.20040279

174. Zhang L, Zhu F, Xie L, Wang C, Wang J, Chen R, et al. Clinical characteristics of COVID-19-infected cancer patients: A retrospective case study in three hospitals within Wuhan, China. Ann Oncol. 2020; doi: 10.1016/j.annonc.2020.03.296

175. Zhang L, Sun W, Chen L, Wang Q, Liu Y, Zhao S, et al. Clinical Features and a Simple Model for Predicting the Mortality of Coronavirus Disease 2019 Patients on Admission. SSRN. 2020; doi: 10.2139/ssrn.3551386

176. Zhang G, Zhang J, Wang B, Zhu X, Wang Q, Qiu S. Analysis of clinical characteristics and laboratory findings of 95 cases of 2019 novel coronavirus pneumonia in Wuhan, China: a retrospective analysis. Respir Res. 2020; 21(1):1-10. doi: 10.1186/s12931-020-01338-8

177. Zhao D, Yao F, Wang L, Zheng L, Gao Y, Ye J, et al. A comparative study on the clinical features of COVID-19 pneumonia to other pneumonias. Clin Infect Dis. 2020; doi: 10.1093/cid/ciaa247/5803302

178. Zhao S, Ling K, Yan H, Zhong L, Peng X, Yao S, et al. Anesthetic management of patients with suspected 2019 novel coronavirus infection during emergency procedures. J Cardiothorac Vasc Anesth. 2020; doi: 10.1053/j.jvca.2020.02.039

179. Zhao W, He L, Xie X, Liu J. The Viral Load of 2019 Novel Coronavirus (COVID-19) has the potential to predict the clinical outcomes. SSRN. 2020; doi: 10.2139/ssrn.3546047

180. Zhao W, Yu S, Zha X, Wang N, Pang Q, Li T, et al. Clinical characteristics and durations of hospitalized patients with COVID-19 in Beijing: a retrospective cohort study. medRxiv. 2020; doi: 10.1101/2020.03.13.20035436

181. Zhao W, Zhong Z, Yu Q, Xie X, Liu J. Clinical Outcomes of Patients with 2019-nCoV: A Preliminary Summary. SSRN. 2020; doi: 10.2139/ssrn.3542167

182. Zhao W, Zhong Z, Xie X, Yu Q, Liu J. Relation between chest CT findings and clinical conditions of coronavirus disease (COVID-19) pneumonia: a multicenter study. Am J Roentgenol. 2020; doi: 10.2214/AJR.20.22976

183. Zhao Z, Xie J, Yin M, Yang Y, He H, Jin T, et al. Clinical and Laboratory Profiles of 75 Hospitalized Patients with Novel Coronavirus Disease 2019 in Hefei, China. medRxiv. 2020; doi: 10.1101/2020.03.01.20029785

184. Zhao X, Liu B, Yu Y, Wang X, Du Y, Gu J, et al. The characteristics and clinical value of chest CT images of novel coronavirus pneumonia. Clin Radiol. 2020; doi: 10.1016/j.crad.2020.03.002

185. Zhao H, Lu J, Hongjun Z, Lu X, Li T, Rao B, et al. A New Features of SARS-CoV-2 Infection in Wenzhou, China. Research Square. 2020; doi: 10.21203/rs.2.23945/v1

186. Zheng C, Wang J, Guo H, Lu Z, Ma Y, Zhu Y, et al. Risk-adapted Treatment Strategy For COVID-19 Patients. Int J Infect Dis. 2020; doi: 10.1016/j.ijid.2020.03.047

187. Zheng F, Liao C, Fan Q-h, Chen H-b, Zhao X-g, Xie Z-g, et al. Clinical characteristics of children with coronavirus disease 2019 in Hubei, China. Curr Med Sci. 2020; doi: 10.1007/s11596-020-2172-6

188. Zhong Q, Liu YY, Luo Q, Zou YF, Jiang HX, Li H, et al. Spinal anaesthesia for patients with coronavirus disease 2019 and possible transmission rates in anaesthetists: retrospective, single-centre, observational cohort study. Br J Anaesth. 2020; doi: 10.1016/j.bja.2020.03.007

189. Zhou F, Yu T, Du R, Fan G, Liu Y, Liu Z, et al. Clinical course and risk factors for mortality of adult inpatients with COVID-19 in Wuhan, China: a retrospective cohort study. Lancet. 2020; doi: 10.1016/S0140-6736(20)30566-3

190. Zhou S, Wang Y, Zhu T, Xia L. CT features of coronavirus disease 2019 (COVID-19) pneumonia in 62 patients in Wuhan, China. Am J Roentgenol. 2020; doi: 10.2214/AJR.20.22975

191. Zhou Z, Zhao N, Shu Y, Han S, Chen B, Shu X. Effect of gastrointestinal symptoms on patients infected with COVID-19. Gastroenterology. 2020; doi: 10.1053/j.gastro.2020.03.020

192. Zhou Z, Guo D, Li C, Fang Z, Chen L, Yang R, et al. Coronavirus disease 2019: initial chest CT findings. Eur Radiol. 2020; doi: 10.1007/s00330-020-06816-7

193. Zhou Y, Yang G, Feng K, Huang H, Yun Y, Mou X, et al. Clinical features and chest CT findings of coronavirus disease 2019 in infants and young children. Zhongguo Dang Dai Er Ke Za Zhi. 2020; 22(3):215-20.

194. Zhu W, Xie K, Lu H, Xu L, Zhou S, Fang S. Initial clinical features of suspected Coronavirus Disease 2019 in two emergency departments outside of Hubei, China. J Med Virol. 2020; doi: 10.1002/jmv.25763

195. Zhu X, Yuan W, Huang K, Wang Q, Yao S, Lu W, et al. Clinical Features and Short-Term Outcomes of 114 Elderly Patients with COVID-19 in Wuhan, China: A Single-Center, Retrospective, Observational Study. SSRN. 2020; doi: 10.2139/ssrn.3548774

196. Zhu Y, Gao Z-H, Liu Y-L, Xu D-Y, Guan T-M, Li Z-P, et al. Clinical and CT imaging features of 2019 novel coronavirus disease (COVID-19). J Infect. 2020; doi: 10.1016/j.jinf.2020.02.022

197. Zhu H, Wang L, Fang C, Peng S, Zhang L, Chang G, et al. Clinical analysis of 10 neonates born to mothers with 2019-nCoV pneumonia. Transl Pediatr. 2020; 9(1):51-60. doi: 10.21037/tp.2020.02.06
