# Supplementary material for: Prevalence and characteristics of fever in adult and paediatric patients with coronavirus disease 2019 (COVID-19): A systematic review and meta-analysis of 17515 patients
Source: PLoS One. 2021 Apr 6;16(4):e0249788. doi: 10.1371/journal.pone.0249788 (PMC8023501; doi:10.1371/journal.pone.0249788)
Supplement: S3 Table — (DOCX) [file pone.0249788.s019.docx]

| **S3 Table. Quality assessment of the included cross-sectional studies.** | | | | | | | | | | |
| --- | --- | --- | --- | --- | --- | --- | --- | --- | --- | --- |
| **No.** | **Study ID** | **Questions assessing included cross-sectional studies** | | | | | | | | **Yes (%)** |
|  |  | **1** | **2** | **3** | **4** | **5** | **6** | **7** | **8** |  |
| 1 | Ai 2020 | Y | Y | Y | Y | N | N | Y | Y | 75·0 |
| 2 | Bhatraju 2020 | Y | Y | Y | Y | N | N | N | Y | 62·5 |
| 3 | Cao 2020a | Y | Y | Y | Y | N | N | Y | Y | 75·0 |
| 4 | Cao 2020b | Y | Y | Y | Y | N | N | Y | Y | 75·0 |
| 5 | Chang 2020 | Y | Y | N | Y | N | N | N | N | 37·5 |
| 6 | Chang 2020a | Y | Y | N | Y | N | N | N | N | 37·5 |
| 7 | Chen 2020 | Y | Y | Y | Y | N | N | Y | Y | 75·0 |
| 8 | Chen 2020a | Y | Y | Y | Y | N | N | Y | Y | 75·0 |
| 9 | Chen 2020b | Y | Y | Y | Y | Y | Y | Y | Y | 100·0 |
| 10 | Chen 2020c | Y | Y | Y | Y | N | N | Y | Y | 75·0 |
| 11 | Chen 2020d | Y | Y | Y | Y | N | N | N | Y | 62·5 |
| 12 | Chen 2020e | Y | Y | Y | Y | N | N | Y | Y | 75·0 |
| 13 | Chen 2020f | Y | Y | Y | Y | N | N | N | Y | 62·5 |
| 14 | Chen 2020h | Y | Y | Y | Y | N | N | Y | Y | 75·0 |
| 15 | Chen 2020i | Y | Y | Y | N | N | N | Y | Y | 62·5 |
| 16 | Chen 2020j | Y | Y | Y | Y | N | N | Y | Y | 75·0 |
| 17 | Chen 2020k | Y | Y | Y | Y | N | N | Y | Y | 75·0 |
| 18 | Chen 2020l | Y | Y | Y | Y | N | N | N | Y | 62·5 |
| 19 | Cheng 2020a | Y | Y | Y | Y | N | N | Y | Y | 75·0 |
| 20 | Chu 2020 | Y | Y | Y | Y | N | N | N | Y | 62·5 |
| 21 | Chuan 2020 | Y | Y | Y | Y | N | N | N | Y | 62·5 |
| 22 | Cui 2020 | Y | Y | Y | Y | N | N | N | Y | 62·5 |
| 23 | Deng 2020 | Y | Y | Y | Y | N | N | N | Y | 62·5 |
| 24 | Du 2020 | Y | Y | Y | Y | Y | Y | Y | Y | 100·0 |
| 25 | Du 2020a | Y | Y | Y | Y | N | N | Y | Y | 75·0 |
| 26 | Easom 2020 | Y | Y | Y | Y | N | N | Y | Y | 75·0 |
| 27 | Fan 2020 | Y | Y | Y | Y | N | N | Y | Y | 75·0 |
| 28 | Fan 2020a | Y | Y | Y | Y | N | N | Y | Y | 75·0 |
| 29 | Feng 2020 | Y | Y | Y | Y | Y | N | N | Y | 75·0 |
| 30 | Fu 2020 | Y | Y | Y | Y | N | N | N | Y | 62·5 |
| 31 | Guan 2020 | Y | Y | Y | Y | N | N | Y | Y | 75·0 |
| 32 | Han 2020 | Y | Y | Y | Y | N | N | Y | N | 62·5 |
| 33 | Hou 2020 | Y | Y | Y | Y | N | N | Y | Y | 75·0 |
| 34 | Hu 2020 | Y | Y | Y | Y | N | N | Y | Y | 75·0 |
| 35 | Hu 2020a | Y | Y | N | Y | N | N | N | Y | 50·0 |
| 36 | Hu 2020b | Y | Y | Y | Y | Y | Y | Y | Y | 100·0 |
| 37 | Huang 2020 | Y | Y | Y | Y | N | N | Y | Y | 75·0 |
| 38 | Huang 2020a | Y | Y | Y | Y | N | N | N | Y | 62·5 |
| 39 | Huang 2020b | Y | Y | Y | Y | Y | N | N | Y | 75·0 |
| 40 | Huang 2020c | Y | Y | N | Y | N | N | N | Y | 50·0 |
| 41 | Huang 2020d | Y | Y | N | Y | N | N | Y | N | 50·0 |
| 42 | Ji 2020 | Y | Y | Y | Y | N | N | Y | Y | 75·0 |
| 43 | Jin 2020 | Y | Y | Y | Y | Y | Y | Y | Y | 100·0 |
| 44 | Lei 2020 | Y | Y | Y | Y | N | N | N | Y | 62·5 |
| 45 | Lei 2020a | Y | Y | Y | Y | N | N | Y | Y | 75·0 |
| 46 | Lei 2020b | Y | Y | Y | Y | N | N | N | Y | 62·5 |
| 47 | Li 2020 | Y | Y | N | Y | N | N | N | Y | 50·0 |
| 48 | Li 2020a | Y | Y | Y | Y | N | N | Y | Y | 75·0 |
| 49 | Li 2020b | Y | Y | Y | Y | Y | N | N | Y | 75·0 |
| 50 | Li 2020c | Y | Y | Y | Y | Y | Y | N | Y | 87·5 |
| 51 | Li 2020d | Y | Y | Y | Y | Y | Y | N | Y | 87·5 |
| 52 | Li 2020e | Y | Y | Y | Y | N | N | Y | Y | 75·0 |
| 53 | Li 2020f | Y | Y | Y | Y | N | N | Y | Y | 75·0 |
| 54 | Lian 2020 | Y | Y | Y | Y | N | N | Y | Y | 75·0 |
| 55 | Liang 2020 | Y | Y | Y | Y | N | N | Y | Y | 75·0 |
| 56 | Liao 2020 | Y | Y | Y | Y | N | N | N | Y | 62·5 |
| 57 | Liu 2020a | Y | Y | Y | Y | N | N | N | Y | 62·5 |
| 58 | Liu 2020b | Y | Y | Y | Y | N | N | Y | Y | 75·0 |
| 59 | Liu 2020c | Y | Y | Y | Y | N | N | Y | Y | 75·0 |
| 60 | Liu 2020e | Y | Y | Y | Y | N | N | N | Y | 62·5 |
| 61 | Liu 2020f | Y | Y | Y | Y | Y | Y | Y | Y | 100·0 |
| 62 | Liu 2020g | Y | Y | Y | Y | Y | Y | N | Y | 87·5 |
| 63 | Liu 2020h | Y | Y | Y | Y | N | N | Y | Y | 75·0 |
| 64 | Liu 2020i | Y | Y | Y | Y | N | N | N | Y | 62·5 |
| 65 | Liu 2020k | Y | Y | Y | Y | N | N | N | Y | 62·5 |
| 66 | Liu 2020l | Y | Y | Y | Y | Y | Y | Y | Y | 100·0 |
| 67 | Liu 2020n | Y | Y | Y | Y | N | N | N | N | 50·0 |
| 68 | Liu 2020p | Y | Y | N | Y | N | N | N | Y | 50·0 |
| 69 | Lu 2020 | Y | Y | Y | Y | Y | Y | Y | Y | 100·0 |
| 70 | Lu 2020b | Y | Y | N | Y | N | N | N | Y | 50·0 |
| 71 | Luo 2020 | Y | Y | Y | N | N | N | N | Y | 50·0 |
| 72 | Miao 2020 | Y | Y | N | Y | N | N | Y | Y | 62·5 |
| 73 | Min 2020 | Y | Y | N | Y | N | N | Y | Y | 62·5 |
| 74 | Mo 2020 | Y | Y | N | Y | Y | Y | Y | Y | 87·5 |
| 75 | Nie 2020 | Y | Y | Y | Y | N | N | N | Y | 62·5 |
| 76 | Pan 2020 | Y | Y | Y | Y | N | N | Y | Y | 75·0 |
| 77 | Pung 2020 | Y | Y | Y | Y | N | N | N | Y | 62·5 |
| 78 | Qi 2020 | Y | Y | Y | Y | N | N | Y | Y | 75·0 |
| 79 | Qian 2020 | Y | Y | Y | Y | N | N | Y | Y | 75·0 |
| 80 | Qin 2020 | Y | Y | Y | Y | N | N | N | Y | 62·5 |
| 81 | Qin 2020a | Y | Y | Y | Y | N | N | N | Y | 62·5 |
| 82 | Qiu 2020a | Y | Y | Y | Y | N | N | Y | Y | 75·0 |
| 83 | Shi 2020 | Y | Y | Y | Y | N | N | Y | Y | 75·0 |
| 84 | Shi 2020a | Y | Y | Y | N | Y | Y | Y | Y | 87·5 |
| 85 | Shu 2020 | Y | Y | Y | Y | N | N | N | Y | 62·5 |
| 86 | Song 2020 | Y | Y | N | Y | N | N | Y | Y | 62·5 |
| 87 | Su 2020 | Y | Y | N | Y | N | N | N | N | 37·5 |
| 88 | Tang 2020 | Y | Y | Y | Y | N | N | N | Y | 62·5 |
| 89 | Tian 2020 | Y | Y | Y | Y | N | N | Y | Y | 75·0 |
| 90 | Tian 2020a | Y | Y | Y | Y | N | N | Y | Y | 75·0 |
| 91 | Wan 2020 | Y | Y | Y | Y | N | N | Y | Y | 75·0 |
| 92 | Wang 2020 | Y | Y | N | Y | N | N | Y | Y | 62·5 |
| 93 | Wang 2020a | Y | Y | Y | Y | U | N | Y | Y | 75·0 |
| 94 | Wang 2020b | Y | Y | N | Y | N | N | Y | Y | 62·5 |
| 95 | Wang 2020c | Y | Y | Y | Y | N | N | N | N | 50·0 |
| 96 | Wang 2020e | Y | Y | Y | Y | N | N | N | Y | 62·5 |
| 97 | Wang 2020f | Y | Y | Y | Y | Y | Y | Y | Y | 100·0 |
| 98 | Wang 2020g | Y | Y | Y | Y | N | N | Y | Y | 75·0 |
| 99 | Wang 2020h | Y | Y | Y | Y | N | N | Y | Y | 75·0 |
| 100 | Wang 2020i | Y | Y | Y | Y | Y | Y | N | Y | 87·5 |
| 101 | Wang 2020j | Y | Y | N | Y | Y | Y | N | Y | 75·0 |
| 102 | Wei 2020 | Y | Y | Y | Y | N | N | N | Y | 62·5 |
| 103 | Wei 2020a | Y | Y | Y | Y | N | N | N | Y | 62·5 |
| 104 | Wen 2020 | Y | Y | Y | Y | Y | Y | Y | Y | 100·0 |
| 105 | Wu 2020 | Y | Y | Y | Y | N | N | N | Y | 62·5 |
| 106 | Wu 2020a | Y | Y | Y | Y | Y | N | N | Y | 75·0 |
| 107 | Wu 2020b | Y | Y | Y | Y | N | N | Y | Y | 75·0 |
| 108 | Wu 2020c | Y | Y | Y | Y | N | N | Y | Y | 75·0 |
| 109 | Xia 2020 | Y | Y | Y | Y | N | N | Y | N | 62·5 |
| 110 | Xiong 2020 | Y | Y | Y | Y | Y | Y | Y | Y | 100·0 |
| 111 | Xu 2020 | Y | Y | Y | Y | N | N | Y | Y | 75·0 |
| 112 | Xu 2020a | Y | Y | Y | Y | N | N | Y | Y | 75·0 |
| 113 | Xu 2020b | Y | Y | Y | Y | N | N | Y | Y | 75·0 |
| 114 | Xu 2020c | Y | Y | Y | Y | N | N | Y | Y | 75·0 |
| 115 | Xu 2020e | Y | Y | Y | Y | U | N | N | Y | 62·5 |
| 116 | Xu 2020f | Y | Y | Y | Y | N | N | Y | Y | 75·0 |
| 117 | Xu 2020g | Y | Y | Y | N | N | N | N | Y | 50·0 |
| 118 | Yan 2020 | Y | Y | Y | Y | N | N | Y | Y | 75·0 |
| 119 | Yang 2020 | Y | Y | Y | Y | N | N | Y | Y | 75·0 |
| 120 | Yang 2020a | Y | Y | Y | N | N | N | N | Y | 50·0 |
| 121 | Yang 2020b | Y | Y | Y | Y | Y | Y | Y | Y | 100·0 |
| 122 | Yang 2020c | Y | Y | N | Y | Y | Y | N | Y | 75·0 |
| 123 | Yao 2020 | Y | Y | Y | Y | N | N | N | Y | 62·5 |
| 124 | Yu 2020 | Y | Y | Y | Y | N | N | N | Y | 62·5 |
| 125 | Yu 2020a | Y | Y | N | Y | N | N | N | N | 37·5 |
| 126 | Yu 2020b | Y | Y | N | Y | N | N | N | Y | 50·0 |
| 127 | Yuan 2020 | Y | Y | N | Y | N | N | N | Y | 50·0 |
| 128 | Yuan 2020a | Y | Y | Y | Y | N | N | N | Y | 62·5 |
| 129 | Yuan 2020b | Y | Y | N | Y | Y | Y | N | Y | 75·0 |
| 130 | Yuanyuan 2020 | Y | Y | Y | Y | N | N | Y | Y | 75·0 |
| 131 | Zeng 2020 | Y | Y | Y | Y | Y | Y | Y | Y | 100·0 |
| 132 | Zhang 2020 | Y | Y | N | Y | N | N | Y | Y | 62·5 |
| 133 | Zhang 2020a | Y | Y | Y | Y | N | N | N | Y | 62·5 |
| 134 | Zhang 2020b | Y | Y | Y | Y | N | N | N | Y | 62·5 |
| 135 | Zhang 2020e | Y | Y | Y | N | N | N | Y | Y | 62·5 |
| 136 | Zhang 2020f | Y | Y | Y | Y | N | N | Y | Y | 75·0 |
| 137 | Zhang 2020g | Y | Y | Y | Y | Y | Y | N | Y | 87·5 |
| 138 | Zhang 2020i | Y | Y | Y | Y | Y | Y | N | Y | 87·5 |
| 139 | Zhang 2020k | Y | Y | Y | Y | Y | Y | N | Y | 87·5 |
| 140 | Zhang 2020l | Y | Y | Y | Y | N | N | Y | Y | 75·0 |
| 141 | Zhao 2020 | Y | Y | Y | Y | N | N | N | Y | 62·5 |
| 142 | Zhao 2020a | Y | Y | N | N | N | N | Y | Y | 50·0 |
| 143 | Zhao 2020b | Y | Y | Y | Y | N | N | N | Y | 62·5 |
| 144 | Zhao 2020d | Y | Y | Y | Y | N | N | N | Y | 62·5 |
| 145 | Zhao 2020e | Y | Y | Y | Y | N | N | N | Y | 62·5 |
| 146 | Zhao 2020f | Y | Y | Y | Y | N | N | Y | Y | 75·0 |
| 147 | Zhao 2020g | Y | Y | N | Y | N | N | Y | N | 50·0 |
| 148 | Zhao 2020h | Y | Y | Y | Y | N | N | N | Y | 62·5 |
| 149 | Zheng 2020 | Y | Y | N | Y | N | N | N | Y | 50·0 |
| 150 | Zheng 2020a | Y | Y | Y | Y | N | N | N | Y | 62·5 |
| 151 | Zhou 2020a | Y | Y | N | Y | N | N | N | Y | 50·0 |
| 152 | Zhou 2020b | Y | Y | Y | Y | N | N | N | Y | 62·5 |
| 153 | Zhou 2020c | Y | Y | Y | Y | N | N | Y | Y | 75·0 |
| 154 | Zhu 2020 | Y | Y | Y | Y | N | N | Y | Y | 75·0 |
| 155 | Zhu 2020a | Y | Y | Y | Y | Y | Y | N | Y | 87·5 |
| 156 | Zhu 2020b | Y | Y | N | Y | N | N | Y | N | 50·0 |
| 1. Were the criteria for inclusion in the sample clearly defined? 2. Were the study subjects and the setting described in detail? 3. Was the exposure measured in a valid and reliable way? 4. Were objective, standard criteria used for measurement of the condition? 5. Were confounding factors identified? 6. Were strategies to deal with confounding factors stated? 7. Were the outcomes measured in a valid and reliable way? 8. Was appropriate statistical analysis used? Y=Yes; N=No; U=Unclear. | | | | | | | | | | |
