# Supplementary material for: Prevalence and characteristics of fever in adult and paediatric patients with coronavirus disease 2019 (COVID-19): A systematic review and meta-analysis of 17515 patients
Source: PLoS One. 2021 Apr 6;16(4):e0249788. doi: 10.1371/journal.pone.0249788 (PMC8023501; doi:10.1371/journal.pone.0249788)
Supplement: S4 Table — (DOCX) [file pone.0249788.s020.docx]

| **S4 Table. Quality assessment of the included cohort studies.** | | | | | | | | | | | | | |
| --- | --- | --- | --- | --- | --- | --- | --- | --- | --- | --- | --- | --- | --- |
| **No.** | **Study ID** | **Questions assessing included cohort studies** | | | | | | | | | | | **Yes (%)** |
|  |  | **1** | **2** | **3** | **4** | **5** | **6** | **7** | **8** | **9** | **10** | **11** |  |
| 1 | Cheng 2020 | Y | Y | Y | Y | Y | Y | Y | U | Y | NA | Y | 90·0 |
| 2 | Guan 2020a | Y | Y | Y | Y | Y | Y | Y | N | Y | NA | Y | 90·0 |
| 3 | He 2020 | Y | Y | Y | Y | Y | Y | Y | Y | Y | N | Y | 90·0 |
| 4 | Liu 2020o | Y | Y | Y | Y | Y | Y | Y | Y | Y | NA | Y | 100.0 |
| 5 | Lu 2020a | Y | Y | Y | U | N | Y | Y | Y | Y | NA | Y | 80·0 |
| 6 | Mao 2020 | Y | Y | Y | Y | Y | Y | Y | Y | Y | NA | Y | 100·0 |
| 7 | Qiu 2020 | Y | Y | Y | N | N | Y | Y | Y | Y | NA | Y | 90·0 |
| 8 | To 2020 | Y | Y | Y | Y | N | Y | Y | Y | Y | NA | Y | 90·0 |
| 9 | Zhang 2020j | Y | Y | Y | Y | Y | Y | Y | Y | Y | NA | Y | 100·0 |
| 10 | Zhao 2020c | Y | Y | Y | Y | Y | Y | Y | Y | Y | NA | Y | 100·0 |
| 11 | Zhong 2020 | Y | Y | Y | N | N | Y | Y | N | Y | NA | Y | 70·0 |
| 12 | Zhou 2020 | Y | Y | Y | Y | Y | Y | Y | Y | Y | NA | Y | 100·0 |
| 1. Were the two groups similar and recruited from the same population? 2. Were the exposures measured similarly to assign people to both exposed and unexposed groups? 3. Was the exposure measured in a valid and reliable way? 4. Were confounding factors identified? 5. Were strategies to deal with confounding factors stated? 6. Were the groups/participants free of the outcome at the start of the study (or at the moment of exposure)? 7. Were the outcomes measured in a valid and reliable way? 8. Was the follow up time reported and sufficient to be long enough for outcomes to occur? 9. Was follow up complete, and if not, were the reasons to loss to follow up described and explored? 10. Were strategies to address incomplete follow up utilized? 11. Was appropriate statistical analysis used? Y=Yes; N=No; U=Unclear; NA: Not applicable. | | | | | | | | | | | | | |
