# Supplementary material for: Prevalence and characteristics of fever in adult and paediatric patients with coronavirus disease 2019 (COVID-19): A systematic review and meta-analysis of 17515 patients
Source: PLoS One. 2021 Apr 6;16(4):e0249788. doi: 10.1371/journal.pone.0249788 (PMC8023501; doi:10.1371/journal.pone.0249788)
Supplement: S5 Table — (DOCX) [file pone.0249788.s021.docx]

| **S5 Table. Quality assessment of the included case series.** | | | | | | | | | | | | |
| --- | --- | --- | --- | --- | --- | --- | --- | --- | --- | --- | --- | --- |
| **No.** | **Study ID** | **Questions assessing included case series** | | | | | | | | | | **Yes (%)** |
|  |  | **1** | **2** | **3** | **4** | **5** | **6** | **7** | **8** | **9** | **10** |  |
| 1 | Chen 2020g | Y | Y | Y | Y | Y | Y | Y | Y | Y | Y | 100·0 |
| 2 | Chen 2020m | Y | Y | Y | Y | Y | Y | Y | Y | Y | N | 90·0 |
| 3 | Chung 2020 | Y | Y | N | Y | Y | Y | Y | Y | Y | N | 80·0 |
| 4 | Diao 2020 | Y | Y | N | Y | Y | Y | Y | Y | Y | N | 80·0 |
| 5 | Ding 2020 | Y | Y | Y | N | N | Y | Y | Y | Y | Y | 80·0 |
| 6 | Feng 2020a | Y | Y | Y | Y | Y | Y | Y | Y | Y | Y | 100·0 |
| 7 | Han 2020a | Y | Y | Y | Y | Y | Y | Y | Y | N | Y | 90·0 |
| 8 | Jiehao 2020 | Y | Y | Y | Y | Y | Y | Y | Y | Y | N | 90·0 |
| 9 | Lescure 2020 | Y | Y | Y | Y | Y | Y | Y | Y | Y | N | 90·0 |
| 10 | Liu 2020d | Y | Y | N | Y | Y | Y | Y | Y | Y | Y | 90·0 |
| 11 | Liu 2020j | Y | Y | N | Y | Y | Y | Y | Y | Y | N | 80·0 |
| 12 | Liu 2020m | Y | Y | Y | Y | Y | Y | Y | Y | Y | Y | 100·0 |
| 13 | Lo 2020 | Y | Y | Y | Y | Y | Y | Y | Y | Y | Y | 100·0 |
| 14 | Sun 2020 | Y | Y | Y | Y | Y | Y | Y | Y | Y | N | 90·0 |
| 15 | Wang 2020d | Y | Y | Y | Y | Y | Y | Y | Y | Y | Y | 100·0 |
| 16 | Xie 2020 | Y | Y | N | Y | Y | Y | Y | Y | N | N | 70·0 |
| 17 | Xu 2020d | Y | Y | N | Y | Y | Y | Y | Y | Y | Y | 90·0 |
| 18 | Young 2020 | Y | Y | Y | Y | Y | Y | Y | Y | Y | N | 90·0 |
| 19 | Zhang 2020c | Y | Y | Y | Y | Y | Y | Y | Y | Y | Y | 100·0 |
| 20 | Zhang 2020d | Y | Y | N | Y | Y | Y | N | Y | Y | N | 70·0 |
| 21 | Zhou 2020d | Y | Y | Y | Y | Y | Y | Y | Y | Y | N | 90·0 |
| 22 | Zhu 2020c | Y | Y | Y | Y | Y | Y | Y | Y | Y | N | 90·0 |
| 1. Were there clear criteria for inclusion in the case series? 2. Was the condition measured in a standard, reliable way for all participants included in the case series? 3. Were valid methods used for identification of the condition for all participants included in the case series? 4. Did the case series have consecutive inclusion of participants? 5. Did the case series have complete inclusion of participants? 6. Was there clear reporting of the demographics of the participants in the study? 7. Was there clear reporting of clinical information of the participants? 8. Were the outcomes or follow up results of cases clearly reported? 9. Was there clear reporting of the presenting site(s)/clinic(s) demographic information? 10. Was statistical analysis appropriate? Y=Yes; N=No; U=Unclear. | | | | | | | | | | | | |
