# Supplementary material for: Prevalence and characteristics of fever in adult and paediatric patients with coronavirus disease 2019 (COVID-19): A systematic review and meta-analysis of 17515 patients
Source: PLoS One. 2021 Apr 6;16(4):e0249788. doi: 10.1371/journal.pone.0249788 (PMC8023501; doi:10.1371/journal.pone.0249788)
Supplement: S6 Table — (DOCX) [file pone.0249788.s022.docx]

| **S6 Table. Quality assessment of the included randomised controlled trials.** | | | | | | | | | | | | | | | | |
| --- | --- | --- | --- | --- | --- | --- | --- | --- | --- | --- | --- | --- | --- | --- | --- | --- |
| **No.** | **Study ID** | **Questions assessing included randomised controlled trials** | | | | | | | | | | | | | **Yes (%)** |  |
|  |  | **1** | **2** | **3** | **4** | **5** | **6** | **7** | **8** | **9** | **10** | **11** | **12** | **13** |  |  |
| 1 | Cao 2020 | Y | Y | Y | Y | N | N | Y | Y | Y | Y | Y | Y | Y | 84·6 |  |
| 2 | Chen 2020n | Y | U | Y | U | U | U | Y | Y | Y | Y | Y | Y | Y | 69·2 |  |
| 3 | Liu 2020 | N | U | Y | N | N | U | Y | Y | Y | Y | Y | Y | Y | 61·5 |  |
| 1. Was true randomization used for assignment of participants to treatment groups? 2. Was allocation to treatment groups concealed? 3. Were treatment groups similar at the baseline? 4. Were participants blind to treatment assignment? 5. Were those delivering treatment blind to treatment assignment? 6. Were outcomes assessors blind to treatment assignment? 7. Were treatment groups treated identically other than the intervention of interest? 8. Was follow up complete and if not, were differences between groups in terms of their follow up adequately described and analyzed? 9. Were participants analyzed in the groups to which they were randomized? 10. Were outcomes measured in the same way for treatment groups? 11. Were outcomes measured in a reliable way? 12. Was appropriate statistical analysis used? 13. Was the trial design appropriate, and any deviations from the standard RCT design (individual randomization, parallel groups) accounted for in the conduct and analysis of the trial? Y=Yes; N=No; U=Unclear. | | | | | | | | | | | | | | | | |
