# Supplementary material for: Prevalence and characteristics of fever in adult and paediatric patients with coronavirus disease 2019 (COVID-19): A systematic review and meta-analysis of 17515 patients
Source: PLoS One. 2021 Apr 6;16(4):e0249788. doi: 10.1371/journal.pone.0249788 (PMC8023501; doi:10.1371/journal.pone.0249788)
Supplement: S7 Table — (DOCX) [file pone.0249788.s023.docx]

| **S7 Table. Quality assessment of the included case-control studies.** | | | | | | | | | | | | |
| --- | --- | --- | --- | --- | --- | --- | --- | --- | --- | --- | --- | --- |
| **No.** | **Study ID** | **Questions assessing included case-control studies** | | | | | | | | | | **Yes (%)** |
|  |  | **1** | **2** | **3** | **4** | **5** | **6** | **7** | **8** | **9** | **10** |  |
| 1 | Tang 2020a | Y | Y | Y | Y | Y | Y | Y | Y | Y | Y | 100·0 |
| 2 | Cheng 2020b | Y | Y | Y | Y | Y | U | N | Y | Y | Y | 80·0 |
| 3 | Zhang 2020h | Y | Y | Y | N | Y | N | N | Y | Y | Y | 70·0 |
| 1. Were the groups comparable other than the presence of disease in cases or the absence of disease in controls? 2. Were cases and controls matched appropriately? 3. Were the same criteria used for identification of cases and controls? 4. Was exposure measured in a standard, valid and reliable way? 5. Was exposure measured in the same way for cases and controls? 6. Were confounding factors identified? 7. Were strategies to deal with confounding factors stated? 8. Were outcomes assessed in a standard, valid and reliable way for cases and controls? 9. Was the exposure period of interest long enough to be meaningful? 10. Was appropriate statistical analysis used? Y=Yes; N=No; U=Unclear. | | | | | | | | | | | | |
