# Supplementary material for: Prevalence and characteristics of fever in adult and paediatric patients with coronavirus disease 2019 (COVID-19): A systematic review and meta-analysis of 17515 patients
Source: PLoS One. 2021 Apr 6;16(4):e0249788. doi: 10.1371/journal.pone.0249788 (PMC8023501; doi:10.1371/journal.pone.0249788)
Supplement: S8 Table — (DOCX) [file pone.0249788.s024.docx]

| **S8 Table. Quality assessment of the included non-randomised experimental studies.** | | | | | | | | | | | |
| --- | --- | --- | --- | --- | --- | --- | --- | --- | --- | --- | --- |
| **No.** | **Study ID** | **Questions assessing included non-randomised experimental studies** | | | | | | | | | **Yes (%)** |
|  |  | **1** | **2** | **3** | **4** | **5** | **6** | **7** | **8** | **9** |  |
| 1 | Bian 2020 | Y | Y | N | Y | Y | N | Y | Y | Y | 84·6 |
| 1. Is it clear in the study what is the ‘cause’ and what is the ‘effect’ (i.e. there is no confusion about which variable comes first)? 2. Were the participants included in any comparisons similar? 3. Were the participants included in any comparisons receiving similar treatment/care, other than the exposure or intervention of interest? 4. Was there a control group? 5. Were there multiple measurements of the outcome both pre and post the intervention/exposure? 6. Was follow up complete and if not, were differences between groups in terms of their follow up adequately described and analyzed? 7. Were the outcomes of participants included in any comparisons measured in the same way? 8. Were outcomes measured in a reliable way? 9. Was appropriate statistical analysis used? Y=Yes; N=No; U=Unclear. | | | | | | | | | | | |
